# Disrupting the semantic network shifts activity to domain-general brain regions

**DOI:** 10.1038/s41467-026-79266-2

**Published:** 2026-11-05

**Authors:** Anna-Lisa Schuler, Andrea Bruera, Gesa Hartwigsen

**Affiliations:** 1 Research Group Cognition and Plasticity, Max Planck Institute for Human Cognitive and Brain Sciences, Leipzig, Germany; 2 Cognitive and Biological Psychology, Wilhelm Wundt Institute for Psychology, Leipzig University, Leipzig, Germany

**Keywords:** Language, Cognitive control, Human behaviour

## Abstract

Semantic processing relies on flexible interactions between specialized lexico-semantic regions and domain-general control networks, particularly under challenging conditions. Using transcranial magnetic stimulation interleaved with functional magnetic resonance imaging, we investigated acute network adaptation when the left posterior middle temporal gyrus—a core lexico-semantic region—was perturbed during lexico-semantic decisions in 25 healthy participants. High-intensity transcranial magnetic stimulation over the posterior middle temporal gyrus induced task-specific activity increases across semantic control and lexical retrieval areas, as well as remote domain-general regions. Critically, increased local activity at the stimulation site correlated with slower reaction times, whereas greater upregulation in domain-general frontal regions was associated with less disrupted semantic performance. These findings indicate that acute semantic disruption triggers immediate, flexible recruitment of domain-general control areas consistent with the maintenance of task performance, highlighting the brain’s capacity to dynamically adapt to interference. Our findings contribute to the current understanding of putative compensatory network-level mechanisms supporting lexico-semantic cognition.

## Abstract (web summary)

Disrupting a key language region prompts increased activity in widespread brain regions. Stronger engagement of frontal control regions helps preserve cognitive performance, revealing the brain’s capacity to adapt to interference in real time.

## Introduction

Fluent semantic processing is a key faculty for language comprehension and production, storing and retrieving long-term information from memory and drawing inferences from contextual knowledge. Previous neuroimaging, lesion and neurostimulation studies have provided insight into the underlying neural circuits in the human brain^1–5^. These studies demonstrate that challenging situations such as noisy listening conditions and demanding tasks increase the contribution of the semantic control network^6–8^. This network comprises, among other regions, the left anterior inferior frontal gyrus (aIFG) and posterior middle temporal gyrus (pMTG;^1,9–12^). While the aIFG is crucial for resolving high-demand or conflicting semantic conditions, the pMTG has been associated with the controlled retrieval of semantic concepts and lexical retrieval (e.g., refs. ^8,13–15^). Notably, semantic control requires both the controlled retrieval of semantic concepts and the ability to solve conflicting semantic information^1^. Other work demonstrated that higher cognitive demands require flexible interactions between regions involved in general semantic processing, semantic control regions and domain-general areas of the multiple demand network (MDN;^6,16^). The MDN is central to executive processing and supports functions such as task set maintenance, attentional allocation, and response conflict resolution.

Beyond increasing task demands, brain lesions in semantic areas often severely impair semantic cognition. Several studies showed that recovery of language function does not only rely on the specialized language network but also engages multiple demand regions (e.g., refs. ^17,18^), especially during early recovery from stroke-induced language impairments^19,20^. Accordingly, we have recently argued that focal damage to domain-specific semantic areas may be partially compensated for by flexible recruitment of domain-general areas of the MDN^21^. Such increased contributions may support maintenance of specialized functions under challenging conditions in young and older brains^22^ and recovery of function after lesions^20^.

Yet, despite the identification of network adaptation as a key mechanism for flexible adaptation to challenges, the underlying acute network dynamics during semantic and lexical processing remain unclear. A better understanding of immediate flexible adaptation mechanisms is key for identifying the neural signatures that distinguish successful from unsuccessful compensatory recruitment and determining how to enhance these adaptive processes. Bridging the gap between static neuroimaging results and dynamic real-time modulation of lexico-semantic cognition would allow insights into the dynamics of flexible functional network adaptation. Moreover, elucidating dynamic adaptation during lexico-semantic cognition may not only inform neurobiological models of lexico-semantic processing but also provide initial insights relevant to future neurostimulation approaches for patients with lexico-semantic impairments following brain lesions.

However, despite the clear benefits of mapping immediate network adaptation during lexico-semantic cognition, progress is hindered by limitations of current approaches. Lesion studies are inherently limited by patient-related factors and confounded by remote degeneration of cortical neurons, disconnection syndromes and reorganization processes (e.g., ref. ^23^). On the other hand, “virtual lesion” studies with transcranial magnetic stimulation (TMS) usually separate TMS and functional magnetic resonance imaging (fMRI) in time, allowing to map plastic after-effects of TMS in local and remote regions (e.g., ref. ^24^). While such studies have provided new insights into adaptive short-term reorganization of the language network (see ref. ^25^, for review), they cannot map immediate effects at the neural network level. Finally, behavioral "online" TMS studies have enabled focal, non-invasive mapping of cognitive functions, but do not allow for whole-brain mapping of direct stimulation effects yet^26,27^.

To address these issues, we employed state-of-the-science interleaved TMS-fMRI, the direct recording of brain activity while stimulating a specific cortical area. This approach provides insight into the acute brain dynamics that are associated with the direct interference with cognitive tasks and flexible network adaptation reflecting putative compensatory effects^28–30^. Previous interleaved TMS-fMRI studies in other cognitive domains suggested that TMS induces distributed modulation of brain activity^31–33^. Activity changes within the targeted network were associated with performance disruption^34^, while changes in remote, connected areas or networks may be associated with compensation or improvement (see ref. ^28^).

To probe immediate flexible adaptation during lexico-semantic processing, we targeted the left pMTG (lpMTG) with interleaved TMS-fMRI during a lexico-semantic matching task. Based on the evidence discussed above, we hypothesized that TMS should induce distributed activity increases in the lexico-semantic network and in domain-general control areas. Probing the behavioral relevance of such effects, we expected that local stimulation effects should be associated with performance impairment, while remote changes in domain-general areas should reflect compensatory recruitment and thus be associated with less performance disruption.

We show that acute lexico-semantic disruption triggers rapid, flexible recruitment of domain-general control hubs which likely helps to maintain task performance, thereby elucidating the brain’s capacity for dynamic adaptation to interference and advancing our understanding of putative network-level compensation in lexico-semantic cognition.

## Results

**Fig. 1:**
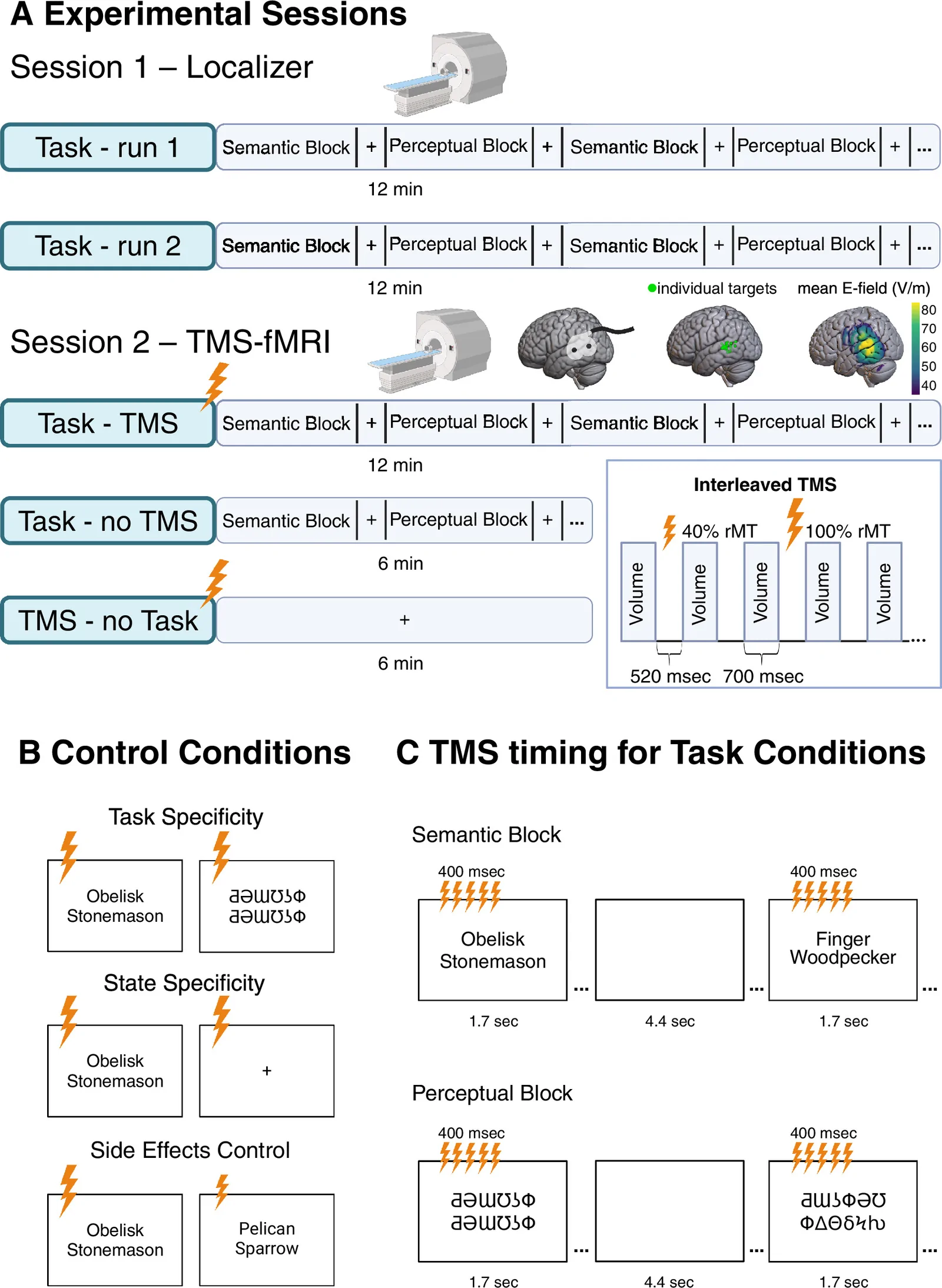
Experimental design and timing of TMS-fMRI. **A** Experimental Sessions. During the localizer session, participants performed two runs of the lexico-semantic matching paradigm, and a T1-weighted anatomical sequence was acquired. For the interleaved TMS-fMRI session, participants performed one run of the lexico-semantic paradigm with individualized difficulty levels during TMS, one run without TMS (“no TMS” baseline with individualized difficulty levels) and one run with TMS at rest (no Task condition). Word pairs (“Semantic Block”) and pairs of perceptual strings (“Perceptual Block”) were presented in alternating blocks. Individual stimulation targets clustered around the left superior temporal sulcus (lSTS; peaks in the superior temporal gyrus (STG) and posterior middle temporal gyrus (pMTG)) with strongest mean electric field (E-field) magnitudes at the gyral crowns of the STG and pMTG. Stimulation was interleaved within volume acquisition gaps of 520 ms and either performed at 40% (low) or 100% (high) of resting motor threshold (rMT) intensity per task block (gray box). **B** Control Conditions. Task Specificity. Stimulation during a control task at the same intensity (high stimulation intensity: 100% rMT). State Specificity. Stimulation during a control state (rest) at high intensity (100% rMT). Side Effects Control. Control for general auditory and somatosensory effects at 100% rMT (high) vs. 40% rMT (low) stimulation intensity. **C** Timing of TMS and task conditions. Each item was presented for 1.7 s with the onset of the TMS burst after 200 ms and a stimulation duration of 400 ms (5 pulses at a frequency of 10 Hertz (Hz)). Stimulation was performed during each trial of the task run. The interstimulus interval (ISI) was 4.4 s. Created in BioRender. Schuler, A. (2026) https://BioRender.com/wpcys1q. Brain visualization for coil position and individual targets was performed using MRIcroGL, brain visualization for E-field using Surf Ice. min: minutes, msec: milliseconds, sec: seconds.

To map the impact of neurostimulation on the semantic network, we employed a lexico-semantic matching paradigm in 25 healthy young participants. First, a localizer session was performed to individually adjust task difficulty and identify individual stimulation targets in the left posterior middle temporal gyrus (lpMTG). Thereafter, participants underwent an interleaved TMS-fMRI session (Fig. 1). During this session, participants performed one run of the lexico-semantic paradigm with either high stimulation intensity (100% of resting motor threshold as effective stimulation, hereafter “high”) or low stimulation intensity (40% of resting motor threshold as control intensity, hereafter “low”) being applied trial-wise, one run of the task without TMS (“no TMS” baseline) and one run of TMS without task (no Task; see “Methods” for further details). For improved readability throughout the Results section, we abbreviate the lexico-semantic task condition as “semantic” in all contrasts, Figures and Tables. This study design allowed controlling for task, state and general side effects in the challenging TMS-fMRI environment (cf. Fig. 1B and “Methods” section for details). fMRI and behavioral results for the “no TMS” baseline condition are reported in Supplementary Fig. 2. In brief, the baseline condition resulted in significant activity increases in core areas of the semantic control and overlapping language network, including left inferior frontal gyrus (IFG) and pMTG.

### TMS induces task-specific activity increases in semantic and domain-general areas during lexico-semantic processing

**Fig. 2:**
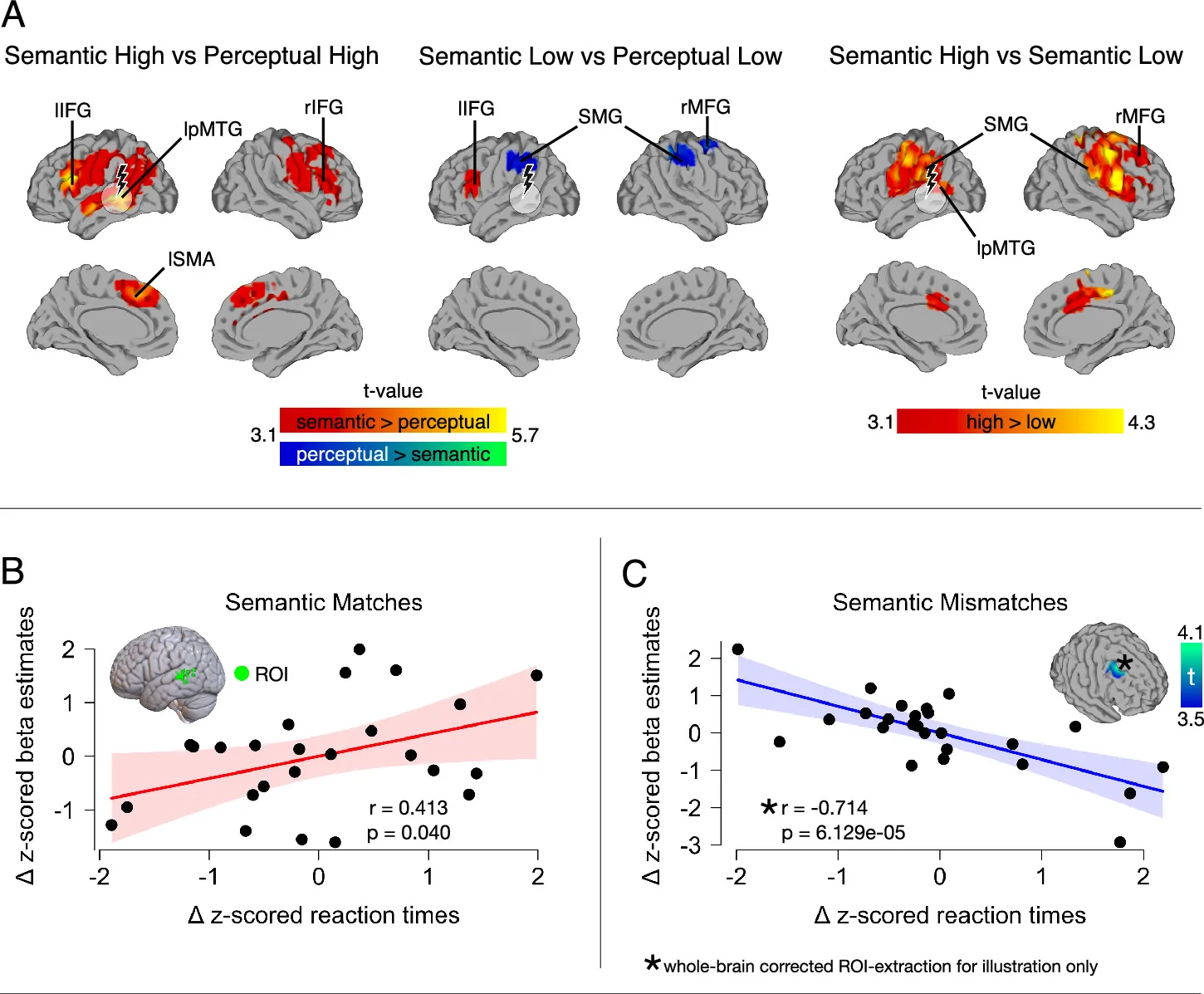
TMS-induced activity changes and behavioral relevance in semantic and domain-general control areas. **A** Significant TMS effects on task-related activity. At high stimulation intensity, TMS-induced increased activity in the semantic control network (lIFG, rIFG, lpMTG, SMA) and beyond contrasting lexico-semantic and perceptual decisions. Comparisons for low stimulation intensity revealed increased activity in the lIFG for semantic > perceptual decisions and increased activity in domain-general control areas (bilateral SMG, rMFG) for perceptual > semantic decisions. Specific intensity effects were reflected in increased activity for high > low stimulation intensity in both semantic (lpMTG) and domain-general control areas (bilateral SMG, rMFG) for lexico-semantic decisions only. The stimulation site is highlighted with a circle and a flash. **B** Relationship between activity increases and stimulation-induced reaction time delays at the stimulation site. Region-of-interest (ROI) analysis, quantified as the difference (Δ) between high and low stimulation intensity during semantic matches. Pearson correlation with bootstrap confidence interval (C.I.), one-sided *p*-value, uncorrected for multiple comparisons. Positive correlation between beta estimates and reaction times. Green dots represent ROI where individual beta estimates were extracted. Each green dot represents one ROI per participant. **C** Relationship between activity increases and stimulation-induced reaction time modulation at the whole-brain level. Stronger stimulation-induced activity increases in the rMFG were associated with less stimulation-induced reaction time delays (whole-brain analysis), quantified as the difference (Δ) between high and low stimulation intensity during semantic mismatches. Scatter plot and regression line are depicted for illustrative purposes. For scatter plots (**B**, **C**), each black dot represents one participant (*n* = 25). Solid lines show fitted ordinary-least-squares regression lines. Shaded bands represent pointwise 95% C.I. Activity maps (**A**, **C**) are thresholded at *p* < 0.001, uncorrected with cluster-level family-wise error (FWE) correction at *p* < 0.05. Source data for plots are provided as Source Data files. Brain visualization for (**A**, **C**) was performed using Surf Ice, brain visualization for **B** using MRIcroGL. lIFG: left inferior frontal gyrus, rIFG: right inferior frontal gyrus, lpMTG: left posterior middle temporal gyrus, lSMA: left supplementary motor area, SMG: supramarginal gyrus, rMFG: right middle frontal gyrus.

We first compared the task-conditions of interest against each other (semantic high, semantic low, perceptual high, perceptual low stimulation intensity) to elucidate task-specific stimulation-induced activity changes (Table 1 and Fig. 2A). Effective high-intensity stimulation of the left posterior middle temporal gyrus (lpMTG) led to task-specific activity increases when contrasting semantic and perceptual decisions. Significant changes were observed in core areas of the semantic control network (SCN), including the stimulation site (lpMTG, left inferior frontal gyrus (lIFG), right inferior frontal gyrus (rIFG), supplementary motor area (SMA)) and in non-semantic somatosensory areas as well as domain-general control regions. Given that the rIFG and SMA are functionally proximal to domain-general control regions, we conducted network-restricted, participant-specific functional region-of-interest (fROI) analyses (see Supplementary Methods, Supplementary Fig. 8). These showed a predominant contribution of the multiple demand network (MDN) to remote stimulation effects in the SMA, alongside smaller but significant SCN engagement. In contrast, the rIFG exhibited stronger SCN engagement. Comparing lexico-semantic and perceptual decisions under low-intensity stimulation selectively revealed increased activity in the lIFG, a core area of the SCN. The opposite contrast (perceptual vs. semantic decisions under low stimulation intensity) resulted in activity increases in perceptual control regions (bilateral supramarginal gyrus (SMG), right middle frontal gyrus (rMFG)). Quantifying the impact of stimulation intensity on lexico-semantic decisions (the direct comparison of high and low stimulation intensity during lexico-semantic processing) revealed activity increases in both areas of the semantic network, including the stimulation site (lpMTG), and domain-general control areas (bilateral SMG, rMFG). Contrasting both stimulation intensities during perceptual decisions did not reveal any significant activity differences, further supporting the specificity of the observed effects. Note that the impact of TMS on task-related activity was not confounded by stimulation-induced changes at the behavioral level (Supplementary Methods and Results (Equivalence Tests) and Supplementary Fig. 3).

**Table 1:** Task-specific TMS effects during lpMTG stimulation

Region	*k*	Side	Cluster Peak (MNI: x, y, z)	*t*-value (peak-level)	*p*-value (cluster-level FWE corrected)
Semantic High > Perceptual High Stimulation Intensity					
Cluster 1	2854				
posterior middle temporal gyrus/superior temporal sulcus/angular gyrus/supramarginal gyrus		left	−56, −36, 4	8.79	<0.001
anterior temporal lobe		left	−52, −6, −10	5.85	<0.001
Cluster 2	2851				
somatosensory cortex/right motor cortex/right inferior frontal gyrus/right middle frontal gyrus		right	16, −4, 38	4.94	<0.001
Cluster 3	1167				
inferior frontal gyrus		left	−48, 18, 24	8.33	<0.001
Cluster 4	380				
somatosensory cortex		left	−58, −12, 40	3.77	0.001
Cluster 5	287				
supplementary motor area		left	−6, 24, 42	5.58	0.004
Semantic Low > Perceptual Low Stimulation Intensity					
Cluster 1	146				
inferior frontal gyrus		left	−46, 18, 24	4.19	0.043
Perceptual Low > Semantic Low Stimulation Intensity					
Cluster 1	420				
supramarginal gyrus		left	−54, −30, 42	4.36	0.001
Cluster 2	343				
supramarginal gyrus		right	44, −40, 56	4.84	0.002
Cluster 3	269				
frontal eye field		right	28, 0, 60	4.82	0.005
Semantic High > Semantic Low Stimulation Intensity					
Cluster 1	5728				
somatosensory cortex/right motor cortex		right	40, −16, 42	6.92	<0.001
supramarginal gyrus		right	50, −14, 46	6.11	<0.001
Cluster 2	4068				
somatosensory cortex/left motor cortex		left	−40, −18, 44	6.11	<0.001
middle temporal gyrus		left	−32, −46, 10	5.29	<0.001
supramarginal gyrus		left	−62, −32, 26	5.06	<0.001
Cluster 3	187				
middle frontal gyrus		right	28, 30, 44	4.03	0.02

Comparisons represent linear contrasts of interest between conditions (semantic high, semantic low, perceptual high, perceptual low stimulation intensity). Statistical thresholds were set to *p* < 0.05, cluster level FWE-corrected, with an initial cluster-defining threshold of *p* < 0.001, uncorrected.

*k* cluster size, *MNI* Montreal Neurological Institute, *FWE* family-wise error.

### Local stimulation effects are associated with delayed semantic reaction times while remote effects reflect potential compensatory activity increases

Notably, we observed activity increases in response to TMS, both in semantic control regions and remote domain-general areas. To elucidate the differential relevance for task performance, we correlated lexico-semantic reaction times for correct responses under TMS (high-low intensity) separately for semantic matches and mismatches. This was a planned decomposition, since behavioral piloting data hinted towards qualitatively different processing strategies for both trial types (Supplementary Methods (Pilot Experiments)). We found a positive correlation between stimulation-induced increases in reaction times and increases in activity at the stimulation site during semantic match trials (*r*(23) = 0.413, *p* = 0.040, 95% bootstrap C.I. [0.085 0.670]; Fig. 2B). In other words, stronger local stimulation effects on task-specific activity are associated with delayed semantic reaction times under TMS. Correlations for semantic mismatches did not reveal any significant effects (*p* > 0.05).

In contrast, we observed a different pattern for remote effects. A whole-brain cluster-level family-wise error (FWE)-corrected regression analysis revealed that stronger upregulation of the domain-general rMFG was associated with less stimulation-induced reaction time delays, potentially reflecting compensatory increases outside the core lexico-semantic network (x, y, z = 36, 16, 54; *t*(23) = 4.62, *p* = 0.038; cluster-level FWE-corrected, Fig. 2C) during semantic mismatch trials. No voxels survived cluster-level FWE-correction for the semantic matches regression analysis.

Electric field (E-field) simulations confirmed that we effectively stimulated the target area (cf. Fig. 1). Accordingly, mean E-field magnitudes were strongest at the gyral crowns of the lpMTG and left superior temporal gyrus (lSTG). Exploratory whole-brain correlations revealed a relationship between delayed lexico-semantic reaction times and E-field magnitudes in the more posterior part of the lpMTG for semantic mismatch trials (Supplementary Fig. 4). Further details and additional analyses are reported in the Supplement (Supplementary Figs. 5–10).

### Task-based connectivity reflects both potential local disruption effects and remote compensatory changes

**Fig. 3:**
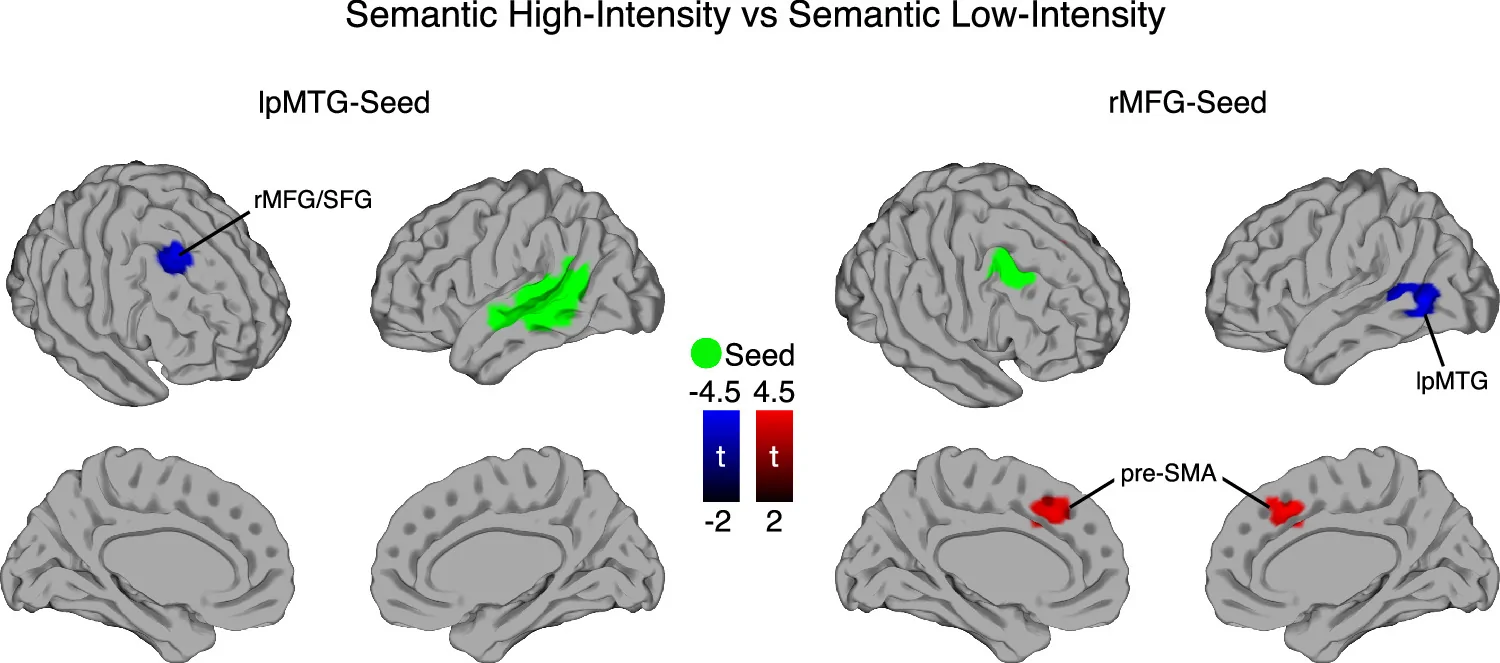
TMS-induced connectivity changes during lexico-semantic processing. Left Panel. Seed-based connectivity between the lpMTG and the rest of the brain showed significant connectivity decreases between the lpMTG and rMFG/SFG. Right Panel. Seed-based connectivity between the rMFG and the rest of the brain revealed significant connectivity decreases between the rMFG and the lpMTG and increases between the rMFG and the pre-SMA. Blue t-values: negative connectivity, red t-values: positive connectivity. Green clusters represent the seed-area. Thresholded at *p* < 0.001, uncorrected with cluster-level family-wise error (FWE) correction at *p* < 0.05. Brain visualization was performed using Surf Ice. lpMTG: left posterior middle temporal gyrus, rMFG: right middle frontal gyrus, SFG: superior frontal gyrus, pre-SMA: pre-supplementary motor area.

For an in-depth investigation of the potential compensatory dynamics between semantic and domain-general regions, we performed task-based connectivity analyses (gPPI) between the targeted lpMTG as well as potential compensatory rMFG and all other areas in the brain, respectively. Comparing connectivity changes between the semantic high-intensity and low-intensity stimulation conditions, we observed decreases in the interaction between the lpMTG (seed region) and rMFG/SFG (x, y, z = 30, 22, 58; *t*(24) > 3.75, *p* = 0.034; Fig. 3, left panel) and between the rMFG (seed region) and the lpMTG (x, y, z = −52, −58, 2; *t*(24) > 3.75, *p* < 0.001; Fig. 3, right panel). In contrast, coupling between the rMFG and the pre-SMA—an intermediate zone between semantic control and multiple demand regions—increased (x, y, z = 0, 20, 40; *t*(24) > 3.75, *p* = 0.028; Fig. 3, right panel).

### Putative compensatory activity increases during semantic processing are state-specific

**Fig. 4:**
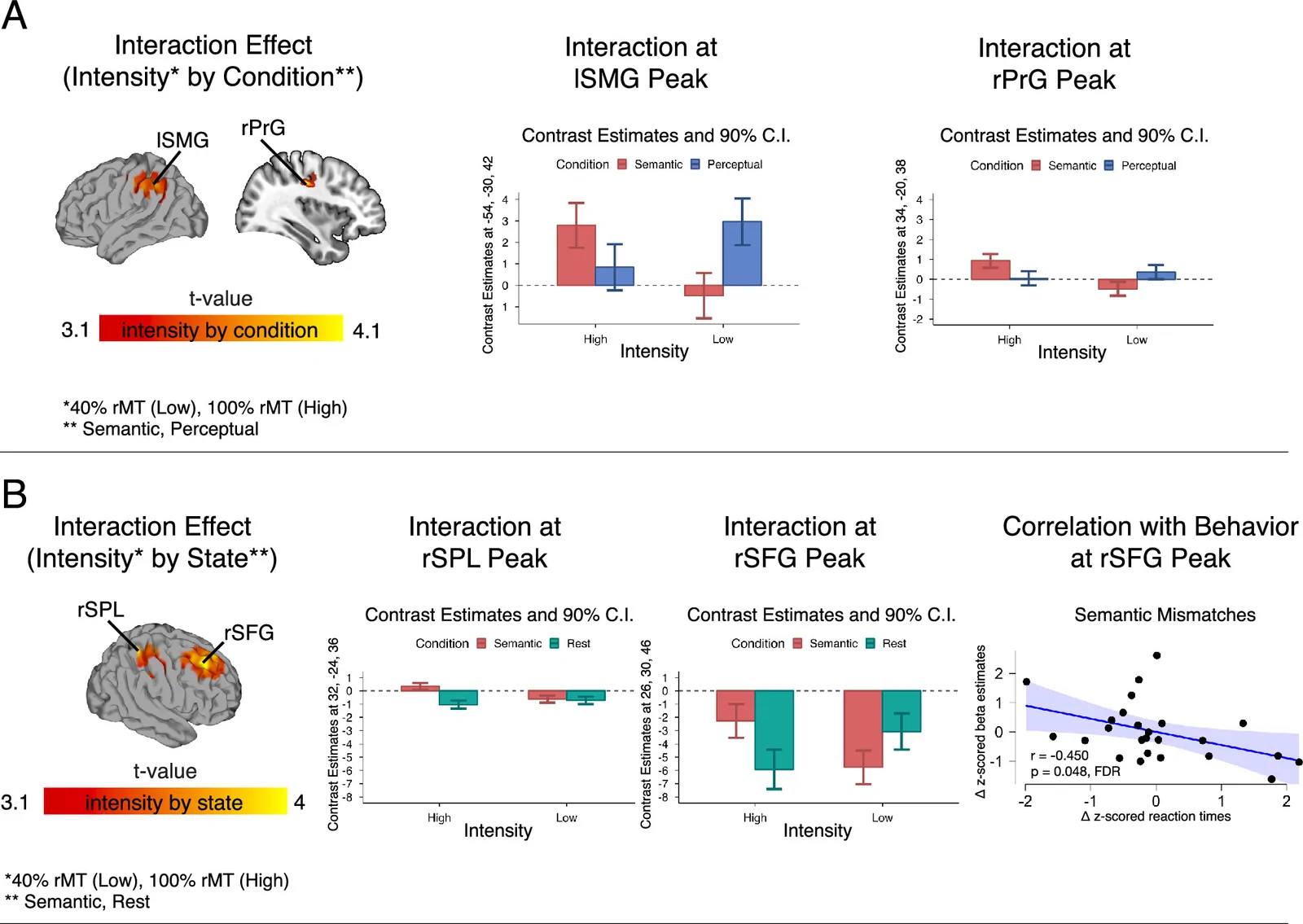
Intensity-by-task and intensity-by-state interactions in frontal and parietal regions with corresponding behavioral correlation. **A** Intensity-by-task interaction. Significant interactions were observed in the left supramarginal gyrus (SMG) and right precentral gyrus (rPrG). These interaction effects were driven by increased activity in the semantic high stimulation intensity and perceptual low stimulation intensity conditions. **B** Intensity-by-brain-state interaction. Significant interactions between brain state (semantic task, rest) and stimulation intensity (high, low) were observed in the right superior parietal lobule (rSPL) and right superior frontal gyrus (rSFG). The latter interaction effect was driven by increased activity in the semantic high-intensity and rest low-intensity conditions, while the former effect was driven by an exclusive activity increase during the semantic high-intensity condition. A negative correlation between stimulation-induced activity increases and reaction times suggests potential compensatory recruitment of the rSFG for trials with semantic mismatches (quantified as difference (Δ) between high and low stimulation intensity). Each black dot represents one participant (*n* = 25). Solid lines show fitted ordinary-least-squares regression lines. Shaded bands represent pointwise 95% confidence intervals (C.I.). Pearson correlation with bootstrap C.I., one-sided *p*-value, FDR-corrected for *n* = 2 comparisons. Contrast estimates (bar graphs **A**, **B**) are represented as means with 90% confidence interval (C.I.; error bars) for *n* = 25 participants. Bar graphs are depicted for illustrative purposes. No statistical test was applied. Activity maps (**A**, **B**) are thresholded at *p* < 0.001, uncorrected with cluster-level family-wise error (FWE) correction at *p* < 0.05. Brain visualization was performed using Surf Ice and MRIcroGL. Source data for plots are provided as Source Data files.

Next, we further elucidated task-specificity by modeling interaction effects between task condition (semantic, perceptual) and stimulation intensity (high, low; Fig. 4A).

We found significant interactions in the left supramarginal gyrus (lSMG) and right precentral gyrus (rPrG). These effects were driven by significant activity increases for the semantic high and perceptual low stimulation intensity conditions in the lSMG (x, y, z = −54, −30, 42; *t*(96) = 4.69, *p* < 0.001) and in the rPrG (x, y, z = 34, −20, 38; *t*(96) = 4.55, *p* = 0.008). The activity increase for the semantic condition in the lSMG may indicate stronger involvement of a neighboring domain-general area to compensate for acute perturbation in a semantic area, while the contribution of this area in the low-intensity control condition for the perceptual task might be explained by peripheral TMS effects that interact more strongly with neural processing of attention. The stronger stimulation-induced engagement of right motor areas might reflect effortful response execution. Stimulation-induced increases in rPrG were associated with a trend for less stimulation-induced delays in semantic reaction times during semantic match trials (*r*(23) = −0.414, *p* = 0.080, false discovery rate (FDR)-corrected), but there was no significant correlation for semantic mismatch trials. There was no significant correlation between activity changes in the left SMG and reaction times for any of the conditions.

Finally, we observed brain state-dependent stimulation effects, quantified by the interaction of state (semantic task, rest) and stimulation intensity (high, low; Fig. 4B). Significant interactions were observed in the right superior parietal lobule (rSPL; x, y, z = 32, −24, 36, *t*(96) = 4.45, *p* = 0.002) and right superior frontal gyrus (rSFG; x, y, z = 26, 30, 46, *t*(96) = 4.57, *p* < 0.001). Stimulation-induced activity increases in these domain-general areas for the lexico-semantic task likely reflect stronger recruitment of attentional resources as a putative compensatory mechanism to acute semantic disruption. Indeed, a negative correlation with lexico-semantic reaction times revealed that stimulation-induced increases in activity in the rSFG were associated with less stimulation-induced disruptions in reaction times for semantic mismatches (*r*(23) = −0.450, *p* = 0.048, FDR-corrected, 95% bootstrap C.I. [−0.724 −0.117]). No significant effect was found for semantic matches in the rSFG. Finally, for rSPL, there was a trend towards a negative correlation between semantic reaction times and activity changes for semantic matches (*r*(23) = −0.433, *p* = 0.062, FDR-corrected) but no significant correlation for semantic mismatches. There were no significant interactions in the intensity-by-state model for the perceptual vs. rest conditions. Consistent with models of semantic control emphasizing the flexible engagement of distributed fronto-parietal regions^35,36^, putative compensation effects were thus state-dependent and qualitatively different at rest.

### Putative compensatory recruitment of domain-general frontal control regions during lexico-semantic processing

**Fig. 5:**
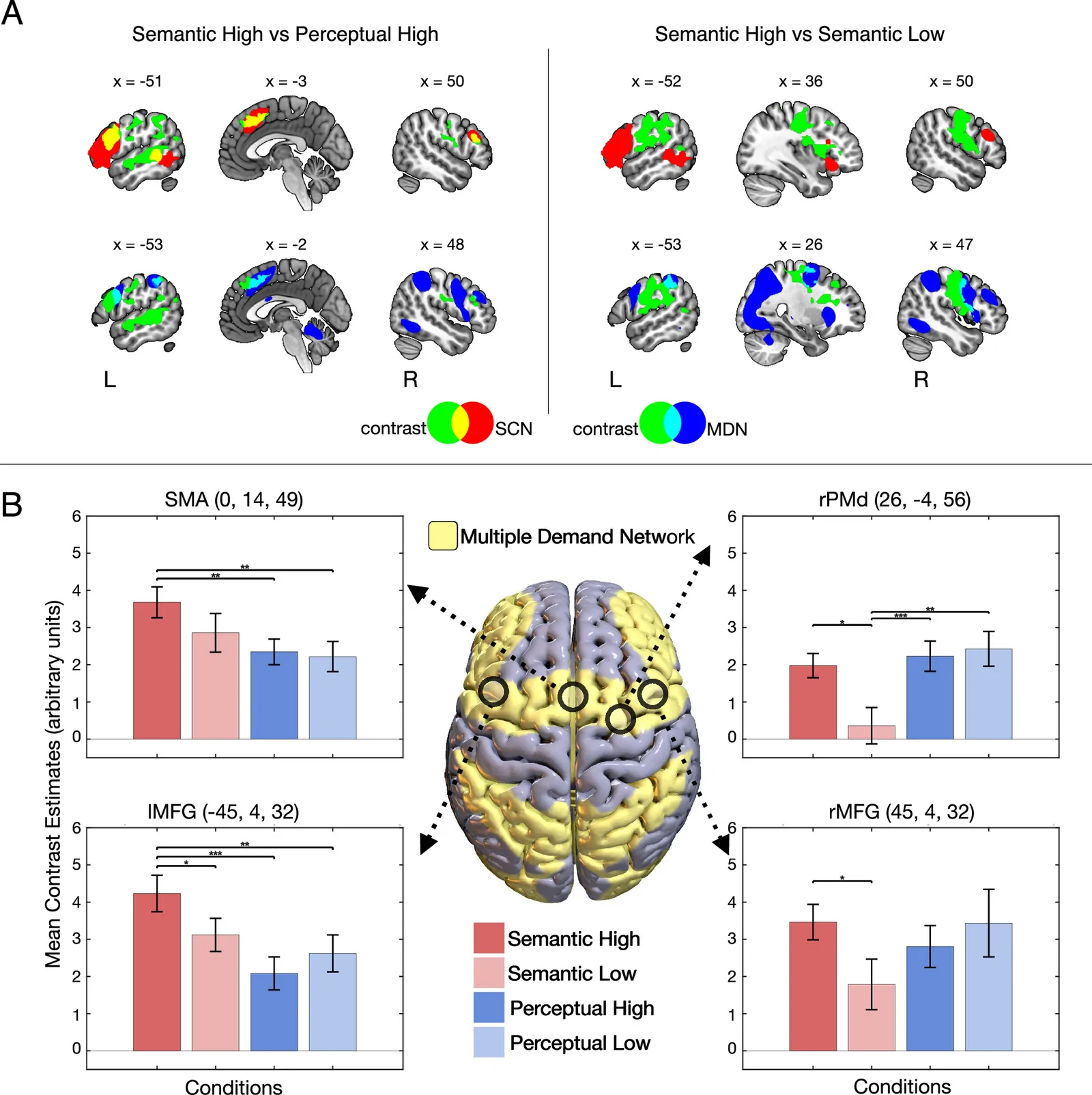
Stimulation-induced semantic activity increases in multiple demand and semantic control regions. **A** Overlap of task contrasts with the multiple demand network (MDN) and semantic control network (SCN). The semantic high vs. perceptual high contrast (green) overlapped with the SCN (red) in the bilateral inferior frontal gyrus (IFG) and left posterior middle temporal gyrus (lpMTG; yellow). Overlap with the MDN (blue) was found in the left middle frontal gyrus (MFG), superior parietal lobule (SPL) and supplementary motor area (SMA; turquoise). The semantic high vs. semantic low contrast (green) overlapped with the MDN (blue) at the left SPL and right MFG (turquoise). MDN map obtained from ref. ^75^, SCN map obtained from ref. ^8^. **B** TMS-induced activity increases in frontal multiple demand network regions. Significant activity increases for the semantic high-intensity stimulation compared to the low-intensity stimulation condition were found in the bilateral middle frontal gyrus (lMFG, rMFG) and right dorsal premotor cortex (rPMd). In the rPMd, activity was significantly lower for the semantic low condition compared to all other conditions. In the SMA, the semantic high stimulation intensity condition led to significant signal increases compared to both perceptual conditions. Pairwise comparisons were performed with one-sided t-tests, *p*-values were Bonferroni-corrected for six comparisons. Bar graphs represent means ± standard error of the mean (SEM) for 25 participants. * *p* < 0.05 ** *p* < 0.01 *** *p* < 0.001 (Bonferroni-corrected). Exact *p*-values are provided in Supplementary Data 1 and the Source Data files. Source data for plots are provided as Source Data files. Brain visualization for **A** was performed using MRIcroGL.

Finally, we created overlap maps between our main contrasts (semantic high vs. perceptual high, semantic high vs. semantic low) and the semantic control and multiple demand networks (SCN, MDN; Fig. 5A). The semantic high vs. perceptual high contrast measured the TMS effect on lexico-semantic processing, while controlling for peripheral effects of high-intensity stimulation, symbol processing, and decision making. This contrast overlapped with the SCN at the bilateral IFG, SMA and lpMTG, and with the MDN at the left MFG, SPL and SMA. Furthermore, there was overlap with the MDN for the semantic high vs. semantic low contrast in the rMFG and lSPL. The latter contrast measured the TMS effect during lexico-semantic processing while partly controlling for peripheral effects and the direct effect of lexico-semantic decisions.

To further elucidate putative compensatory activity shifts towards domain-general control regions during lexico-semantic perturbation in the lpMTG, we performed exploratory participant-independent region-of-interest (ROI) analyses in the multiple demand network (MDN; Fig. 5B).

These region-of-interest analyses revealed the following findings (see Supplementary Data 1 for details): 1) stronger recruitment of bilateral MFG and right dorsal premotor cortex (rPMd) for the semantic high stimulation compared to the semantic low stimulation intensity condition, 2) overall stronger recruitment of lMFG for the semantic high stimulation intensity condition compared to all other conditions, 3) less engagement for the semantic low stimulation intensity condition relative to all other conditions in the rPMd, and 4) stronger recruitment of the SMA for the semantic high stimulation intensity condition compared to both perceptual conditions. Consistent with its putative role as an interface between semantic and domain-general control, complementary subject-specific fROI analyses within SCN and MDN masks confirmed this pattern for the SMA, with a relatively stronger engagement of the MDN. Similarly, functionally restricted ROI analyses for the lMFG converged with participant-independent results (compare Fig. 5B and Supplementary Fig. 8). Collectively, these results suggest that perturbation of the left posterior middle temporal cortex during lexico-semantic processing resulted in immediate activity increases in domain-general frontal areas. Note that activity increases in the MDN under effective high-intensity stimulation were only observed for the semantic condition, highlighting the specificity of our effect.

We did not find any significant differences in stimulation-induced activity changes for the remaining ROIs within the MDN after corrections for multiple comparisons.

## Discussion

**Fig. 6:**
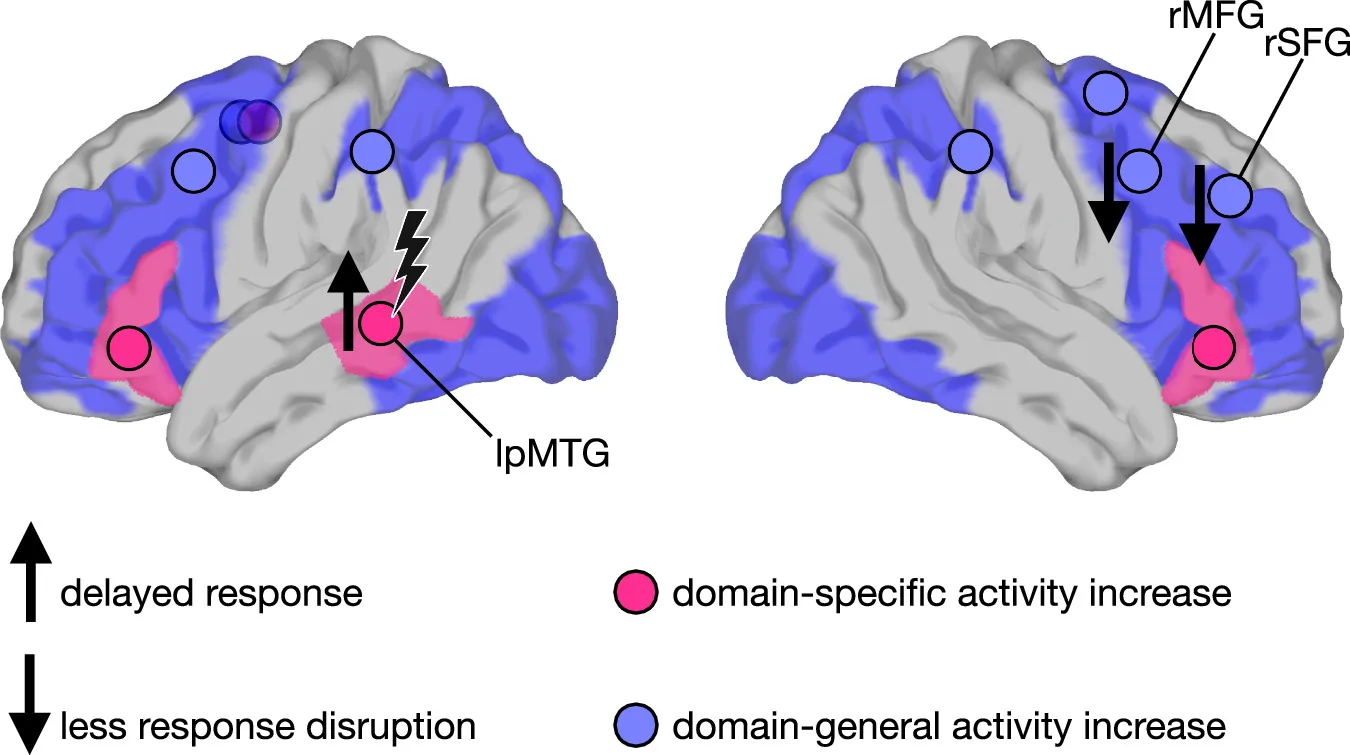
Perturbation of lpMTG triggers acute semantic network responses and domain-general activity shifts associated with preserved performance. Summary of the main findings. Visualization of immediate activity changes in domain-specific semantic areas (pink circles) and domain-general regions (purple circles). Local activity increases in the left posterior middle temporal gyrus (lpMTG) were associated with delayed lexico-semantic reaction times, while activity increases in remote domain-general areas were correlated with less disruption in lexico-semantic reaction times. Purple mask: outline of the domain-general multiple demand network. Pink mask: areas of the lexico-semantic network. Brain visualization was performed using Surf Ice. lpMTG: left posterior middle temporal gyrus, rMFG: right middle frontal gyrus, rSFG: right superior frontal gyrus.

In this study, we demonstrate that acute perturbation of the left posterior middle temporal gyrus (lpMTG) with high-intensity TMS induces immediate, widespread activity increases in semantic and domain-general control regions that differentially link to behavior. At the target site, we found that stimulation-induced activity increases were correlated with stimulation-induced delays in semantic reaction times, indicating a decrease in response efficiency. In contrast, stimulation-induced activity increases in domain-general (control) areas were negatively linked with behavioral stimulation effects, likely reflecting less disruption of performance under TMS and putative compensatory attempts of the brain to maintain performance. Collectively, these results provide initial insight into the brain’s potential for rapid adaptation in response to acute perturbation during lexico-semantic processing. Specifically, our results support a potential beneficial contribution of domain-general regions under challenging conditions, when domain-specific lexico-semantic processing is perturbed. These effects were task-specific. Additional state-specificity was demonstrated as putative compensatory activity shifts under effective stimulation specific to lexico-semantic decisions relative to rest. The main results are summarized in Fig. 6.

As a first main finding, effective high‑intensity stimulation of the lpMTG during lexico-semantic processing led to increased activity in both local and remote semantic control regions (bilateral IFG, lpMTG, SMA), relative to the high‑intensity perceptual control condition. Moreover, high-intensity semantic stimulation elicited greater local lpMTG activity increases compared to low-intensity stimulation. Local activity increases during the effective high-intensity semantic match condition were associated with slower reaction times, indicating a within-network perturbation effect, similar to prior concurrent TMS-fMRI findings in the attention domain^34^. Moreover, lexico-semantic reaction time delays were stronger with increased electric field strengths in the more posterior portion of the lpMTG for semantic mismatches, supporting local TMS effects on semantic control as reported previously^1,13,37^.

As a second main finding, effective high-intensity stimulation during lexico-semantic processing not only increased local lpMTG and within-network semantic control activity but also upregulated rMFG activity compared to low-intensity stimulation. Complementary whole-brain regression analysis indicated that rMFG upregulation may serve a compensatory role during acute semantic interference, as shown by reduced TMS-induced behavioral disruption during semantic mismatches. These findings converge with prior concurrent TMS-fMRI studies showing that remote activity increases are associated with performance gains in semantic memory tasks (e.g., refs. ^31,38,39^).

As hypothesized, effective pMTG stimulation during lexico-semantic processing elicited further widespread activity changes beyond the targeted semantic control network (SCN), relative to the perceptual control condition. Activity increases were observed in the bilateral somatosensory cortices, right frontal lobe and extended into the left anterior temporal lobe and parietal cortex as general semantic regions. These activity increases were partially overlapping with the contrast between semantic effective high-intensity and low-intensity conditions (activity increases in the somatosensory and motor cortices, and bilateral SMG). Together, these results suggest potential compensatory upregulation of domain-general and domain-specific areas as a direct response to perturbing a domain-specific semantic control region, consistent with the literature^14,40,41^.

Complementary whole-brain connectivity analyses showed disrupted task-related connectivity between the lpMTG and rMFG/SFG for semantic high- vs. low-intensity conditions. This decrease in connectivity under effective stimulation may indicate less efficient coordination of control resources and likely contributed to delayed performance on demanding (high-intensity) semantic trials. By contrast, upregulation of an adjacent rMFG subregion and its increased functional coupling with the pre-SMA—a hub at the interface of semantic and domain‑general control—likely reflects network modulation consistent with potential compensation following transient disruption of semantic processing^6^.

In contrast to effective high-intensity stimulation, low-intensity stimulation elicited stronger activity changes during the perceptual control condition, potentially recruiting the contralateral (left) SMG beyond right SMG and PMd. This may reflect either sensory interaction effects between peripheral stimulation and attention^42,43^ or compensatory neural responses to peripheral distraction^44,45^.

Furthermore, the task-condition-by-intensity interaction revealed increased left SMG activity during semantic high- and perceptual low-intensity conditions. This might reflect recruitment of a domain-general area to compensate for pMTG interference (semantic high-intensity condition) and increased attentional processing as a response to sensory TMS effects (perceptual low-intensity condition;^46,47^). Finally, a selective activity increase in the right precentral sulcus for lexico-semantic processing suggests additional involvement of more unspecific neural responses to interference with pMTG^48^.

Beyond the comparison between the perceptual and lexico-semantic tasks, we observed significant state-dependent interaction effects for semantic vs. rest conditions in the right SPL and SFG. While the effect in the rSPL reflected increased activity solely for high-intensity stimulation during the lexico-semantic task, the effect in the rSFG was driven by decreased deactivation during the same condition and decreased deactivation during rest for low-intensity stimulation. At the behavioral level, activity increase in the rSFG correlated with less disruption of lexico-semantic reaction times. These results further underscore the role of domain-general cognitive control areas in sustaining domain-specific functions under acute interference^49^.

Collectively, our findings highlight state-dependent interaction effects of online TMS^50,51^: stimulation during lexico-semantic processing relative to a perceptual control task revealed task-specific activity increases in the lSMG, whereas the comparison of the lexico-semantic task state with rest indicated a shift toward right-hemisphere regions associated with cognitive control. The latter effect is consistent with increased recruitment of right-hemispheric fronto-parietal regions in semantically challenging situations^35,36^. These observations provide insights into both the mechanisms of TMS and the dynamic allocation of cognitive resources. Combining different control conditions (task-, state-specificity and general side effects control), we were able to dissociate unspecific side effects evoked by arousal, startle responses, or peripheral effects from compensatory mechanisms in response to TMS.

Region-of-interest (ROI) analyses within the multiple demand network (MDN) revealed that high‑intensity TMS during lexico-semantic decisions increased activity in bilateral MFG and SMA, consistent with enhanced domain-general control under elevated semantic demands. The lMFG exhibited increased activity for high vs. low stimulation intensity during lexico-semantic compared to perceptual decisions, suggesting a left-lateralized bias for semantic control demands.

In contrast, the right MFG and PMd showed increased activity during high compared with low stimulation intensity in the lexico-semantic task, but comparable activity across both stimulation intensities in the perceptual task (no significant intensity effect), consistent with their role in domain-general difficulty and performance monitoring^52^. The SMA showed the strongest response for high stimulation intensity during lexico-semantic trials, indicating increased demands on response selection and temporal coordination under lexico-semantic perturbation.

Together, these findings suggest that high-intensity TMS transiently elevates semantic control demands, supported by flexible recruitment of MDN regions with partially dissociable domain specificity, in line with models of resilient semantic cognition involving interactions between domain-general and semantic-specific control systems (e.g., refs. ^41,49^).

Strikingly, brain-behavior correlations between increased activity and relatively preserved performance occurred only in right frontal areas for semantic mismatches, while less disruption for semantic matches was associated with a trend for right-hemispheric involvement of motor and posterior attention regions. In contrast, stronger lexico-semantic disruption was associated with local activity increases in the stimulated temporal lobe for semantic matches. This might be explained by different processing strategies for semantic mismatch conditions that require the inhibition of linking concepts rather than actively deciding on a connection between two concepts. These trials require more divergent lexico-semantic search and the ability to inhibit loose associations between presented concepts and thus trigger stronger engagement of frontal resources^37,53^.

In contrast, increased activity around the stimulation site paired with less efficient responses for semantic match trials might reflect a failed attempt to compensate for interference with more related, but still demanding, semantic concepts. Given that task difficulty was individually adjusted to participants’ performance levels, we expected that semantic matches would be processed via the more effortful semantic control system rather than the more automatic parietal pathway^37,52,54,55^. Increased lpMTG activity in slower participants might reflect initial upregulation due to higher task demands, which interacted more strongly with the concurrent stimulation. Moreover, a trend towards stimulation-induced activity increases in rPrG being associated with less response disruption during semantic match trials suggests less automated processing and increased reliance on semantic control^56^. Importantly, BOLD-fMRI signals are insensitive to whether the underlying neural activity is of excitatory or inhibitory nature. Consequently, behavioral outcomes serve as a proxy for interpreting the direction of these effects.

Collectively, our findings reveal insights into the acute neural and behavioral processes associated with adaptation triggered by interfering with a key semantic control region in the lpMTG. These findings support and extend previous results on short-term plasticity induced by perturbing the semantic system with offline TMS (e.g., refs. ^46,57^). While the previous findings were interpreted as short-term reorganization within or across networks^21^, our present findings demonstrate that the latter mechanism also occurs as an immediate response to perturbation, likely reflecting a shift in the balance of the relative contribution of different networks in the brain to maintain efficient task performance. Importantly, we found correlations between task-specific activity increases and efficient behavior for the semantic mismatch condition in right frontal domain-general control areas. Corroborating this pattern, activity in frontal MDN areas was upregulated as a response to effective stimulation during lexico-semantic processing. Collectively, these results provide initial evidence consistent with a putative compensatory role of frontal regions to prevent performance disruption, especially in the presence of more divergent semantic concepts that require active inhibition.

Aside from providing insight into flexible adaptation in the semantic network, our findings demonstrate how combining TMS with fMRI enables a shift from static localization of cognitive functions (“where”) toward a mechanistic account of network dynamics (“how”) underlying semantic processing. Consistent with emerging non-modular frameworks^58^, this approach provides initial evidence for distributed and interactive lexico-semantic processing. By transiently perturbing a targeted node while measuring whole‑brain activity, interleaved TMS-fMRI reveals how neural activity is redistributed across the semantic and support networks, identifying regions that exhibit reduced engagement, relative stability or putative compensatory upregulation. More broadly, integrating interleaved TMS-fMRI with emerging approaches such as multi-channel TMS^59^ in future studies holds promise for advancing a more fine-grained understanding of large-scale neural dynamics in lexico-semantic cognition.

In summary, our findings provide insight into how semantic and domain-general regions adjust their balance to support efficient lexico-semantic processing. Future studies could investigate these mechanisms further to determine whether targeted non-invasive brain stimulation of domain-general areas may represent a potential avenue for supporting recovery in patients with impaired lexico-semantic processing after brain lesions. In particular, frontal nodes of the MDN may represent promising stimulation targets following damage to lexico-semantic brain regions, although this possibility requires further investigation.

### Limitations

Our study provides insight into the acute adaptation processes in response to perturbations of lexico-semantic processing, although certain considerations should be taken into account.

First, despite being an established control condition in interleaved TMS-fMRI research^28,30^, the low-intensity stimulation condition cannot fully separate auditory/somatosensory effects from intensity-dependent neural effects.

Second, reaction times were generally faster in the perceptual condition, suggesting that domain-general activity changes for the semantic high vs. perceptual high contrast might be partly driven by differences in task difficulty.

Third, although we included a relatively large sample compared to typical interleaved TMS-fMRI studies of cognition (cf.^28^), the observed brain-behavior correlations should be interpreted as converging rather than definitive mechanistic evidence for compensatory processes. Future studies should further examine the functional relevance of putative compensatory upregulation.

Finally, although stimulation was delivered at 200 ms post word-onset—after the earliest stages of visual and orthographic word-form encoding are largely resolved—and although the pMTG is not typically implicated in low-level perceptual or phonological input processing, we cannot fully exclude a contribution of upstream lexical retrieval processes that may still be ongoing at this latency. Our task-based difficulty manipulation, which specifically targeted semantic selection demands, supports an interpretation centered on (lexico-)semantic control rather than purely pre-semantic processing; however, we acknowledge that lexical and semantic retrieval processes are not fully dissociable in the present paradigm, and this should be considered when generalizing our conclusions.

## Methods

### Participants

Twenty-five healthy right-handed volunteers (mean age = 31.04 ± 5.98 (standard deviation (SD)), 13 female) participated in the study. Upon registration, participants self-reported their gender by selecting one of three options: “female” (13), “male” (12), or “diverse” (0). The sample was chosen to include a balanced gender distribution (female/male) across young adulthood. Exclusion criteria were contraindications to magnetic resonance imaging (MRI) or TMS, language impairments, proficiency in any language based on the Cyrillic, Greek or Georgian writing system, psychiatric or neurological disorders. All participants gave written informed consent prior to participating in the study. The study followed the recommendations of the Helsinki Declaration (1964) and was approved by the Ethics Committee of the Medical Faculty of Leipzig University. Participants were financially reimbursed for their participation. Participants were recruited via the database of the Max Planck Institute for Human Cognitive and Brain Sciences (MPI CBS). Data were collected between 2023 and 2024.

### Experimental paradigm and procedure

Experimental Paradigm. We used an adapted version of a lexico-semantic matching task (ref. ^55^; Psychtoolbox 3.0.19). In this task, word pairs have to be categorized as semantically related (matching, e.g., “calendar” and “date”) or semantically unrelated (mismatching, e.g., “pear” and “ring”). The task uses an adaptive staircase procedure to obtain comparable task demands between participants. We chose this paradigm because it was specifically designed to minimize contamination of fMRI measures of lexico-semantic processing by activity changes driven by task difficulty. This allowed us to map TMS-induced increases in the recruitment of domain-general brain areas during lexico-semantic processing. Each word pair is assigned a difficulty level of 1–7. The adaptive staircase employs a 2-down, 1-up methodology where two correct responses in a row upgrade the subjects to more difficult word pairs. An incorrect response shifts the subjects two levels downwards.

Following^55^, a perceptual decision-making condition consisting of pairs of false font strings was used as a non-linguistic control task. Subjects had to categorize false font strings as either perceptually matching (e.g., “ƋƏƜƱʖΦ”, “ƋƏƜƱʖΦ”) or mismatching (e.g., “ƋƏƜƱʖΦ”, “ΦΔΘδϞƕ”). This task controlled for visual, sensorimotor, executive and decision-making processing. Difficulty was manipulated with the similarity of letter strings, with higher visual similarity of letter strings leading to increased difficulty. The original task was modified for application during interleaved TMS-fMRI. Modifications included a reduction of the number of items per block to five and slight changes to the visual task design, as well as implementation as forced-choice task, where participants had to press with the left index finger when the items were matching and with the left middle finger when the items were not matching.

Experimental Procedure. Participants underwent two experimental sessions (Fig. 1A). During the first session, we acquired two runs of the adapted German version of the lexico-semantic matching task as a localizer (see Supplementary Methods for details on item creation). Additionally, we acquired a T1-weighted image for TMS neuronavigation targeting. During the second session, participants underwent one run of the lexico-semantic matching task while either receiving bursts of 5 pulses of 10 Hertz (Hz) TMS at 100% (high-intensity) or 40% (low-intensity) of their respective resting motor thresholds. Items were set to the individualized median difficulty for lexico-semantic and perceptual performance based on the two localizer runs. 8 blocks of each condition (semantic, perceptual) were included, with presentation in alternating order. Low- and high-intensity TMS was applied during 4 blocks, respectively. Stimulation intensity was semirandomized per block. Match and mismatch items were semirandomized across blocks but balanced to include 50% of matches for each intensity and condition. The experimenter was in a separate room from the participant during data acquisition. Stimulation intensity was set automatically by the task. The number of matches and mismatches was balanced per condition to enable decomposition analyses of qualitatively different trial types. Conceptually, semantic match trials primarily engage convergent retrieval and verification of appropriate associations, whereas semantic mismatch trials place greater demands on conflict monitoring, divergent lexico-semantic search, and the inhibition of inappropriate links, thereby recruiting partially distinct control mechanisms and processing strategies^37,53,54^. Items were presented at a fixed interval of 5 repetition times (TRs; 6.1 s) to diminish startle responses or surprise effects from TMS onsets^43,60–62^. Each item was presented for 1.7 s. The interstimulus interval (ISI) was 4.4 s. 10 TRs (12.2 s) with a fixation cross were added between blocks to allow hemodynamics to go back to baseline. In total, this run lasted 12 minutes (min). Next, participants performed a task run with half of the items without stimulation (“no TMS” baseline) for 6 min. Finally, participants underwent a short stimulation run at rest (i.e., without the task while fixating a cross), which was also 6 min long.

### Control conditions

TMS has several side effects, including auditory and somatosensory activity as well as muscle twitches and attentional shifts, that necessitate rigorous control conditions. Addressing these confounds is particularly challenging in TMS-fMRI settings, where MR-compatible sham coils are unavailable and peripheral stimulation can substantially influence the BOLD signal, complicating the dissociation of transcranial and non-transcranial effects. To mitigate these limitations, we implemented a multi-level control framework in line with recent recommendations^28,30^. Specifically, we contrasted (i) a task of interest with a control task (task specificity: semantic vs. perceptual), (ii) task engagement with a rest state (state specificity: semantic vs. rest), and (iii) effective vs. subthreshold stimulation intensities (side effects control: 100% vs. 40% resting motor threshold (rMT)). While the latter controls for auditory and somatosensory confounds, it does not fully dissociate intensity-dependent effects. The integration of these complementary control conditions provides a robust basis for isolating transcranial contributions and offers a refined characterization of TMS-induced effects (see Fig. 1B).

Participant-specific TMS side effects were assessed with the TMSSens_Questionnaire (ref. ^63^; Supplementary Table 2).

### Imaging data acquisition

All data were acquired on a 3-Tesla Siemens Skyra magnetic resonance tomograph (Siemens, Erlangen, Germany). Data from the localizer session were acquired with a standard 32-channel radiofrequency (RF) coil. TMS-fMRI data were acquired with two 7-channel surface RF coils—one with the TMS coil mounted on top and the other positioned over the contralateral hemisphere^64^. For neuronavigation, tissue segmentation of the functional localizer data and electric field modeling, we used a T1-weighted MPRAGE sequence (TR/TI/TE = 2300/900/2.98 ms, flip angle 9°, voxel size 1 mm^3^). For segmentation of TMS-fMRI data, we acquired an MP2RAGE sequence (TR/TI1/TI2/TE = 5000/700/2500/2.85 ms, flip angle 4/5°, voxel size 1 mm^3^). Echo-planar imaging (EPI) was performed with a CMRR sequence (TR/TE = 1220/38 ms, flip angle 45°, voxel size 3 mm^3^, MB-factor = 4, volume acquisition gap = 520 ms;^65^). For an overview on motion and temporal signal-to-noise ratio (tSNR) quality checks, please refer to Supplementary Table 4.

### Transcranial magnetic stimulation (TMS)

TMS was performed using a MagProX100 stimulator and the MR-compatible MRi-B91 TMS coil (MagVenture, Farum, Denmark). Resting motor threshold (rMT) was defined by visual inspection and the help of a Parameter Estimation by Sequential Testing (PEST) procedure employing the Motor Threshold Assessment Tool (MTAT 2.0;^66^, https://www.clinicalresearcher.org/software.htm) while the participant was seated next to the scanner bore and with the 7-channel RF coil attached to the TMS-coil. We chose a stimulation threshold of 100% of the individual rMT because a validated functional threshold for our specific task was not yet available^29^. Our choice was further supported by a previous study demonstrating stronger neural responses to stimulation in association cortex at 100% rMT compared with both lower and higher intensities^67^. During the main experiment, 5 pulses at 10 Hz were applied during each trial 200 ms after visual stimulus onset, either at 100% or 40% of the individual rMT (Fig. 1C). We chose this delay since it guaranteed stimulation during different stages of semantic processing (200–600 ms) without affecting early visual-spatial processing (0–200 ms;^68–70^). Please note that we calibrated the timing for the most effective lexico-semantic disruption in a pilot experiment (see Supplementary Methods (Pilot Experiments)). Bursts were applied during volume gaps of 520 ms (Fig. 1A, gray box).

To target the individually defined area within the lpMTG, we used the semantic > perceptual contrast across two runs from the localizer session. The mean fMRI-derived stimulation target coordinates were x, y, z = −66.40, −39.76, 4.26 (SD: 1.53, 6.60, 4.75; MNI space). Individual fMRI peaks in the lpMTG during lexico-semantic processing were projected to the cortical surface of individual T1 images in the Localite neuronavigation software (Fig. 1A; Localite, Sankt Augustin, Germany), with the coil rotation angle sulcus aligned with the STS^71^. The TMS-coil was then positioned using frameless stereotaxy on the scanner bed and a dedicated coil holder (MagVenture, Farum, Denmark).

To simulate induced electric fields for TMS-fMRI, we used SimNIBS v4.5^72^. We first employed the charm command line tool to create a finite element method (FEM) head-model. Then, we fed the affine transformation matrix for each participant’s final stimulation TMS-coil position into the simulation, considering the corresponding TMS-coil model (MRi-B91;^73^). Supplementary analyses can be found in the Supplementary Methods and Supplementary Fig. 4.

### Statistical analyses

#### Behavioral data

Reaction times (RT) were analyzed using linear mixed-effects regression with the lme4 and lmerTest packages in R (RStudio 2022.07.1). The dependent variable was z-standardized log-transformed RT for correctly solved items. Fixed effects included task, intensity, and match and their interactions. The random-effects structure comprised random intercepts for participants and items. Trial-level accuracy (correct = 1, incorrect = 0) was analyzed using generalized linear mixed-effects models with a binomial error distribution and logit link. Fixed and random effects mirrored the RT model, with task, intensity, and match (and their interactions) as fixed effects, and random intercepts for participants and items.

#### fMRI analyses

Preprocessing included bias correction implemented in ANTs (2.5.0; http://stnava.github.io/ANTs), despiking using AFNI (23.2.19; http://afni.nimh.nih.gov/afni), realignment with SPM12 (r7771; https://www.fil.ion.ucl.ac.uk/spm/software/spm12/), segmentation of anatomical images using CAT12 (http://www.neuro.uni-jena.de/cat/), normalization of EPIs based on deformation fields derived from anatomical normalization and 6 mm spatial smoothing in SPM12.

First- and second-level analyses were performed in SPM12. First-level analyses included modulation of four regressors (semantic high-intensity, semantic low-intensity, perceptual high-intensity and perceptual low-intensity) against the implicit baseline. To explore the impact of differences in task processing for matching vs. mismatching, we set up an additional model that separated matches and mismatches for each condition, thus comprising eight regressors.

First-level estimates were entered into a flexible factorial design to model the main effects of stimulation intensity (high vs. low) and task condition (semantic vs. perceptual), as well as their interaction and pairwise comparisons. For exploratory purposes, an additional second-level fMRI model was estimated that incorporated matches vs. mismatches as a third factor (Supplementary Fig. 9). The “no TMS” run was modeled analogously, including comparisons for task conditions (semantic vs. perceptual). Due to technical issues, the “no TMS” run was missing for one participant. Finally, separate models were specified to examine the interactions between semantic and rest, as well as perceptual and rest conditions, with high and low stimulation intensities modeled for all conditions. Statistical thresholds were set to *p* < 0.05, cluster level FWE-corrected, with an initial cluster-defining threshold of *p* < 0.001, uncorrected. All coordinates are provided in MNI space.

#### Generalized psychophysiological interaction (gPPI)

To elucidate TMS intensity specific connectivity modulations, we used the CONN toolbox (22.v2407). Four task conditions were modeled as miniblocks without hemodynamic response function (HRF) convolution as implemented in the toolbox to account for acquisition gaps (semantic high, semantic low, perceptual high, perceptual low;^74^). Preprocessing was skipped, since all necessary preprocessing steps had been performed for initial GLM-estimations. The lpMTG was defined as a thresholded cluster from a TMS-independent semantic network contrast derived from our baseline “no TMS” run. As a second ROI, we used the significant cluster in the rMFG derived from our brain-behavior regression analysis (see below). These ROIs were imported into CONN as single-timeseries seeds, and whole‑brain seed‑to‑voxel connectivity analyses were computed at the subject level. At the second level, we tested stimulation condition contrasts on gPPI effects (semantic high > semantic low) using voxelwise t‑tests within CONN. Resulting seed-to‑voxel t‑maps were thresholded at the voxel level (*p* < 0.001, uncorrected) with cluster‑level FWE correction at *p* < 0.05. Significant clusters were interpreted as regions showing condition‑dependent increases or decreases in lpMTG or rMFG connectivity.

#### Region-of-interest analyses

For in-depth exploration of the impact of TMS on the recruitment of domain-general areas of the multiple demand network (MDN), we extracted contrast estimates for the four conditions semantic high, semantic low, perceptual high and perceptual low from 7 key regions of the multiple demand network, including bilateral anterior insula (antIns), supplementary motor area (SMA), bilateral dorsal premotor cortex (PMd) and bilateral middle frontal gyrus (MFG) based on a canonical MDN reference map (^75^; https://imaging.mrc-cbu.cam.ac.uk/imaging/MDsystem). To this end, we defined 8 mm spherical regions-of-interest (ROIs) around the cluster peaks within the predefined areas^52^ using MarsBaR (version 0.45) for SPM12 (^76^; https://github.com/marsbar-toolbox/marsbar/releases). Bonferroni-corrected pairwise t-tests were then used for each ROI to compare each combination of the four conditions (*n* = 6). Supplementary analyses can be found in the Supplementary Methods and Supplementary Fig. 8. For supplementary overlap maps between task contrasts (semantic high vs. perceptual high, semantic high vs. semantic low) and MDN and SCN (Fig. 5A), we thresholded contrast images at *p* < 0.001, uncorrected with cluster‑level FWE correction at *p* < 0.05; the MDN reference map was thresholded at *t* = 1.5, and the SCN reference map was thresholded at activation likelihood estimation (ALE) = 0.02.

#### Regression and correlation analyses

To map relationships between TMS-induced modulations of task performance and task-related activity, we calculated whole-brain regressions between low intensity-normalized *z*-scored beta-estimates of the lexico-semantic conditions and the corresponding *z*-scored reaction times. Regression analyses were first performed across all trials (matches and mismatches) and then separately for both lexico-semantic conditions. This was a planned decomposition analysis, based on differences in behavioral responses from our TMS pilot (Supplementary Methods (Pilot Experiments)). Since we restricted our analysis to a small family (*n* = 2) of planned, hypotheses-driven comparisons, we did not perform multiple comparison correction for whole-brain regression models and correlation analyses at the stimulation site, consistent with recommendations by ref. ^77^ and recent complementary suggestions^78–80^.

All regression analyses were performed in SPM12 using multiple regression analysis at *p* < 0.05, cluster level FWE-corrected, with an initial cluster-defining threshold of *p* < 0.001, uncorrected. Additionally, we calculated region-of-interest (ROI) correlation analyses for the stimulation targets (6 mm spherical ROIs) between the same low intensity-normalized beta-estimates and reaction times as described above, as we expected worse lexico-semantic performance for local activity increase. One-sided tests were considered significant at *α* = 0.05.

Finally, for exploratory analyses, we extracted low intensity-normalized beta-estimates from interaction-derived (lSMG, rPrG, rSPL, rSFG) cluster-peaks of statistical maps (6 mm spherical ROIs) and correlated these with reaction times for correctly solved items (response efficiency). Exploratory analyses were FDR-corrected for multiple comparisons (semantic matches, semantic mismatches). To guarantee robustness of results, we bootstrapped significant correlations for a 95% C.I., running 5000 permutations per correlation. Bootstrapping intervals are displayed in Supplementary Fig. 7. Additionally, we calculated leave-one-out SDs (Supplementary Table 3). Supplementary regression results for the semantic high vs. perceptual high stimulation intensity conditions can be found in Supplementary Fig. 6.

### Reporting summary

Further information on research design is available in the Nature Portfolio Reporting Summary linked to this article.

## Supplementary information

Supplementary Information

Description of Additional Supplementary Files

Supplementary Data 1

Reporting Summary

Transparent Peer Review File

## Source data

Source Data

## Data Availability

Due to legal and ethical restrictions related to data privacy and the EU General Data Protection Regulation (GDPR), the datasets generated during the current study are not publicly available. Data supporting the findings of this study are available upon request to the corresponding authors and subject to a data-sharing agreement. Individual participant data are made available for the duration of the approved analyses to investigators who provide evidence of relevant ethical approval and comply with applicable German and EU data protection requirements. Access requests will be addressed as soon as possible, with a response expected within one month of receipt. Source data are provided with this paper.

## Code availability

Code used to generate figures in this article is available at https://github.com/anna-lisa-vibes/fb.
