# Supplementary material for: Disrupting the semantic network shifts activity to domain-general brain regions
Source: Nat Commun. 2026 Nov 5;17:11454. doi: 10.1038/s41467-026-79266-2 (PMC13740281; doi:10.1038/s41467-026-79266-2)
Supplement: Supplementary file 1 [file 41467_2026_79266_MOESM1_ESM.pdf]

Supplementary Information

Supplementary Methods

Adaptive Semantic Matching Paradigm

To create a comparable adaptive German version of our paradigm we followed two strategies of item generation: one semi- and one fully automatic strategy.

Semi-automatic approach: For the semi-automatic approach, we took into account age of acquisition[1,2], concreteness[3], word frequency[4] and word length. Semantic similarity was then evaluated using the dewak100k_lsa space as provided by Fritz Günther (https://sites.google.com/site/fritzgntr/software-resources/semantic_spaces). Then, the word pairs were matched manually following the criteria by[5]. The resulting items were fed into an online survey and results (accuracy and reaction times) were considered to order the items for difficulty calculating balanced integration scores according to[6].

Full automatic approach: Multiple measures were taken to tailor the stimuli of the semantic condition to German. Words were taken from word lists rated in terms of different properties, that is, word frequency, age of acquisition[1,2] and concreteness[7]. Words not included in all of the lists were excluded from further analyses. In addition to word length, word frequency, concreteness, and age of acquisition, which were used to assign levels of difficulty in the original task, we also used the criteria of orthographic neighborhood. Moreover, the original task involved manually matching word pairs and assigning them levels of difficulty, whereas we automated these processes. Using a Levenshtein distance formula, the semantic distance between the words and easiness was calculated to create the two categories of matching and unmatching word pairs, where higher level difficulty means decreasing frequency, concreteness and orthography, and increasing age of acquisition and word length. In total, our version of the semantic matching task included the 2700 most semantically related word pairs (matches), and the 2700 least semantically similar word pairs (mismatches). Finally, we organized the 2700 matching and mismatching word pairs into seven levels of difficulty, each

consisting of 300 items. These levels were interspersed with "padding" batches of 100 items to create a separation among each level of difficulty, which were excluded. These lists were then checked independently by two researchers and word pairs that were rejected by both were discarded from the final list.

After the two versions of item sets had been created, both were behaviorally tested in 15 participants. The results were comparable, however, since the fully automatic version resulted in slightly lower and less variable accuracy ratings and better reaction time scaling as a factor of difficulty level, we opted for this item set in the final experiment.

Pilot Experiments

The German version of the adaptive language mapping paradigm was piloted in 10 healthy volunteers during fMRI. Group results from two runs were analyzed with a voxelwise threshold of $p < 0.005$, and a cluster level multiple comparison correction at $p < 0.01$. These images per run were then binarized and dice coefficients for the overlap between the semantic network and the multiple demand network (MDN) were calculated. There was a low overlap between the semantic network and MDN for both runs (dice run_1 = 0.0726; dice run_2 = 0.0257), the second run showed more specific maps for semantic control with significant BOLD increases in the left IFG (inferior frontal gyrus) and posterior STS (superior temporal sulcus), exclusively. To robustly guide TMS-targeting, we calculated contrasts across both runs at the single subject level for the main experiment.

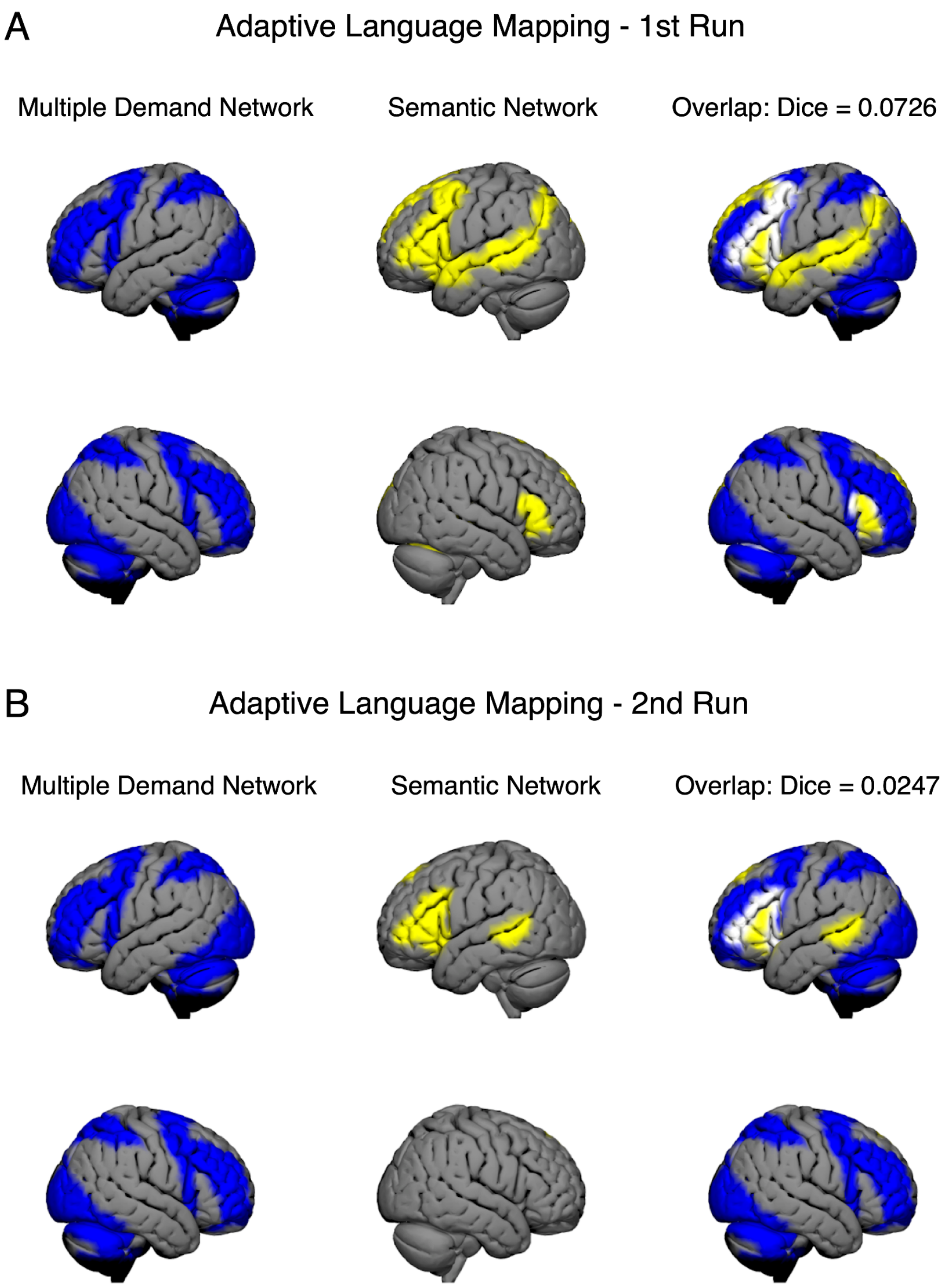

Supplementary Figure 1. Spatial overlaps between semantic and multiple demand networks in the fMRI pilot. Results of first and second runs of the German version of Adaptive Language Mapping (ALM) are overlaid on the multiple demand network (MDN, mask taken from[8]). **A.** During the first ALM run, activity was increased in the bilateral IFG, along the left superior temporal sulcus (STS) and the left premotor cortex, with a low spatial overlap to the MDN. **B.** During the second ALM run, activity was increased in the left IFG and pMTG (posterior middle temporal gyrus), with a lower spatial overlap (compared to the first run) to the MDN. Brain visualization was performed using Surf Ice.

Since timing of TMS pulses relative to stimulus onset is crucial for task interference[9], we investigated different stimulation onsets relative to visual stimulus onset in an additional TMS piloting experiment (n = 8). Based on previous M/EEG and TMS studies with similar tasks[10,11,12,13,14,15,16,17,18,19,20,21,22], we investigated delays of 200 milliseconds (msec), 300 msec and 400 msec. For piloting, task difficulty was defined as the median difficulty for semantic

and perceptual matching, respectively, during an initial baseline acquisition. We found the most robust semantic performance interference at 200 msec and thus chose this delay for the final paradigm. Performance at this timing also hinted towards qualitative differences in TMS effects–slower reaction times for mismatches high vs matches high (Cohen's d = 0.284; $p_{Wilcoxon} > 0.1$), suggesting a small but not negligible effect and lower accuracies for matches high vs mismatches high (Cohen's d = 1.605, $p_{Wilcoxon} = 0.014$) suggesting a strong effect. Descriptive statistics are displayed in Supplementary Table 1. Based on these data, we planned decomposition analyses for matches and mismatches at the behavioral level for our main experiment.

Supplementary Table 1. Descriptive TMS pilot data results at 200 msec delay

**Measure**	**Mismatches High**	**Mismatches Low**	**Matches High**	**Matches Low**
Reaction Times	1.346 sec (0.164)	1.320 sec (0.174)	1.295 sec (0.099)	1.285 sec (0.208)
Accuracy	100% (0%)	100% (12.4%)	59% (20.3%)	73% (20.2%)

All values presented as median (interquartile range, IQR). Reaction times are displayed in seconds, accuracy in percent. High = high stimulation intensity, low = low stimulation intensity.

Equivalence Tests

Reaction times were analyzed separately for each task x match condition. For each participant and condition, we computed mean reaction times for low- and high-intensity TMS and derived within-subject difference scores (high - low; positive values indicate slower responses under high intensity). Across participants, we then calculated the mean difference, standard deviation, and confidence intervals.

We evaluated within-subject reaction time differences between high- and low-intensity TMS using the two one-sided tests (TOST) procedure for equivalence testing[23]. Equivalence bounds were set to 0.2 of the raw reaction time difference scores. Equivalence was assumed when both one-sided tests were significant at $\alpha = .05$, which is equivalent to the 90% confidence interval for the mean difference lying entirely within the equivalence bounds.

Exploratory Electric Field-Behavior Correlations

To explore the effects of electric field strength on behavioral modulations, we calculated correlation coefficients (r) for low intensity-normalized reaction times (response efficiency for semantic matches and semantic mismatches) and electric field magnitude inside the >40 V/m-thresholded mask.

Network-Restricted Functional Region-of-Interest Analyses

We delineated subject-specific functional regions of interest (fROIs) using a top-percentage procedure applied within canonical masks of the multiple demand network (MDN) and the semantic control network (SCN; Fedorenko et al. 2013).

For the MDN mask definition, we started from a previously published MDN template map and thresholded it at $t > 1.5$[8]. The thresholded MDN mask and a thresholded metaanalytic SCN mask[24] were each partitioned into spatially contiguous components using “26-connectivity” in three dimensions, yielding a set of anatomical-functional “search volumes” for each network. Since the canonical MDN mask resulted in very large clusters at $t > 1.5$, we created a second mask thresholded at $t > 2$. While this allowed for better cluster separation, it had the tradeoff of smaller ROIs. For this reason, our final comparisons were calculated for both thresholds.

Subject-specific functional localization was based on an effects-of-interest contrast that depicts the combined effect across all task conditions (semantic high, semantic low, perceptual high, perceptual low) of our main experiment. We selected this contrast to minimize bias toward a specific task condition and to obtain robust localization of regions associated with multiple demand and semantic control using our dedicated interleaved TMS-fMRI coil setup. For every subject and each MDN/SCN cluster, we applied a voxelwise threshold of $p < 0.001$, uncorrected, to this contrast to remove weak or noisy responses[25]. Within the surviving voxels of a given cluster, we then retained the top 10% of voxels with the highest t-values at the single-subject level. The resulting voxel sets constituted the subject-specific MDN and SCN fROIs for each cluster.

For each subject-specific fROI, we subsequently extracted condition-specific beta estimates

from the first-level GLM. Beta coefficients corresponding to the four experimental conditions (semantic high, semantic low, perceptual high, perceptual low) were averaged across all voxels within an fROI, yielding one mean beta per subject, network (MDN vs SCN), cluster, and condition. These ROI-wise estimates were used for statistical comparisons and visualization of network- and cluster-specific response profiles.

Supplementary Results

TMS Side Effects

Supplementary Table 2. Quality, frequency and intensity of TMS side effects

Effect	Number of Participants	Range
Neck Stiffness	3	mild-moderate
Headache	8	mild-considerable
Muscle Contractions	11	mild-strong
Scalp Pain	11	moderate-strong
Difficulty Concentrating	11	mild-considerable
Fatigue	2	mild
Sleepiness	7	mild-considerable
Coil Pressure	11	mild-strong
Anxiety/Nervousness	6	mild-considerable
Intrinsic Noise Perception	1	mild
Tingling Sensations	7	mild-moderate
Skin Sensation	2	moderate-considerable
Negative Mood	2	moderate-strong
Burning Sensation	1	considerable

Baseline Results

Baseline results of the task paradigm using the interleaved TMS-fMRI setup revealed robust activity changes of the left inferior frontal gyrus (IFG) and posterior middle temporal gyrus (pMTG) as core regions for semantic processing (IFG: x, y, z = -50, 18, 24, $t(46) = 8.21$, $p < 0.001$; pMTG: x, y, z = -58, -34, 4, $t(46) = 7.56$, $p < 0.001$). This analysis was performed for 24 participants, since one participant did not perform this run due to technical problems. Semantic processing showed median reaction times of 1.07 sec (min/max: 0.80/1.34) and a median accuracy of 85%, while the perceptual control condition had median reaction times of 0.95 sec (min/max: 0.65/1.35) and a median accuracy of 95% (Supplementary Figure 2).

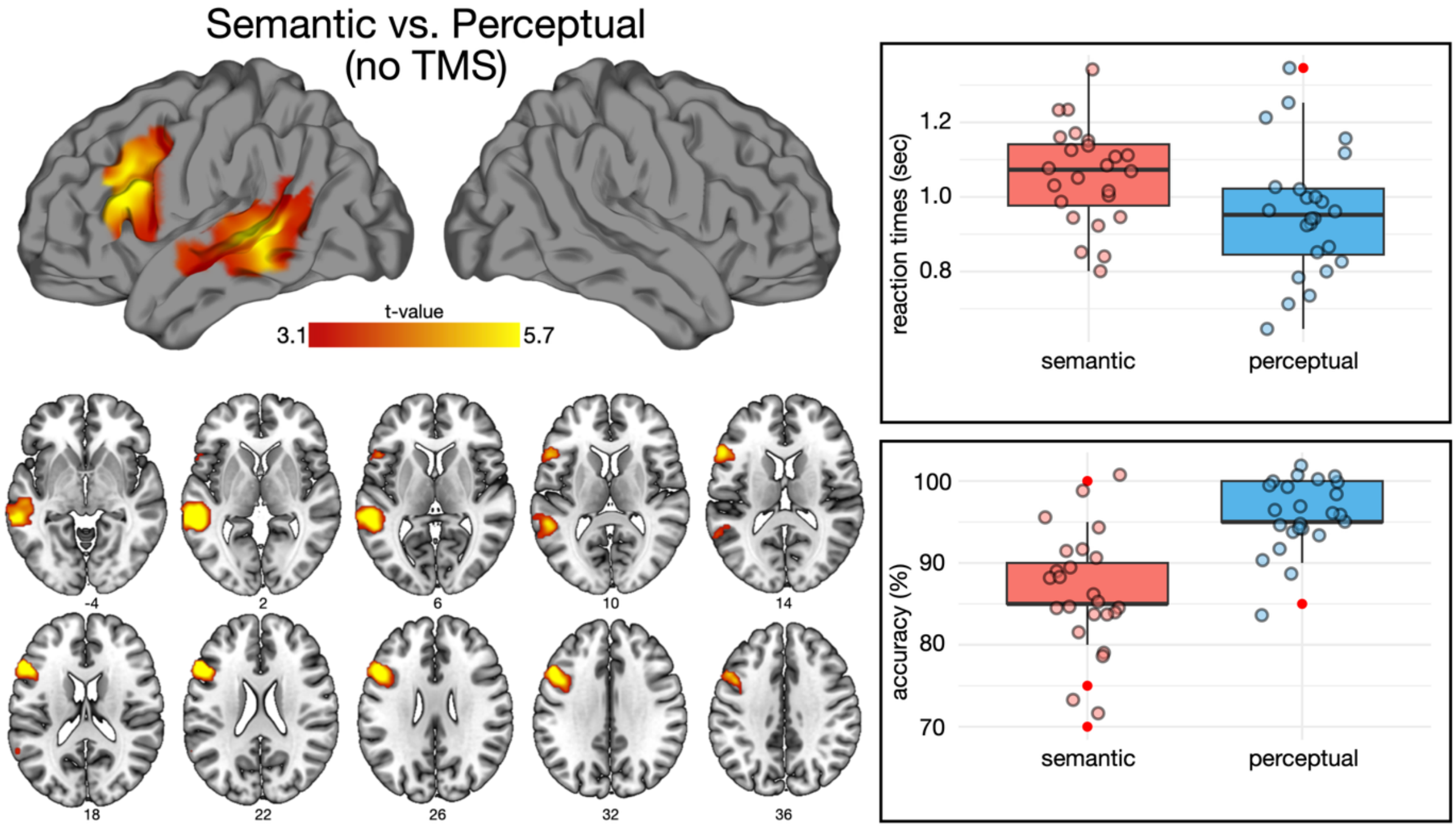

Supplementary Figure 2. "No TMS" baseline fMRI and behavioral results. Left panel. The semantic > perceptual contrast for the task run without TMS revealed activity increases in the left IFG and pMTG semantic control regions. There were no significant activity increases for the opposite contrast (perceptual > semantic). **Right panel.** Baseline reaction times and accuracy boxplots with medians (n = 24). Boxes indicate 25th percentiles, whiskers indicate 50th percentiles and red dots indicate values exceeding the 50th percentile. Brain visualization was performed using Surf Ice and MRIcroGL.

Behavioral Results

Linear mixed effects (LME) results for reaction times (RT) showed a main effect for task ($F(1, 997.23) = 166.87$, $p < 0.001$) and match ($F(1,1082.39) = 28.57$, $p < 0.001$) and a significant interaction between both factors ($F(1,1082.89) = 60.49$, $p < 0.001$). Participants were faster for perceptual mismatch trials compared to the other conditions. Simple effects were estimated with a linear mixed-effects model (Kenward-Roger degrees of freedom). Overall, the task x match interaction reflects a larger semantic - perceptual difference for mismatch trials (semantic - perceptual = 0.91, SE = 0.06, df = 941, $t = 15.07$, $p < 0.001$) compared to match trials (semantic - perceptual = 0.23, SE = 0.06, df = 914, $t = 3.55$, $p < 0.001$). The effect of match was driven by the perceptual task: for the semantic task, there was no significant difference between mismatch and match conditions (mismatch - match = 0.11, SE = 0.06, df = 1112, $t = 1.70$, $p = 0.089$), whereas for the perceptual task, reaction times were shorter for mismatch than match trials (mismatch - match = -0.58, SE = 0.06, df = 845, $t = -9.28$, p <

0.001). LME results for accuracy revealed a main effect for match ($p < 0.001$) with matching trials showing lower accuracy than mismatching trials (beta = 1.61, SE = 0.31, z = -5.25, $p < 0.001$) as well as a task-by-match interaction (beta = 2.10, SE = 0.48, z = 4.36, $p < 0.001$). Participants had less accurate performance during semantic matches compared to the other conditions. A simple-effects analysis confirmed that task had no significant effect on accuracy for mismatch trials (semantic vs perceptual: OR (odds ratio) = 1.24, z = 0.86, p = 0.39), but did so for match trials, where the odds (odds ratio) for a correct response in the semantic task were lower than for the perceptual task (semantic vs perceptual: OR = 0.19, z = -7.36, $p < 0.001$).

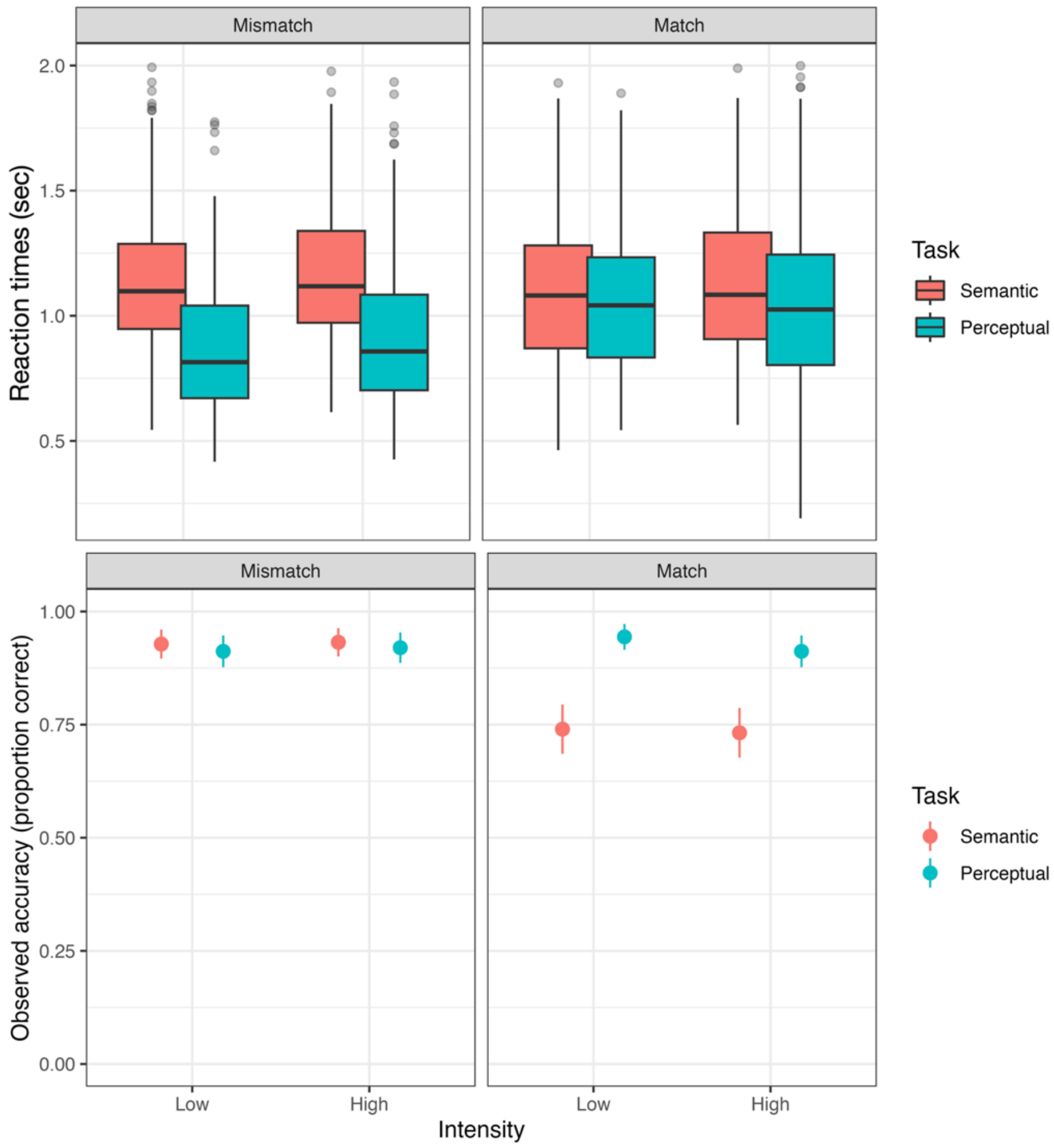

Supplementary Figure 3. Reaction times (correct responses) and accuracy during TMS. No significant differences were found between high and low stimulation intensity conditions. Participants showed faster reaction times for perceptual mismatches relative to all other conditions. Accuracy was lower for semantic matches compared to all other conditions. Boxes indicate 25th percentiles, whiskers indicate 50th percentiles and dots indicate values exceeding the 50th percentile around median reaction times. For accuracy, dots indicate mean accuracy proportion and whiskers 95% confidence intervals.

Equivalence Tests

Across all task x match conditions, within-subject reaction time differences between high- and low-intensity TMS were small and within the predefined equivalence bounds of -0.2 to +0.2. For semantic mismatches, the mean difference was 0.042 (SD = 0.127; 90% CI [-0.002, 0.085]), and the equivalence test was significant. For semantic matches, the mean difference was 0.037 (SD = 0.189; 90% CI [-0.028, 0.101]) with significant equivalence. For perceptual mismatches, the mean difference was 0.036 (SD = 0.128; 90% CI [-0.008, 0.080]) and again supported equivalence. For perceptual matches, the mean difference was essentially zero (-0.001; SD = 0.196; 90% CI [-0.069, 0.066]), and the equivalence criterion was also met.

Exploratory Electric Field-Behavior Correlations

Exploratory electric field (E-field) correlations in the broader stimulated area across participants hinted towards more posterior stimulation being associated with delayed reaction times during mismatch trials. Conversely, faster reaction times were associated with higher E-field magnitude around the posterior opercular area during semantic match trials (Supplementary Figure 4).

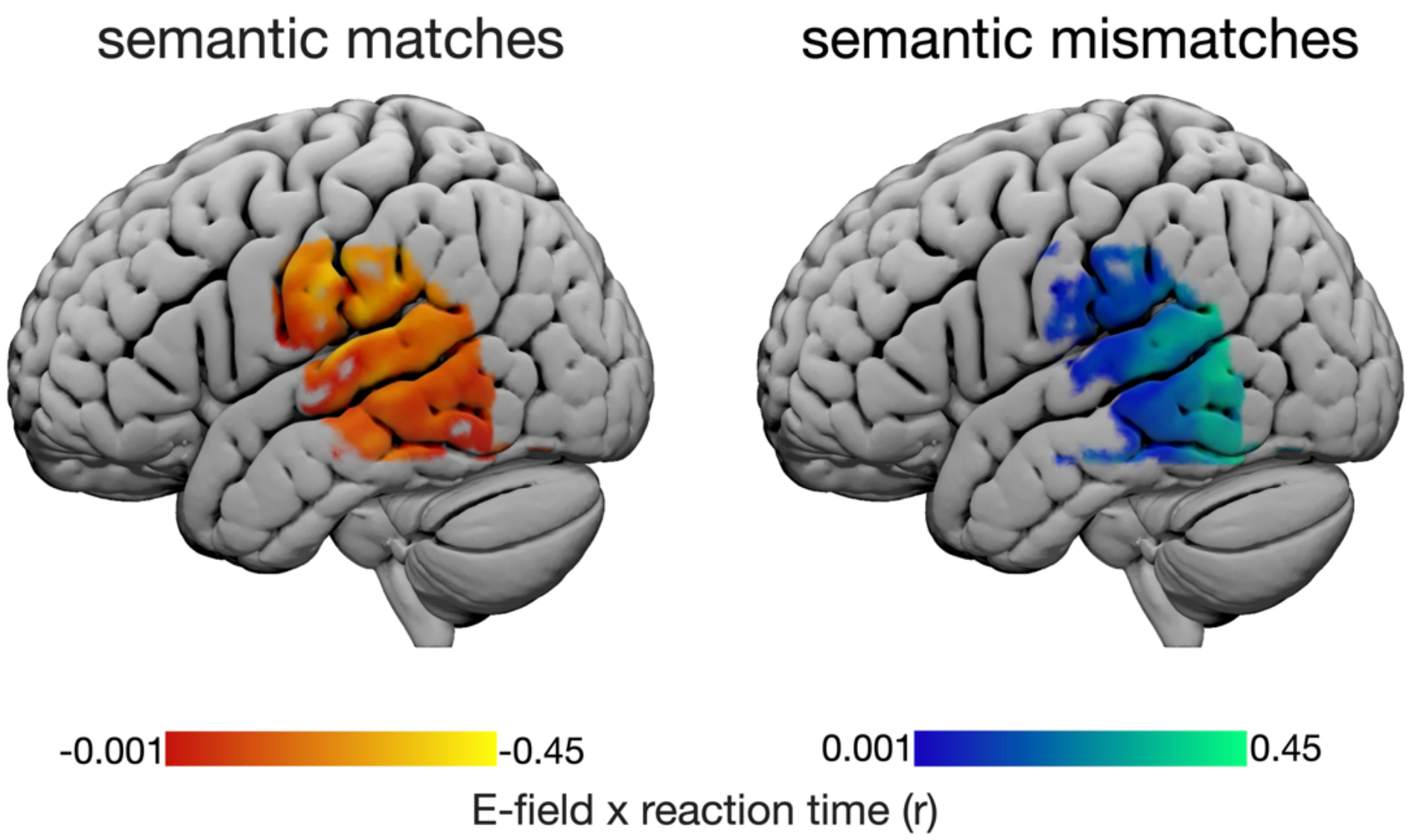

Supplementary Figure 4. Correlations between reaction times and electric field (E-field). Increased E-field magnitude was moderately correlated with increased reaction times in the more posterior part of the pMTG for semantic mismatches. Increased E-field magnitude was moderately correlated with decreased reaction times in the posterior opercular regions for semantic matches. Note: No significance testing applied. Brain visualization was performed using Surf Ice.

Brain-Behavior Correlations

For the semantic task, we found that stimulation-induced delays in reaction times were associated with increased activity in the left M1 (Supplementary Figure 5) and decreased activity in the left temporo-occipital junction (TOJ) and caudate head (Supplementary Figure 5). However, these effects did not survive whole-brain corrections for multiple comparisons.

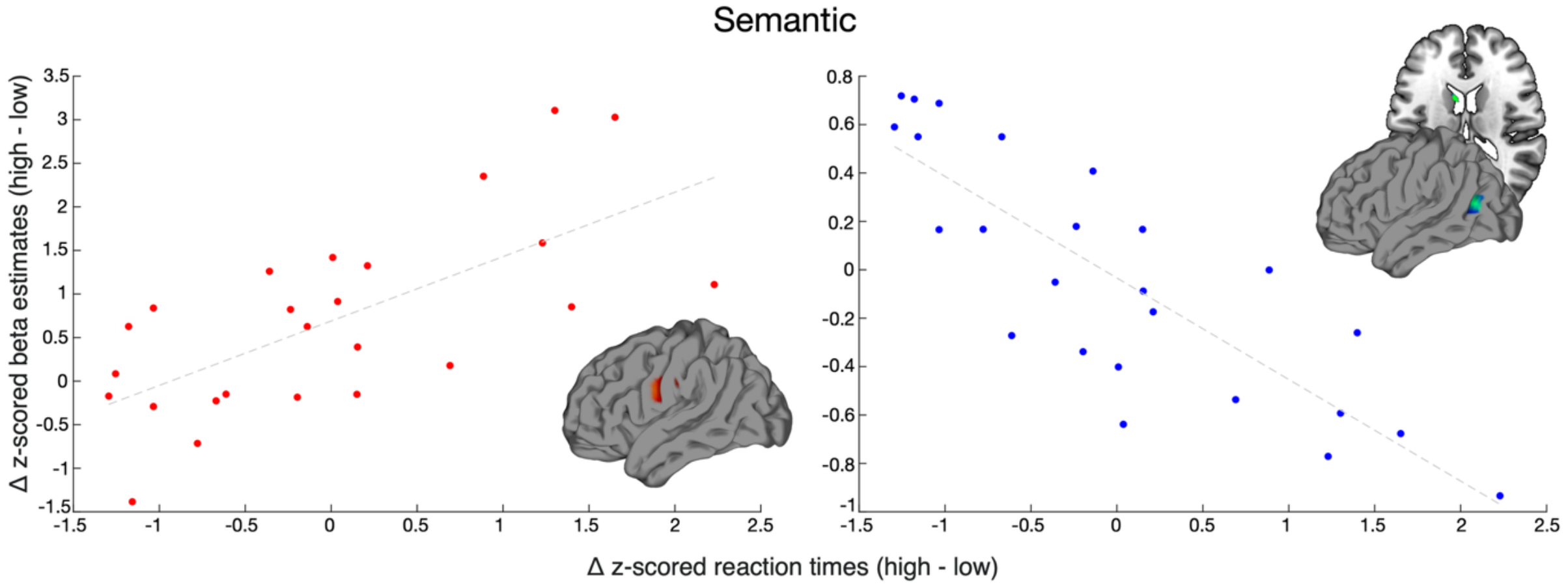

Supplementary Figure 5. Correlations between reaction times and brain activity (low intensity-normalized values). For the semantic task (matches and mismatches combined), stimulation-induced increase in reaction times was associated with increased activity in the left M1 and activity decreases in the left temporo-occipital junction and caudate head (n = 25; $p_{uncorr.} < 0.001$; voxel threshold k = 20). Brain visualization was performed using Surf Ice and MRIcroGL. Scatter plots and regression lines are depicted for illustrative purposes. Each dot represents one participant.

A regression analysis for semantic vs perceptual matches under high stimulation intensity revealed an association between increased activity in the left SPL and increasing reaction times (positive relationship). In contrast, increased activity in the left superior temporal gyrus for semantic vs perceptual mismatches was associated with decreased reaction times (negative relationship; Supplementary Figure 6). However, none of the comparisons survived multiple comparison corrections.

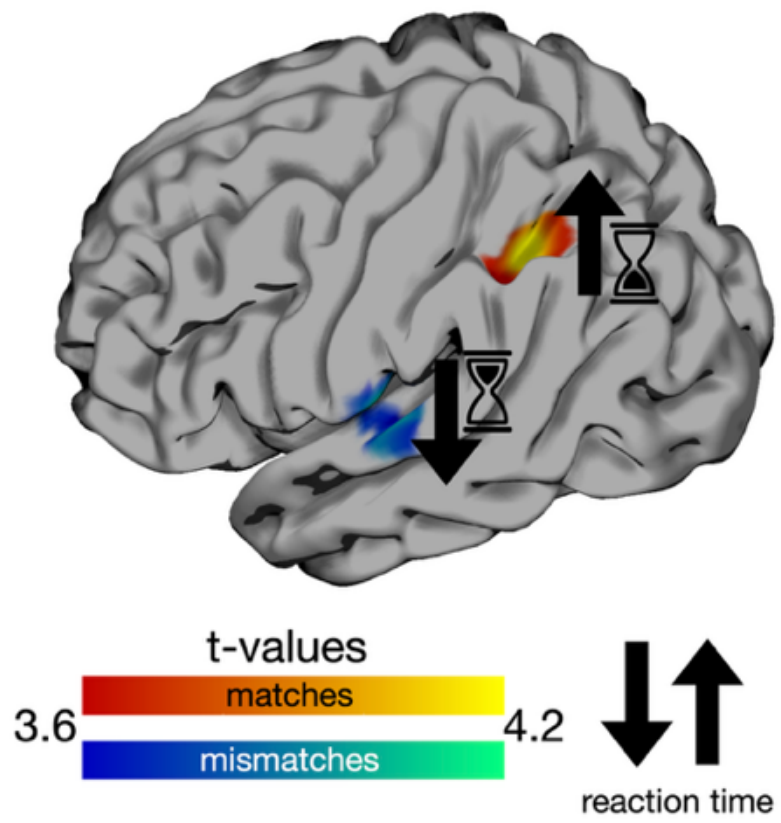

Supplementary Figure 6. Brain-behavior relationships (regression for the comparison between tasks at high stimulation intensity and behavior). Activity increases for semantic vs perceptual matches under high stimulation intensity in the left SPL are associated with increased reaction times (positive relationship). Activity increases in the left superior temporal gyrus for semantic relative to perceptual mismatches under high stimulation intensity are associated with decreased reaction times (negative relationship; n = 25; p < 0.001 uncorrected; voxel threshold k = 50). Note that these effects do not survive whole-brain correction for multiple comparisons. Brain visualization was performed using Surf Ice.

Bootstrapping Results for ROI-Behavior Correlations

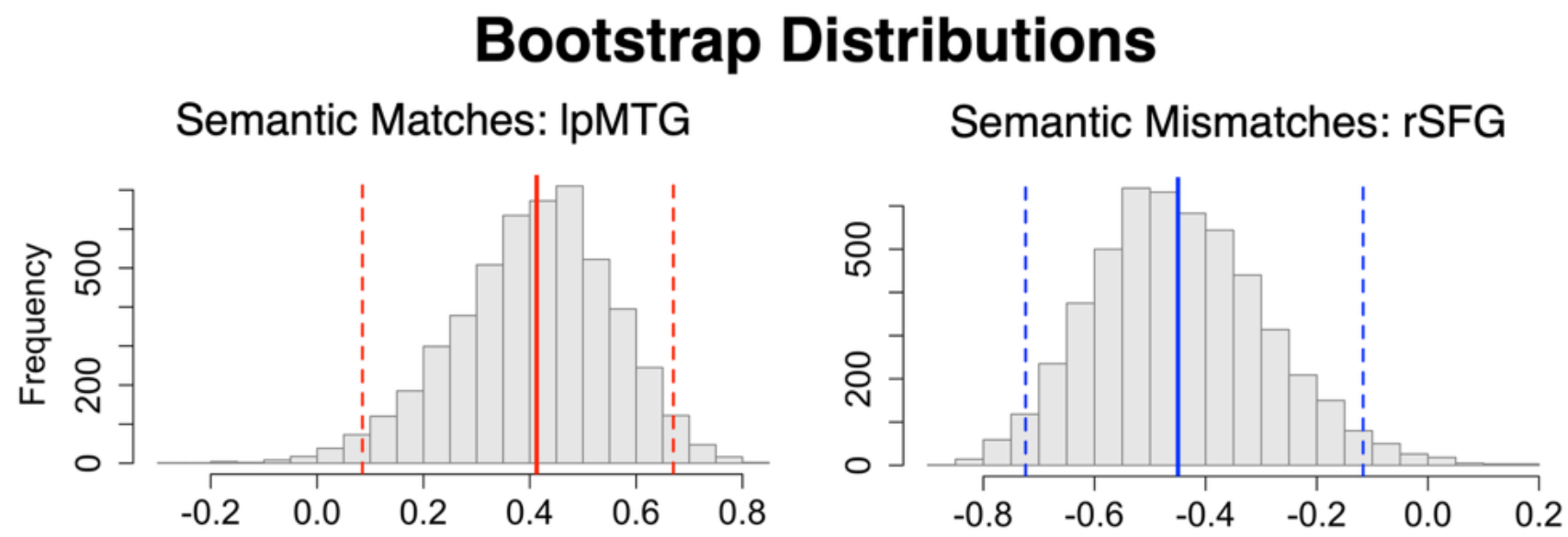

Supplementary Figure 7. Bootstrap distributions for significant brain-behavior correlations in selected regions-of-interest for semantic matches and mismatches. Left: moderate positive correlation between reaction times and activity strength (betas) in the lpMTG (stimulation target site), right: moderate negative correlation between reaction times and activity strength (betas) in the rSFG.

Supplementary Table 3. Leave-one-out and bootstrapping correlation results

ROI	Condition	loo mean	loo SD	r BS	CI low	CI high	r
	Semantic Matches						
lpMTG (stim sites)		0.413	0.032	0.413	0.085	0.67	0.413
	Semantic Mismatches						
rSFG (26 30 46)		-0.45	0.036	-0.45	-0.724	-0.117	-0.45

ROI = region-of-interest, lpMTG = left posterior middle temporal gyrus, rSFG = right superior frontal gyrus, loo = leave-one-out, SD = standard deviation, r = correlation, BS = bootstrap, CI = confidence interval

Network-Restricted Functional Region-of-Interest Analyses

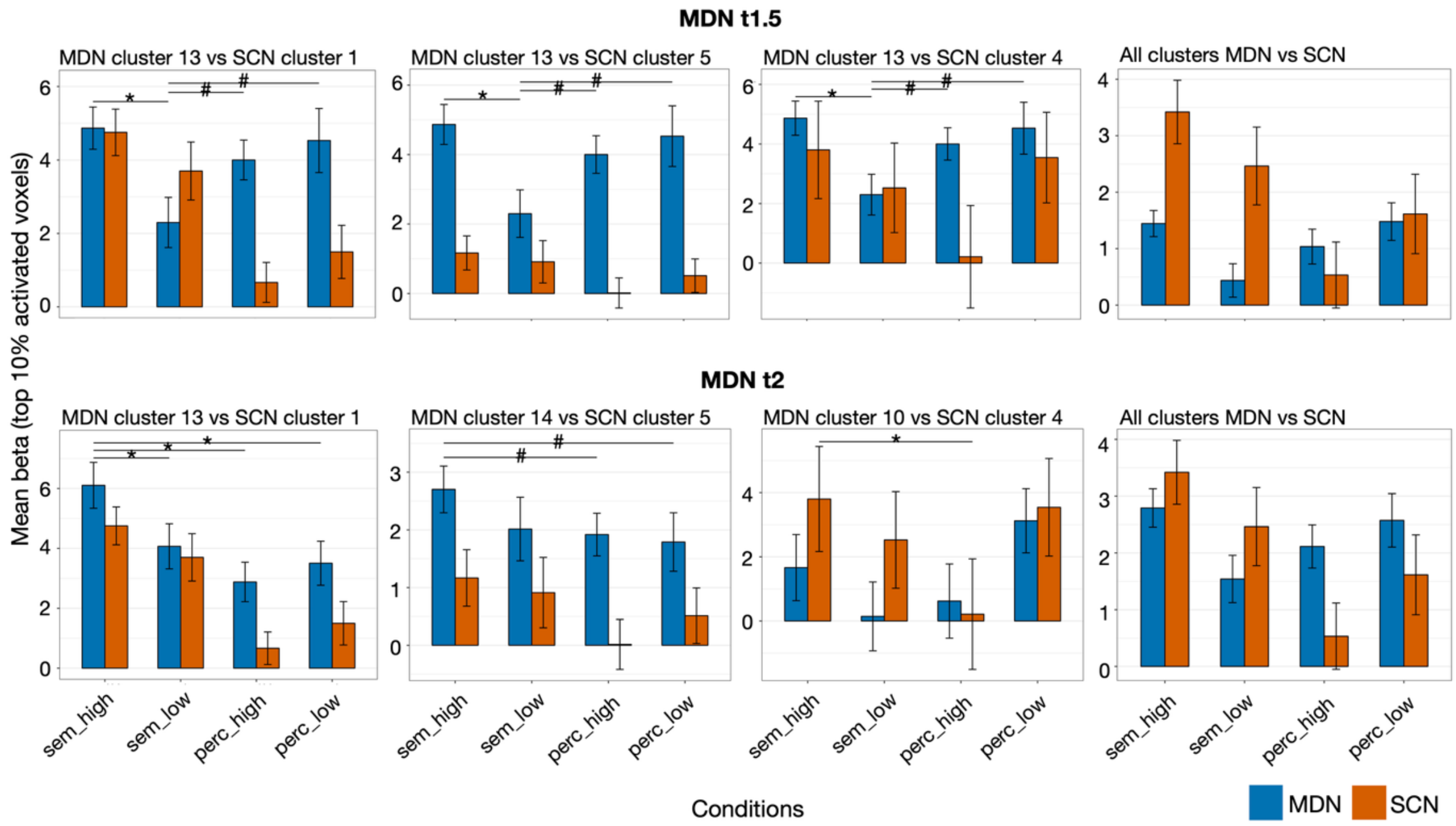

Supplementary Figure 8. Subject-specific fROI-analyses for multiple demand network (MDN) and semantic control network (SCN) overlap in selected clusters. Upper panel: Comparison within MDN network-mask thresholded at $t > 1.5$. MDN cluster 13 = frontal cluster, SCN cluster 1 = left IFG, SCN cluster 4 = right IFG, SCN cluster 5 = SMA. Lower panel: Comparison within MDN network-mask thresholded at $t > 2$ (MDNt2). MDN cluster 14 = SMA, MDN cluster 10 = right DLPFC, MDN cluster 13 = left MFG, SCN cluster 1 = left IFG, SCN cluster 4 = right IFG, SCN cluster 5 = SMA. The last figure in each row shows averaged effects across the respective mask. Bars indicate mean, whiskers indicate standard error of the mean (SEM) for n = 25 participants. # = $p < 0.05$ uncorrected, * = $p < 0.05$ Bonferroni corrected.

Interaction between intensity, task and match condition

The exploratory three-way interaction of intensity, task and match condition was not significant. However, we observed specific activity differences when considering the different conditions separately. During high-intensity stimulation, semantic matches (vs perceptual matches) resulted in activity increases selectively in the core areas for semantic control (left IFG, left pMTG/STS). In contrast, semantic mismatches revealed more distributed activity changes including anterior portions of the left temporal lobe, bilateral somatosensory and primary motor cortices and right IFG. The low-intensity stimulation condition during semantic decisions was characterized by activity increases exclusively in the left IFG for matches and in the left pMTG for mismatches, suggesting stronger semantic control for matching items and lexical search processes for mismatching items. Replicating the main analysis combining matches and mismatches, we found significant activity increases in the bilateral SMG and right

PMd for both low-intensity matching conditions and additional activity increases in the left TOJ and right dorsolateral prefrontal cortex (DLPFC) for the match condition. These results may reflect a stronger need for symbol processing and cognitive control to solve matching items.

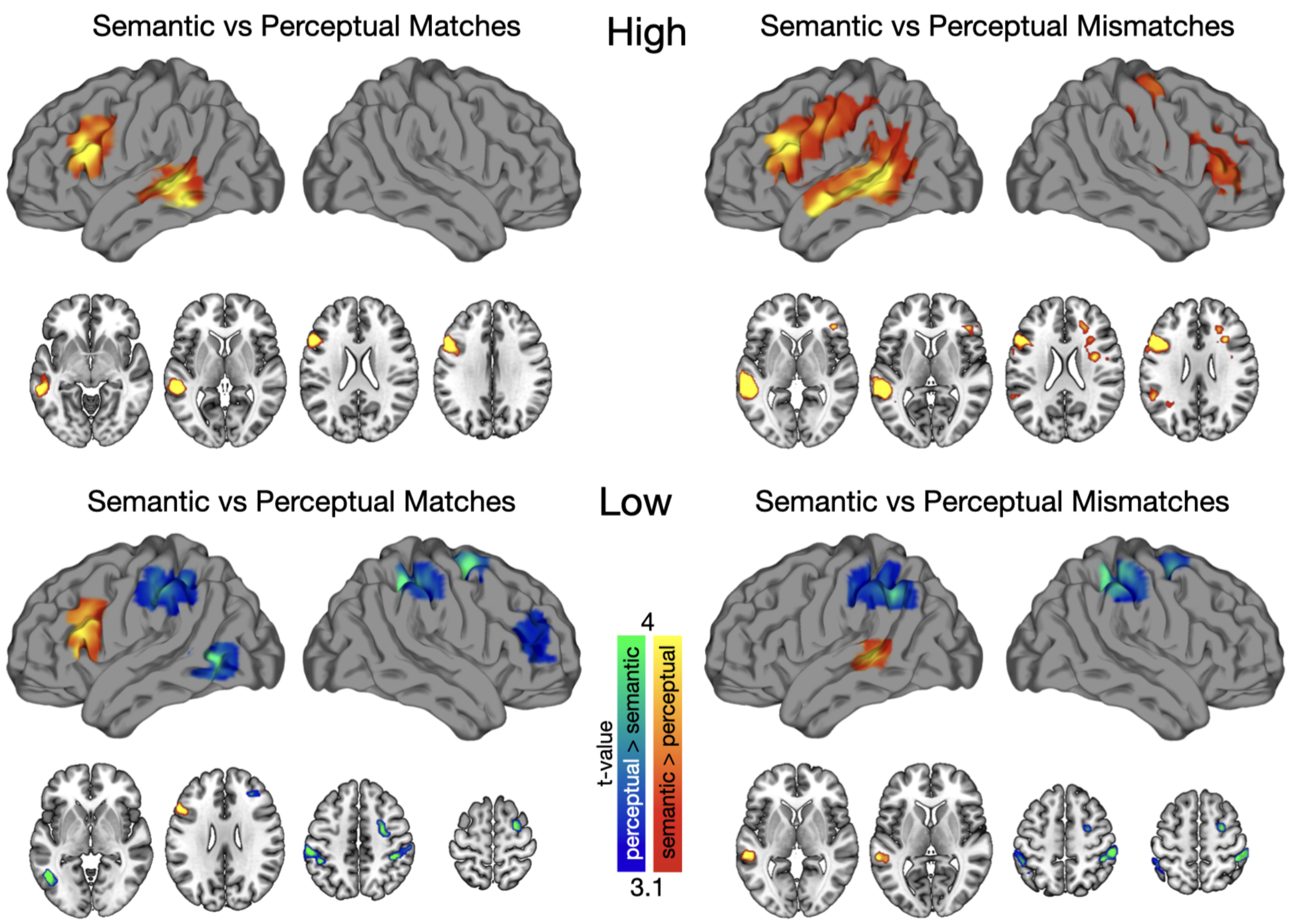

Supplementary Figure 9. Statistical maps separated for match and mismatch conditions. Activity changes for exploratory separate pairwise contrasts for intensity, task and matches. High = high stimulation intensity, Low = low stimulation intensity. Brain visualization was performed using Surf Ice and MRIcroGL.

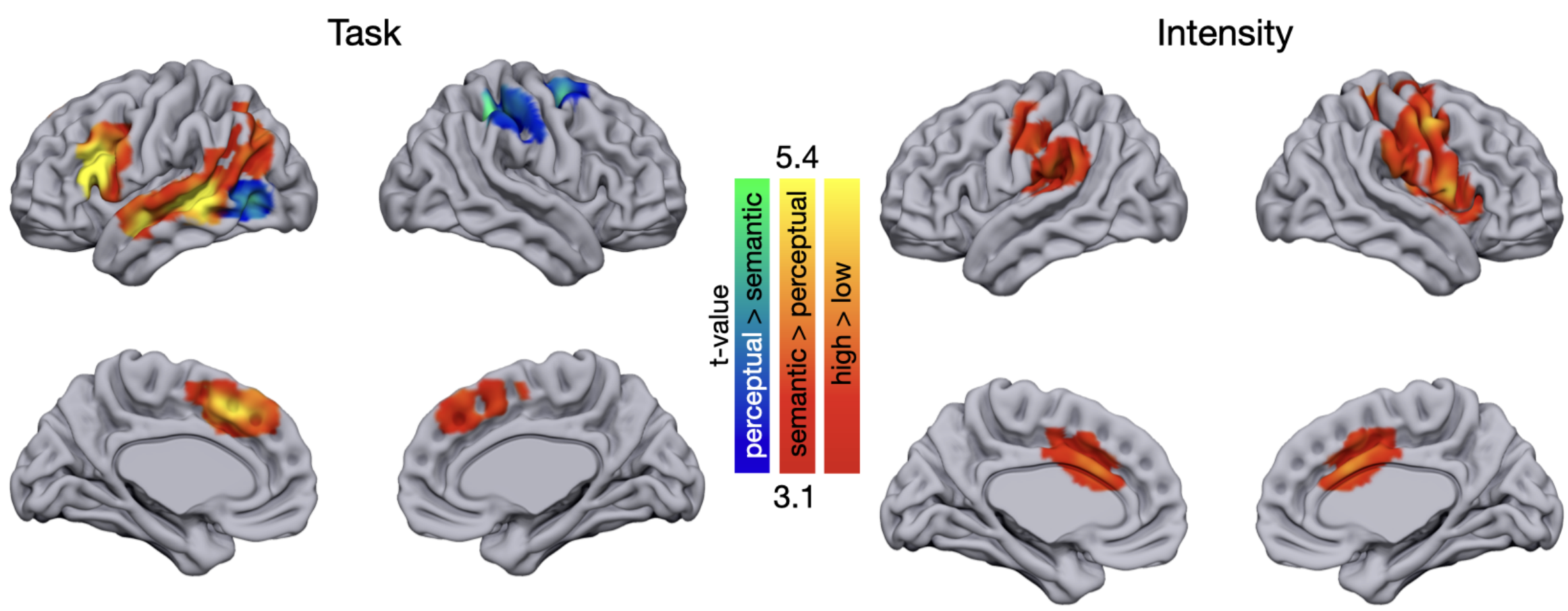

Supplementary Figure 10. Main effects of task and stimulation intensity. Task contrasts over both intensity levels revealed stronger activity in semantic control areas for semantic vs perceptual and in visual attention areas for perceptual vs semantic contrasts. High- vs low-intensity stimulation irrespective of task led to opercular, motor and somatosensory activity increases. Brain visualization was performed using Surf Ice.

Supplementary Table 4. Motion and tSNR quality checks

**Framewise displacement (translational motion parameters)**			
	**No Task**	**Task**	**Task-TMS**
mm	0.088 (0.026)	0.083 (0.026)	0.092 (0.028)
**tSNR (ROI)**			
Left MFG	35.063 (8.051)	38.119 (6.952)	30.768 (7.176)
Right MFG	46.291 (11.746)	52.619 (12.448)	37.732 (10.052)
Right SFG	42.586 (9.528)	47.837 (10.908)	36.156 (10.67)
Left OpIFG	47.847 (10.302)	53.987 (13.926)	41.637 (11.843)
Right OpIFG	45.980 (14.038)	55.652 (15.686)	38.566 (11.912)
Left TrIFG	31.345 (8.162)	36.148 (11.153)	26.780 (9.089)
Right TrIFG	34.421 (12.782)	41.431 (13.050)	30.094 (10.114)
Left MTG	37.835 (9.457)	44.510 (13.106)	30.951 (8.311)
Left STG	46.460 (11.765)	54.609 (15.797)	37.859 (10.176)
Left SMG	57.859 (15.917)	66.281 (17.562)	48.183 (15.129)
Right SMG	48.919 (12.736)	53.121 (11.062)	42.769 (13.302)
Left SPL	45.341 (9.573)	47.991 (8.591)	40.976 (9.912)
Right SPL	34.497 (11.025)	37.375 (10.741)	32.455 (11.329)

All values presented as mean (standard deviation, SD). Temporal signal-to-noise ratio (tSNR) was calculated on unsmoothed data. ROIs were defined based on Neuromorphometrics anatomical atlas.

Average subject motion was low (<1mm) and temporal signal-to-noise ratio (tSNR) was fair to good for all relevant brain areas (>20) and across the three runs.

References

1. Birchenough, J. M. H., Davies, R. & Connelly, V. Rated age-of-acquisition norms for over 3,200 German words. Behav. Res. Methods **49**, 484–501 (2017).
2. Schröder, A., Gemballa, T., Ruppin, S. & Wartenburger, I. German norms for semantic typicality, age of acquisition, and concept familiarity. Behav. Res. Methods **44**, 380–394 (2012).
3. Köper, M. & Schulte im Walde, S. Automatically generated affective norms of abstractness, arousal, imageability and valence for 350,000 German lemmas. in Proc. 10th Int. Conf. on Language Resources and Evaluation (LREC) 2595–2598 (2016).
4. Lüngen, H. & Kupietz, M. Das Deutsche Referenzkorpus DeReKo im Jubiläumsjahr 2014. Sprachreport **30**, 24–26 (2014).
5. Wilson, S. M., Yen, M. & Eriksson, D. K. An adaptive semantic matching paradigm for reliable and valid language mapping in individuals with aphasia. Hum. Brain Mapp. **39**, 3285–3307 (2018).
6. Liesefeld, H. R. & Janczyk, M. Combining speed and accuracy to control for speed–accuracy trade-offs (?). Behav. Res. Methods **51**, 40–60 (2019).
7. Lahl, O., Göritz, A. S., Pietrowsky, R. & Rosenberg, J. Using the World-Wide Web to obtain large-scale word norms: 190,212 ratings on a set of 2,654 German nouns. Behav. Res. Methods **41**, 13–19 (2009).
8. Fedorenko, E., Duncan, J. & Kanwisher, N. Broad domain generality in focal regions of frontal and parietal cortex. Proc. Natl Acad. Sci. USA **110**, 16616–16621 (2013).
9. Schuler, A.-L. & Hartwigsen, G. The potential of interleaved TMS-fMRI for linking stimulation-induced changes in task-related activity with behavioral modulations. Brain Stimul. **18**, 37–51 (2025).
10. Argyropoulos, G. P. & Muggleton, N. G. Effects of cerebellar stimulation on processing semantic associations. Cerebellum **12**, 83–96 (2013).
11. Gatti, D., Van Vugt, F. & Vecchi, T. A causal role for the cerebellum in semantic integration: A transcranial magnetic stimulation study. Sci. Rep. **10**, 18139 (2020).
12. Gough, P. M., Nobre, A. C. & Devlin, J. T. Dissociating linguistic processes in the left inferior frontal cortex with transcranial magnetic stimulation. J. Neurosci. **25**, 8010–8016 (2005).
13. Hung, Y.-h., Hung, D. L., Tzeng, O. J.-L. & Wu, D. H. Tracking the temporal dynamics of the processing of phonetic and semantic radicals in Chinese character recognition by MEG. J. Neurolinguistics **29**, 42–65 (2014).
14. Lewis, G. A., Poeppel, D. & Murphy, G. L. The neural bases of taxonomic and thematic conceptual relations: An MEG study. Neuropsychologia **68**, 176–189 (2015).
15. Passeri, A., Capotosto, P. & Di Matteo, R. The right hemisphere contribution to semantic categorization: A TMS study. Cortex **64**, 318–326 (2015).
16. Piai, V., Nieberlein, L. & Hartwigsen, G. Effects of transcranial magnetic stimulation over the left posterior superior temporal gyrus on picture–word interference. PLoS One **15**, e0242941 (2020).
17. Pineault, J. et al. A MEG study of the neural substrates of semantic processing in semantic variant primary progressive aphasia. Neurocase **25**, 118–129 (2019).
18. Rempe, M. P. et al. Alpha oscillations in left perisylvian cortices support semantic processing and predict performance. Cereb. Cortex **32**, 5376–5387 (2022).
19. Shtyrov, Y. & Pulvermüller, F. Early MEG activation dynamics in the left temporal and inferior frontal cortex reflect semantic context integration. J. Cogn. Neurosci. **19**, 1633–1642 (2007).
20. Teige, C. et al. Dynamic semantic cognition: Characterising coherent and controlled conceptual retrieval through time using magnetoencephalography and chronometric transcranial magnetic stimulation. Cortex **103**, 329–349 (2018).
21. Teige, C. et al. Dissociations in semantic cognition: Oscillatory evidence for opposing effects of semantic control and type of semantic relation in anterior and posterior temporal cortex. Cortex **120**, 308–325 (2019).

22. Zhang, Q. et al. The neural basis of semantic cognition in Mandarin Chinese: A combined fMRI and TMS study. Hum. Brain Mapp. **40**, 5412–5423 (2019).
23. Schuirmann, D. J. A comparison of the two one-sided tests procedure and the power approach for assessing the equivalence of average bioavailability. J. Pharmacokinet. Biopharm. **15**, 657–680 (1987).
24. Jackson, R. L. The neural correlates of semantic control revisited. Neuroimage **224**, 117444 (2021).
25. Nieto-Castañón, A. & Fedorenko, E. Subject-specific functional localizers increase sensitivity and functional resolution of multi-subject analyses. Neuroimage **63**, 1646–1659 (2012).
