# Supplementary material for: Disrupting the semantic network shifts activity to domain-general brain regions
Source: Nat Commun. 2026 Nov 5;17:11454. doi: 10.1038/s41467-026-79266-2 (PMC13740281; doi:10.1038/s41467-026-79266-2)
Supplement: Supplementary file 2 [file 41467_2026_79266_MOESM2_ESM.pdf]

Description of Additional Supplementary Files

File name: Supplementary Data 1
Description: Pairwise comparison performed via one-sided t-tests. All values presented as mean and standard error of the mean (SEM). Arbitrary units. P-values are Bonferroni-corrected for 6 comparisons. This table contains data from 7 frontal ROIs discussed in the main text and 3 supplementary posterior ROIs.
