# Supplementary material for: Disrupting the semantic network shifts activity to domain-general brain regions
Source: Nat Commun. 2026 Nov 5;17:11454. doi: 10.1038/s41467-026-79266-2 (PMC13740281; doi:10.1038/s41467-026-79266-2)
Supplement: Supplementary file 3 [file 41467_2026_79266_MOESM4_ESM.pdf]

nature portfolio

Corresponding author(s): Anna-Lisa Schuler, Gesa Hartwigsen

Last updated by author(s): 2026.08.30



# Reporting Summary

Nature Portfolio wishes to improve the reproducibility of the work that we publish. This form provides structure for consistency and transparency in reporting. For further information on Nature Portfolio policies, see our Editorial Policies and the Editorial Policy Checklist.

## Statistics

For all statistical analyses, confirm that the following items are present in the figure legend, table legend, main text, or Methods section.

| n/a | Confirmed | |
|---|---|---|
| ☐ | ☒ | The exact sample size (*n*) for each experimental group/condition, given as a discrete number and unit of measurement |
| ☒ | ☐ | A statement on whether measurements were taken from distinct samples or whether the same sample was measured repeatedly |
| ☐ | ☒ | The statistical test(s) used AND whether they are one- or two-sided<br>*Only common tests should be described solely by name; describe more complex techniques in the Methods section.* |
| ☒ | ☐ | A description of all covariates tested |
| ☐ | ☒ | A description of any assumptions or corrections, such as tests of normality and adjustment for multiple comparisons |
| ☐ | ☒ | A full description of the statistical parameters including central tendency (e.g. means) or other basic estimates (e.g. regression coefficient) AND variation (e.g. standard deviation) or associated estimates of uncertainty (e.g. confidence intervals) |
| ☐ | ☒ | For null hypothesis testing, the test statistic (e.g. *F*, *t*, *r*) with confidence intervals, effect sizes, degrees of freedom and *P* value noted<br>*Give P values as exact values whenever suitable.* |
| ☒ | ☐ | For Bayesian analysis, information on the choice of priors and Markov chain Monte Carlo settings |
| ☒ | ☐ | For hierarchical and complex designs, identification of the appropriate level for tests and full reporting of outcomes |
| ☐ | ☒ | Estimates of effect sizes (e.g. Cohen's *d*, Pearson's *r*), indicating how they were calculated |

*Our web collection on statistics for biologists contains articles on many of the points above.*

## Software and code

Policy information about availability of computer code

| | |
|---|---|
| Data collection | Matlab (2022b), Psychtoolbox (3.0.19) |
| Data analysis | AFNI (23.2.19), ANTS (2.5.0), SPM12 (r7771), Matlab (2022b), RStudio (2022.07.1), SimNIBS (4.5), marsbar (0.45), CONN (22.v2407) |

For manuscripts utilizing custom algorithms or software that are central to the research but not yet described in published literature, software must be made available to editors and reviewers. We strongly encourage code deposition in a community repository (e.g. GitHub). See the Nature Portfolio guidelines for submitting code & software for further information.

## Data

Policy information about availability of data

All manuscripts must include a data availability statement. This statement should provide the following information, where applicable:

- Accession codes, unique identifiers, or web links for publicly available datasets
- A description of any restrictions on data availability
- For clinical datasets or third party data, please ensure that the statement adheres to our policy

Due to legal and ethical restrictions related to data privacy and the EU General Data Protection Regulation (GDPR), the datasets generated during the current study are not publicly available. Data supporting the findings of this study are available upon request and subject to a data-sharing agreement. Source data are provided with this paper.

## Research involving human participants, their data, or biological material

Policy information about studies with human participants or human data. See also policy information about sex, gender (identity/presentation), and sexual orientation and race, ethnicity and racism.

| | |
|---|---|
| Reporting on sex and gender | Upon registration, participants self-reported their gender by selecting one of three options: 'female' (13), 'male' (12), or 'diverse' (0). We did not perform any gender-specific analyses. |
| Reporting on race, ethnicity, or other socially relevant groupings | We did not acquire data on race, ethnicity or other socially relevant groupings. |
| Population characteristics | 25 healthy right-handed volunteers (mean age= 31.04 ± 5.98 (SD), 13 female) participated in the study. Exclusion criteria were contraindications to MRI or TMS, language impairments, proficiency in any language based on the Cyrillic, Greek or Georgian writing system, psychiatric or neurological disorders. |
| Recruitment | Participants were recruited via the database of the Max Planck Institute for Human Cognitive and Brain Sciences (MPI CBS). |
| Ethics oversight | The study followed the recommendations of the Helsinki Declaration (1964) and was approved by the Ethics Committee of the Medical Faculty of Leipzig University. All participants gave written informed consent prior to participating in the study. Participants were financially reimbursed for their participation. |

Note that full information on the approval of the study protocol must also be provided in the manuscript.

# Field-specific reporting

Please select the one below that is the best fit for your research. If you are not sure, read the appropriate sections before making your selection.

☐ Life sciences ☒ Behavioural & social sciences ☐ Ecological, evolutionary & environmental sciences

For a reference copy of the document with all sections, see nature.com/documents/nr-reporting-summary-flat.pdf

# Behavioural & social sciences study design

All studies must disclose on these points even when the disclosure is negative.

| | |
|---|---|
| Study description | Data was quantitative experimental. |
| Research sample | We recruited healthy young right-handed adults (18-41 years) from the local community. We balanced our sample for male and female gender. |
| Sampling strategy | We aimed at obtaining a balanced ratio between male and female gender participants. Sample size was chosen based on previous task-based interleaved TMS-fMRI studies and was larger than average (mean = 12.76, SD = 6.57) as identified in a systematic review performed prior to this experiment (see Schuler and Hartwigsen, 2025; BrainStim). |
| Data collection | Data was collected via magnetic resonance imaging. Only experimenter was present at the experiment. Experimenter was not blind to the research questions, but real and sham stimulation was performed within one run with minimal interaction during measurement. |
| Timing | Data were collected between 2023 and 2024. |
| Data exclusions | No data were excluded from the sample. |
| Non-participation | No drop-outs. |
| Randomization | No random group allocation. |

# Reporting for specific materials, systems and methods

We require information from authors about some types of materials, experimental systems and methods used in many studies. Here, indicate whether each material, system or method listed is relevant to your study. If you are not sure if a list item applies to your research, read the appropriate section before selecting a response.

## Materials & experimental systems

| n/a | Involved in the study |
| --- | --- |
| ☒ | ☐ Antibodies |
| ☒ | ☐ Eukaryotic cell lines |
| ☒ | ☐ Palaeontology and archaeology |
| ☒ | ☐ Animals and other organisms |
| ☒ | ☐ Clinical data |
| ☒ | ☐ Dual use research of concern |
| ☒ | ☐ Plants |

## Methods

| n/a | Involved in the study |
| --- | --- |
| ☒ | ☐ ChIP-seq |
| ☒ | ☐ Flow cytometry |
| ☐ | ☒ MRI-based neuroimaging |

# Plants

Seed stocks

*Report on the source of all seed stocks or other plant material used. If applicable, state the seed stock centre and catalogue number. If plant specimens were collected from the field, describe the collection location, date and sampling procedures.*

Novel plant genotypes

*Describe the methods by which all novel plant genotypes were produced. This includes those generated by transgenic approaches, gene editing, chemical/radiation-based mutagenesis and hybridization. For transgenic lines, describe the transformation method, the number of independent lines analyzed and the generation upon which experiments were performed. For gene-edited lines, describe the editor used, the endogenous sequence targeted for editing, the targeting guide RNA sequence (if applicable) and how the editor was applied.*

Authentication

*Describe any authentication procedures for each seed stock used or novel genotype generated. Describe any experiments used to assess the effect of a mutation and, where applicable, how potential secondary effects (e.g. second site T-DNA insertions, mosiacism, off-target gene editing) were examined.*

# Magnetic resonance imaging

## Experimental design

Design type

task, event-related & block

Design specifications

16 blocks for task-tms run, 8 blocks for no tms and rest runs, consisting of 5 trials each, one trial was 1.7 seconds, ISI was 4.4 seconds, 12.2 seconds cross fixation between blocks.

Behavioral performance measures

Correct button presses and reaction times were measured. During the measurements we made sure that the participants were performing the task (checking response box) and communicated with them between the runs.

## Acquisition

Imaging type(s)

functional, structural

Field strength

3 Tesla

Sequence & imaging parameters

CMRR sequence (TR/TE = 1220/38 ms, flip angle 45°, voxel size 3 mm3, MB-factor = 4, volume acquisition gap = 520 msec; Moeller et al. 2010); MPRAGE sequence (TR/TI/TE = 2300/900/2.98 ms, flip angle 9°, voxel size 1 mm3); MP2RAGE (TR/TI1/TI2/TE = 5000/700/2500/2.85 ms, flip angle 4/5°, voxel size 1 mm3)

Area of acquisition

whole-brain

Diffusion MRI ☐ Used ☒ Not used

## Preprocessing

Preprocessing software

AFNI (23.2.19), ANTS (2.5.0), SPM12 (r7771), Matlab (2022b). 6 mm smoothing kernel was applied.

Normalization

Non-linear normalization based on MP2RAGE tissue segmentation (CAT12).

Normalization template

MNI152

Noise and artifact removal

despiking (AFNI), realignment (SPM)

Volume censoring

No volume censoring applied.

## Statistical modeling & inference

Model type and settings

First- and second-level analyses were performed in SPM12. First-level analyses included modulation of four regressors

| | |
|---|---|
| Model type and settings | (semantic high-intensity, semantic low-intensity, perceptual high-intensity and perceptual low-intensity) against the implicit baseline. To explore the impact of differences in task processing for matching vs mismatching, we set up an additional model that separated matches and mismatches for each condition, thus comprising eight regressors.<br>First-level estimates were entered into a flexible factorial design to model the main effects of stimulation intensity (high vs low) and task condition (semantic vs perceptual), as well as their interaction and pairwise comparisons. For exploratory purposes, an additional model was estimated that incorporated matches vs mismatches as a third factor. The "no TMS" run was modeled analogously, including comparisons for task conditions (semantic vs perceptual). |
| Effect(s) tested | semantic high intensity vs perceptual high intensity; semantic low intensity vs perceptual low intensity; semantic high intensity vs semantic low intensity; perceptual high intensity vs perceptual low intensity; interactions: semantic/perceptual x high intensity/low intensity; semantic/rest x high intensity/low intensity; perceptual/rest x high intensity/low intensity; |

Specify type of analysis: ☐ Whole brain ☐ ROI-based ☒ Both

Anatomical location(s): Neuromorphometrics anatomical atlas

| | |
|---|---|
| Statistic type for inference<br>(See Eklund et al. 2016) | Statistical thresholds were set to $p < 0.05$ cluster level FWE-corrected, with an initial cluster-defining threshold of $p < 0.001$ uncorrected. |
| Correction | family-wise error (FWE) correction |

## Models & analysis

| n/a | Involved in the study |
|---|---|
| ☐ | ☒ Functional and/or effective connectivity |
| ☒ | ☐ Graph analysis |
| ☒ | ☐ Multivariate modeling or predictive analysis |

| | |
|---|---|
| Functional and/or effective connectivity | psychophysiological interaction (PPI) term |
