# Supplementary material for: Disrupting the semantic network shifts activity to domain-general brain regions
Source: Nat Commun. 2026 Nov 5;17:11454. doi: 10.1038/s41467-026-79266-2 (PMC13740281; doi:10.1038/s41467-026-79266-2)
Supplement: Supplementary file 4 [file 41467_2026_79266_MOESM5_ESM.pdf]

nature portfolio

Peer Review File

Disrupting the Semantic Network Shifts Activity to Domain-General Brain Regions

Corresponding Author: Dr Anna-Lisa Schuler

This file contains all reviewer reports in order by version, followed by all author rebuttals in order by version.

Version 0:

Reviewer comments:

Reviewer #1

(Remarks to the Author)
Dear Authors,

This is a really important work in the understanding of language cortical networks and the role of each area. Especially for rehabilitation and presurgical mapping this work is important to navigate clinicians in investigating this area as well with various tasks.

Since, this team has unique expertise in applying TMS-fMRI my comments will be restricted in the manuscript itself and some minor methodological issues.

You used as high intensity the 100%MT and low 40%. Did you try to get a threshold value to apply TMS. It is known that the TMS threshold in below the MT. Did you try to define a threshold?
Why did you use the MT value?This value is usually applied in speech cortical mapping (Krieg et al., 2017; Lioumis et al., 2012) but it can be reduced also substantially (up to 70%).
The control value is 40% which is actually a very good choice because it serves as both control and sham in my opinion.

Can you talk a bit more about the timing of TMS delivery. Did this change across tasks? could it be that this playes a role in your findings? Even if this is a case this is a very important finding for both network validation and presurgical evaluation, since many times neurosurgeons do not take into consideration these very important details, and they apply unsynchronised currents during awake craniotomies resulting in very variable language-induced errors or lack of errors.

I think your work and your paradigm can be a great tool among other very few ones (the novel multi-locus TMS) to validate the current language network models. The propagation of your induced field is a very crucial tool for this purpose. In your discussion it will be important to generalize a bit, and explain how the TMS-fMRI can give further light on the how the whole network works. I think your approach can evaluate very well old and new models and categorize the functions for each area involved. This is a major paradigm shift and need to be explained in detail.

Last you miss some very important references, although you include them in reviews, but for the readership would be important to have a chance to know them. 1) https://pubmed.ncbi.nlm.nih.gov/37390891/ which introduced how TMS-fMRI can be applied and 2) the virtual lesion demonstration https://pubmed.ncbi.nlm.nih.gov/2027485/ and the first virtual lesion approach for speech cortical mapping https://pubmed.ncbi.nlm.nih.gov/22108143/ .

This is indeed an important paradigm shift study!

Reviewer #2

(Remarks to the Author)

The study employs interleaved TMS-fMRI to investigate acute neural compensation during semantic processing, a technically advanced approach that provides real-time insights into network dynamics. This addresses a critical gap in understanding how the brain adapts immediately to perturbation, complementing prior offline TMS or static neuroimaging

studies.By targeting the left posterior middle temporal gyrus (pMTG)—a core semantic control region—the authors demonstrate task-specific recruitment of both local semantic networks (bilateral IFG, SMA) and remote domain-general regions (right middle frontal gyrus [rMFG], superior parietal lobule [SPL]). The dissociation between local disruption (slower reaction times with increased pMTG activity) and remote compensation (less behavioral disruption with rMFG upregulation) is compelling.

Major Concerns
1. Stimulation Specificity and Control Conditions
TMS Intensity Validation: The low-intensity condition (40% rMT) is intended as a sham control, but the authors should clarify whether this intensity truly avoids neural modulation. Prior work suggests subthreshold TMS can still induce subtle effects, raising questions about whether "low" intensity might confound interpretations of "high" intensity effects.
Perceptual Task Specificity: The perceptual control task (inverted symbols) may not fully match the semantic task's cognitive load. The authors note higher accuracy in perceptual conditions (95% vs. 85% for semantic), indicating potential differences in difficulty. This could explain why domain-general regions (e.g., rMFG) are less engaged in perceptual tasks, rather than reflecting semantic-specific compensation.

2. Statistical and Analytical Rigor
Multiple Comparisons: Whole-brain analyses use cluster-level FWE correction, but ROI analyses (e.g., MDN regions) rely on Bonferroni correction without pre-registration of ROIs. The overlap between a priori semantic control regions and exploratory whole-brain results (e.g., rMFG) should be explicitly justified to avoid circularity.
E-Field Simulations: While E-field magnitudes confirm pMTG targeting, the relationship between E-field strength and behavioral effects (e.g., slower reaction times in posterior pMTG for mismatches) is only briefly mentioned. A more detailed analysis of E-field-behavior correlations across the stimulated region would strengthen causal claims.
3. Interpretation of Compensatory Mechanisms
Domain-General vs. Semantic Network Interactions: The authors argue that rMFG upregulation reflects "compensation," but this could also reflect task-general effort (e.g., increased attention). Control analyses contrasting semantic vs. non-semantic high-load tasks (e.g., working memory) would clarify whether effects are truly semantic-specific .
State-Dependence: The interaction between TMS intensity and brain state (semantic task vs. rest) shows rSPL and rSFG effects, but the functional relevance of these regions to semantic processing is unclear. Linking these to existing models of semantic control would strengthen interpretations.

4.Despite low spatial overlap between the semantic network and Multiple-Demand Network (MDN; Dice coefficients 0.0247–0.0726), ROI analyses show MDN activity increases. The impact of multiple comparison correction on small effect sizes needs explicit discussion.Some correlation analyses have a small sample size (n=25), e.g., the correlation between rSFG activity and reaction times in semantic mismatch trials ($r=-0.450$, $p=0.024$). Effect size reporting or bootstrap validation is advised.

Minor Revisions
1.Clarify Stimulus Design: The adaptive staircase procedure for German word pairs is well-described, but details on how "semantic high/low" and "perceptual high/low" load were operationalized (e.g., difficulty levels, Levenshtein distance) should be expanded in the main text.
2.Visualization of Brain-Behavior Correlations: Scatter plots (e.g., Figure 2C) would benefit from individual data points and 95% confidence intervals to illustrate variability in the relationship between rMFG activity and reaction times.

This study provides novel insights into acute neural compensation during semantic processing using interleaved TMS-fMRI. Addressing concerns about stimulation control, statistical rigor, and mechanistic interpretation will strengthen its impact. The work has significant potential to advance understanding of flexible network dynamics and inform neurorehabilitation strategies.
Recommendation: Major revision

Reviewer #3

(Remarks to the Author)
Schuler et al. used the interleaved TMS-fMRI paradigm to investigate stimulation-related activity changes during a semantic matching task (with perceptual matching as a control). To do so, they target a functionally localized region in pMTG. By comparing the effect of high-intensity stimulation in semantic vs. perceptual conditions, they found the task-specific increase in left pMTG, bilateral IFG, and SMA, regions associated with semantic control (SC) in the past literature. Meanwhile, high vs. low stimulation comparison primarily increased activity in the domain-general multiple-demand (MD) network, with partial overlap with SC regions. Moreover, the authors posit a relationship between TMS-induced activity changes and behavioral performance: increased responses in the left pMTG are associated with performance disruption, whereas stronger activity in the MD regions is associated with less delay responses.

The goal and design are clear, and the use of simultaneous TMS and fMRI is innovative. However, we have substantial reservation about the study's analyses and interpretation of the results.

Major points:

- Lack of distinction between language and semantic control. The authors frame their study as a study of semantic control areas; however, the semantic condition uses words whereas the perceptual condition uses foreign alphabet strings,

meaning that the contrast includes language areas - many of the areas identified by the contrast might have been identified even during passive viewing of these stimuli. Moreover, the stimulation target is defined using that same contrast within pMTG, a region that contains swaths of language-selective contrast, so it's very possible that it's a language area, not a semantic control area that's being stimulated. Further, even if the target itself was a semantic control area and not a purely language area, I am not sure that TMS is spatially precise enough to only stimulate one and not the other, given the functional heterogeneity of pMTG. The authors state that their findings have implications for aphasia, which is a disorder of language - so do they make claims about the language regions or semantic control regions?
To clarify why this distinction matters: language areas would be engaged during language comprehension/production whether or not there is a semantically demanding task; in contrast, semantic control areas would be preferentially recruited during semantically challenging tasks but wouldn't show a modality specificity, responding to both linguistic and non-linguistic semantically meaningful stimuli. So, the question of which network is being targeted would have important implications for both understanding the brain and developing strategies for treating aphasia, but it is not addressed here.

- Lack of functional localization when describing the results. One of our main concerns is how regions are assigned to different functional networks. For instance, is right IFG a SC region, an MD region, or a language region? As noted in the first paragraph of the Results section, significant activation for Semantic>Perceptual in the baseline condition does not include right IFG. While left IFG is consistent and reliably identified as a SC region, right IFG is smaller and MD activation is stronger on the right frontal cortex (Jackson et al., 2021). Similar concern also applies to SMA. In particular, SMA is mentioned as part of the core SC network in Figure 2, but later as one of the MD regions in Figure 4.

Related, line 138 mentions that Semantic high>Perceptual high activates domain-general regions. What are these regions? Figure 2A only denotes regions for the SC network. In short, currently it is unclear to me whether and to what extent there's selective engagement of the MD regions during the semantic condition after effective stimulation. Left MFG ad SMA perhaps? But I will be careful to conclude these are MD regions, given the decent of overlap between the MD and the SC network on the left hemisphere and in those anatomical regions (Jackson et al., 2021).

Realistically, these broad anatomical areas are functionally heterogeneous and likely include both SC and MD subareas; distinguishing between them would require analyzing responses at the participant level (with relevant localizer tasks or resting-data-based network parcellations). Thus, the group-level analyses employed in this study have somewhat limited power for dissociating effects in MD, SC, and language networks.

- Unclear multiple comparisons in brain-behavior analyses. Showing an association between behavioral responses and TMS-induced brain changes is cool, but the current results seem post hocky, at least in the way they are currently described. Why are local effects only observed for matching trials and remote effects only for mismatching trials? Why do we see increased activation post TMS at the local point impairing performance - from a theoretical standpoint, wouldn't we predict the opposite? How exactly was the whole-brain analyses done for Figure 2C and is the same data used to define that region used to estimate the correlation (which would constitute double dipping and potentially inflate the effect)? Overall, how many comparisons were done in total, were they exploratory or hypothesis-driven, and how was correction for multiple comparisons done?

- Describing and interpreting interaction effects. Why are the brain maps in Figure 3 A and B so different? Wouldn't we expect a consistent effect of increased stimulation, no matter whether the semantic task is contrasted with a perceptual baseline or rest? Is this result surprising or theoretically justified? Methodologically, I assume the interaction effects are estimated using the same data as the data that was used to define the clusters, which is an instance of double dipping and is likely to inflate the effect. Finally, there is only one scatterpot per panel, for 1/2 brain areas and 1/2 trial types (match/mismatch). Was multiple comparison done on these and the rest of the comparisons, that I assume went unreported?

- Is there a significant performance difference between semantic and perceptual conditions during the baseline condition? SC regions are usually defined by contrasting hard vs. easy semantic conditions (e.g., Noonan et al., 2013). This is supposed to isolate the semantic-specific controlling component. The current contrast, semantic > perceptual, might yield regions that for semantic cognition and difficulty in general. This may explain why we observe an effect in the left anterior temporal cortex and angular gyrus, beyond the core SC network.

Other points:

It would be helpful to talk about the rationale for using 40% rMF as the low-intensity control, especially considering that this condition increases activity in the perceptual but not the semantic condition. How does this yield the same or different effect as the sham control?

In the Discussion, the potential anterior-to-posterior gradient is discussed, but it is unclear how this relates to any results reported in the Results section.

Later in the Discussion (line 362-364), the authors mentioned that mismatched trials are more complex and demanding. Is this reflected in behavioral performance? Can this be supported by SI Figure 6, where more left anterior temporal activation

in high mismatched vs. matched? Related, in the Supplementary Brain-Behavior Correlations section, here, the behavior is the matched and mismatched combined? The rationale for separating or combining different types of trials can be clearer.

Line 339-341: “While the effect in the SPL was explained by increased activity solely for the effective high intensity stimulation during the semantic task, the effect in the SFG was driven by decreased deactivation during the same condition.” The effect in the SFG is also driven by stronger deactivation during Rest in high stimulation.

The paragraph from line 345 to line 356 mainly reiterates results. It would be helpful to discuss how to interpret these differential response patterns in MD ROIs.

Line 375-378: According to this logic, do we expect to see a correlation between decreased activity in motor areas and increased activity in SC regions? Is there any significant correlation?

Minor points:
line 255: “less engagement for the semantic low stimulation …” which region? rPMd?
Figure 5: It would be helpful to mark the names of regions that are associated with delayed or less delayed responses
Line 364-367: How does the state dependency of the TMS effects for the semantic task relative to rest support the claim in the previous sentence (i.e., mismatched condition creates a more vulnerable brain state)?

Overall, the conceptual conflation between language and semantic regions, the methodological difficulty of assigning brain regions to functional brain networks in the absence of functional localization, and the statistics issues unfortunately leave us with substantial doubts about the results presented in this work.

Reviewer #4

(Remarks to the Author)
Overall summary

The authors use interleaved TMS–fMRI to test how the brain responds immediately to perturbation of a key semantic-control node (left pMTG) during an adaptive semantic matching task. The approach is technically ambitious (delivering bursts within a ~520 ms acquisition gap) and the design is unusually strong for causal neuroimaging: a within-subject manipulation with multiple, complementary controls (perceptual control task, intensity manipulation, a no-TMS baseline, and a stimulation-at-rest run). The central pattern – local semantic-network effects linked to slowing, alongside remote domain-general recruitment linked to preserved performance – is compelling and fits well with prominent network-flexibility accounts. More broadly, this work is a methodologically impressive application of interleaved TMS–fMRI to semantic control and is well positioned to serve as a useful reference for acute network adaptation during language/semantic processing.

The manuscript is already strong. In my view, the main claims would be further strengthened primarily by: (i) making the handling of peripheral/sensory confounds even more explicit in the main narrative using controls the authors already include (especially the rest run and task control), and (ii) adding (or briefly motivating why not feasible) a connectivity-based test of the proposed compensatory mechanism linking right frontal recruitment to semantic-network support. With these clarifications/additions, I expect this paper will make a strong contribution to both the concurrent TMS–fMRI and semantic cognition literatures.

Major comments

1) Peripheral/sensory confounds: make the logic maximally explicit using the existing controls. A key interpretive challenge in concurrent/interleaved TMS–fMRI is that “high vs low” intensity can differ in auditory/somatosensory sensations and arousal/startle, which may influence behavior and BOLD responses in nonspecific ways. The authors already acknowledge these issues and, importantly, the current design is well equipped to address them (perceptual control task and stimulation-at-rest run). I recommend making this inferential logic especially explicit in the main Results/Discussion so readers can immediately see why the key conclusions are not driven by generic intensity effects.
Suggestions:
• Center the inference on state-/task-specific effects (e.g., Task × Intensity interactions and/or semantic vs rest comparisons), rather than emphasizing simple high>low maps. Even if the underlying model already includes this, showing it clearly (main text and/or a compact figure panel) would help readers understand how nonspecific intensity consequences are controlled.
• Elevate the rest-run check in the main narrative (rather than leaving it feeling secondary). A concise statement like “effects consistent with compensation are state-dependent and not present (or are qualitatively different) at rest” would go a long way.
• Discomfort/pain ratings: If subjective discomfort/loudness ratings were collected, reporting them (even briefly) would further strengthen this point. If not collected, a one-sentence limitation acknowledging this would be sufficient.

2) Make the “compensation” interpretation more directly testable with connectivity. At present, compensation is inferred primarily from (i) remote upregulation in domain-general regions and (ii) brain–behavior associations in right frontal areas. This is promising and theoretically well motivated. However, a connectivity analysis would make the mechanistic interpretation substantially more persuasive, because it would test whether the “helpful” remote recruitment is functionally

integrated with the semantic network during perturbation.
Suggestion:
• Add a task-dependent connectivity analysis (e.g., PPI/gPPI), using seeds in right MFG/rSFG and/or canonical semantic-control nodes (e.g., left IFG/pMTG). The key prediction would be stronger coupling between right frontal regions and semantic-control/semantic areas specifically during high-intensity semantic blocks (and/or higher-demand conditions).
• If full PPI is not feasible, a focused ROI-to-ROI connectivity analysis (semantic-control ↔ MDN nodes) would still directly test redistribution accounts.
• If connectivity is not feasible (e.g., design constraints, power), a brief statement explaining why would help align expectations with what the current data can support.
3) Clarify ROI definition/independence (preempt circularity concerns). The MDN ROI findings are interesting and support the domain-general recruitment narrative, but you clarify how ROIs were defined and whether they were independent of the contrasts used for hypothesis testing.
Suggestions:
• Add a clear statement specifying whether ROI centers are drawn from an independent atlas/prior coordinates versus derived from peaks in the present contrasts.
• If any ROIs were derived from the present data, consider a leave-one-subject-out approach or a fully atlas-based alternative for the main confirmatory ROI tests (with current ROIs retained as exploratory).

4) Brain–behavior correlations: clearly distinguish confirmatory vs exploratory and state the correction approach. Multiple brain–behavior correlations are reported (local and remote; sometimes split by match/mismatch). Given their importance for the mechanistic story, we would benefit from knowing which relationships were hypothesized a priori and how the paper handles the multiplicity of tests.
Suggestions:
• Label correlations (or families of correlations) as confirmatory vs exploratory
• Briefly state the strategy for multiple comparisons (e.g., FDR across a defined family, or a small hypothesis-driven set highlighted in the main text with the remainder described as exploratory).
• Where feasible, consider supplementing difference-score correlations with a single regression-style framework (e.g., predicting RT or inverse-efficiency with Task/Intensity/Match and relevant interactions) to reduce noise and simplify interpretation.

5) Alternative behavioral modeling. The behavioral results reassuringly show minimal (or no) high vs low differences in RT/accuracy within task, which supports the interpretation that neural effects are not trivially explained by gross behavioral differences. Because the mechanistic argument leans on brain–behavior relationships, a trial-level model could provide additional leverage and a clean way to integrate Task/Intensity/Match in one place.
Suggestions:
• Consider adding a trial-level mixed-effects model (LME/GLMM) with Task, Intensity, Match, and interactions, with subject-level random effects. This uses all trials and avoids some limitations of condition medians.
• If the authors want to emphasize “no behavioral confound,” an equivalence test (or Bayes factors) for high vs low within each task can communicate evidence for no meaningful difference (rather than a null result).

6) Interpretation: sharpen domain-general control vs arousal/difficulty and keep match/mismatch aligned with evidence strength. The authors already makes a nuanced argument about state dependency and demand (e.g., mismatch trials). I recommend a brief expansion that explicitly contrasts two interpretations and ties them to the existing controls.
Suggestions:
• Arousal/startle/peripheral effects: should be broad (semantic + perceptual) and potentially present at rest.
• Domain-general control compensation: should be state- and demand-dependent, and should relate to preserved performance.
Relatedly, if the key Intensity × Task × Match interaction is not significant, I recommend keeping the main claims centered on supported effects and framing match/mismatch decompositions as exploratory unless clearly powered and pre-specified.

Minor comments / clarity edits
• Define “semantic efficiency” at first use (e.g., correct RT / inverse-efficiency) and keep terminology consistent.
• If follow-up contrasts are presented after a non-significant higher-order interaction, label them clearly as planned decompositions or exploratory (as appropriate).
• Consider adding one brief sentence situating the semantic-control framing within a standard model (e.g., controlled semantic cognition) for general readers.
• Ensure motion/tSNR quality checks are easy to find (a compact table or brief summary is sufficient).
• Make the rationale for the cluster correction approach explicit (e.g., CDT $p<.001$ with cluster-level FWE), primarily to preempt predictable reviewer concerns.
• Briefly justify the choice of 40% RMT as the control intensity and clarify what it controls (and what it does not).

Reviewer #5

(Remarks to the Author)
I co-reviewed this manuscript with one of the reviewers who provided the listed reports. This is part of the Nature Communications initiative to facilitate training in peer review and to provide appropriate recognition for Early Career Researchers who co-review manuscripts.

Version 1:

Reviewer comments:

Reviewer #1

(Remarks to the Author)
Dear Authors,
Thank you for the comprehensive answers to all te comments of all the reviewers. Indeed your work is important and pioneering!

I have one more suggestion, that can be ignored. Can you please simplify the title a bit.
Although it is detailed and informative it can be difficult for a more general readership, although it is indeed a difficult task

Reviewer #2

(Remarks to the Author)
I have read the revised manuscript and the authors' responses to the reviewers' comments with interest. Overall, I think the authors have made a substantial effort to address the concerns raised during review. The revised manuscript is clearer in its theoretical motivation, better structured in its presentation of the control conditions, and more cautious in several places regarding the interpretation of TMS-induced activity changes as compensatory mechanisms.

I still think several points should be treated with caution before publication.

First, the term "compensatory" remains somewhat stronger than what the data can directly establish. The negative brain–behavior correlations in right frontal regions are consistent with compensation, but they do not by themselves demonstrate a causal compensatory mechanism. This is especially important because BOLD activity cannot determine whether the underlying neural activity is excitatory or inhibitory, a limitation that the authors themselves acknowledge. I therefore recommend that the authors consistently use more cautious language, such as "putative compensatory recruitment" or "activity patterns consistent with compensation," particularly in the Abstract, Discussion, and Figure 6 legend.

Second, the sample size remains relatively modest for individual-difference correlations between stimulation-induced BOLD effects and behavioral modulation. The reported correlations are theoretically interesting, but some are close to conventional significance thresholds. The manuscript would benefit from a clearer statement that these brain–behavior associations should be interpreted as converging evidence rather than definitive proof of compensation. This is particularly relevant for the planned match/mismatch decomposition and for the decision not to correct some planned correlation analyses for multiple comparisons.

Third, while the multi-level control framework is a strength, the low-intensity stimulation condition cannot fully separate auditory/somatosensory effects from intensity-dependent neural effects, as the authors acknowledge. This limitation should remain visible in the Discussion rather than being treated as fully resolved by the design.

In summary, I find the revised manuscript substantially improved and scientifically valuable. The study offers a technically sophisticated and novel contribution to the literature on semantic control, TMS-fMRI, and flexible network adaptation. Provided that the authors further moderate the causal/compensatory language and explicitly acknowledge the remaining limitations concerning sample size, correlational interpretation, and translational claims, I would support publication after minor revision.

Reviewer #3

(Remarks to the Author)
All reviewers note the technical innovation of the work, which is indeed commendable. However, our concerns here center primarily on the scientific (statistical) validity of the findings, as well as the theoretical framing. The authors state that their results might be relevant for aphasia treatment development, and so it's really important to be confident in the implications that this work does or does not have for aphasia, as well as the overall strength of the work. We are worried that, if published in a high-impact journal like Nature Comms, the results of the work might be perceived as stronger as they are. We are listing the core concerns below, in order of importance.

The link between brain response and behavior. In our original review, we asked which analyses were prespecified and which were exploratory, and whether adjustments for multiple comparisons have been made. The authors responded that they fit separate models for correct and incorrect responses based on pilot data. However, their own method description says that the main model does not include response correctness and that "For exploratory purposes, an additional model was estimated that incorporated matches vs mismatches as a third factor." Thus, this result is purely exploratory, which is not reflected in the framing. It is also not quite consistent with the authors' original hypothesis, which does not posit different effects for matches and matches - that fact too should be reflected. Finally, a multiple comparison correction should be done here and was not - the authors run separate tests for matches and mismatches and even if only one is significant, they treat it as evidence in support of their theory. This is a classic situation where a multiple comparison adjustment would be needed - and if applied, it might actually make one of the results non-significant. So overall, we just don't have faith in the brain-behavior results and the replicability of these findings.

Semantic vs linguistic processing. MTG, the stimulation site, may relate to either linguistic processing or semantic control. Thus, when it is disrupted, either linguistic or semantic processing may be affected, which would have implications for different symptom profiles in aphasia. The authors respond to our original framing concern by stating that semantic processing can take place over either linguistic or non-linguistic stimuli - which is true but it doesn't address our concern that the manipulation may be targeting language processing upstream of semantic processing (a parallel example for images: it's like blurring the image before semantic processing takes place, in which case we would also expect the involvement of domain-general fronto-parietal areas to cope with increased task difficulty). If the authors are committed to a theoretical view that semantic and linguistic processing are inseparable, they should be very explicit about their theoretical commitments and outline their implications for result interpretation. Alternatively, they should clarify that their manipulation may target language processing more generally rather than only semantic control.
The expected response pattern for the semantic control network (SCN) and multiple-demand network (MDN) across the experimental conditions (semantic/perceptual × high/low). For example, in the reply, the authors mentioned that semantic control was manipulated by high vs. low stimulation. However, in Figure 5, we see stronger overlap with the SCN for the Sem > Perc map rather than the High > Low map. The authors should clarify more clearly the distinction between the left and right panels of Figure 5A and explain what each result is intended to demonstrate. For example, because the paradigm was adjusted for difficulty, we may not expect to observe a traditional high > low difficulty effect in the perceptual conditions within the MD system. Domain-general regions would instead be reflected by comparable responses to semantic and perceptual conditions, whereas more semantic-selective regions would show stronger responses to semantic conditions, as shown in Figure 5.
Relatedly, the authors did not directly address the concern regarding task difficulty. The authors stated that "our difficulty manipulation was the TMS-induced modulation (high vs low stimulation intensity) during task processing while keeping task difficulty (stimulus presentation) constant." However, the behavioral results still show a significant task effect. The equivalence tests are conducted between high vs. low stimulation within each condition, but the main comparison also involves semantic vs. perceptual conditions.
Finally, we still have methodological concerns regarding the ROI analysis. The authors provided subject-specific fROI analyses, but if we understand correctly, they did not use independent data for ROI definition and response extraction. In addition, the fROIs appear to have been defined using an omnibus contrast across all conditions. In general, this analysis remains unclear to me. For example, it is not clear what contrast is being referred to in the following sentence: "For every subject and each MDN/SCN cluster, we sampled the localizer t-statistic image and first applied a voxel-wise threshold of $p < 0.001$, uncorrected, to remove weak or noisy responses (Nieto-Castañón and Fedorenko 2012)."

Minor points:
- The authors noted that, when running gPPI, the task regressor was not convolved with the HRF, which is not standard practice (McLaren et al., 2012).
- In the Discussion, the authors state: "In contrast, the right MFG and PMd were both upregulated across high stimulation intensity semantic and perceptual conditions, consistent with a role in domain-general difficulty and performance monitoring (Quillen et al. 2021)." However, if we are reading the figure correctly, there does not appear to be an intensity effect in the perceptual conditions for rMFG and rPMd.

Reviewer #4

(Remarks to the Author)
The authors have done an excellent job addressing the concerns raised in the initial review process. The manuscript is substantially improved in clarity, rigor, and interpretation, and overall I found this to be a technically sophisticated and conceptually important study. The use of interleaved TMS-fMRI to probe acute network adaptation during semantic processing is especially compelling, and the manuscript now provides a much clearer account of how semantic and domain-general systems interact under perturbation.

The revision thoughtfully addresses many of the key methodological and interpretational concerns from the previous round of review. In particular, I appreciated the expanded justification of the control conditions, the clearer distinction between planned and exploratory analyses, and the more balanced interpretation of the "compensation" findings. The additional statistical reporting (e.g., bootstrap confidence intervals, leave-one-out analyses) also strengthens confidence in the reported effects.

The manuscript now does a much better job situating the findings within broader models of semantic control and multiple-demand network engagement. I also thought the new discussion emphasizing a shift from "where" to "how" in semantic network neuroscience was especially strong and nicely framed the broader significance of the work.

Overall, I think this is a strong and innovative manuscript that makes a valuable contribution to the literature on semantic cognition, adaptive network dynamics, and interleaved TMS-fMRI methodology. The authors responded carefully and professionally to the prior critiques, and I believe the manuscript is now suitable for publication pending minor revisions:
1. I encourage the authors to continue using somewhat cautious language when referring to "compensation." The manuscript is much improved in this regard, but there are still a few places where the wording implies stronger causal evidence for compensation than the data fully support. In several instances, phrasing such as "putatively compensatory" or "associated with preserved performance" may better match the level of inference supported by the data.
2. The distinction between semantic match and mismatch trials is now better justified, particularly with the clarification that this decomposition was motivated by pilot data. I think the manuscript would benefit from a brief conceptual discussion of why matches and mismatches may recruit partially different control mechanisms or processing strategies.

3. The gPPI connectivity findings are interesting and potentially important. I would just encourage slightly cautious wording regarding network “reconfiguration” or mechanistic interpretation of connectivity directionality, given the limitations of gPPI analyses.

Reviewer #5

(Remarks to the Author)
I co-reviewed this manuscript with one of the reviewers who provided the listed reports. This is part of the Nature Communications initiative to facilitate training in peer review and to provide appropriate recognition for Early Career Researchers who co-review manuscripts.

Open Access This Peer Review File is licensed under a Creative Commons Attribution 4.0 International License, which permits use, sharing, adaptation, distribution and reproduction in any medium or format, as long as you give appropriate credit to the original author(s) and the source, provide a link to the Creative Commons license, and indicate if changes were made.
In cases where reviewers are anonymous, credit should be given to 'Anonymous Referee' and the source.
The images or other third party material in this Peer Review File are included in the article’s Creative Commons license, unless indicated otherwise in a credit line to the material. If material is not included in the article’s Creative Commons license and your intended use is not permitted by statutory regulation or exceeds the permitted use, you will need to obtain permission directly from the copyright holder.
To view a copy of this license, visit https://creativecommons.org/licenses/by/4.0/

<u>Response Letter</u>

We thank the editor and five anonymous reviewers for their thoughtful and constructive comments, which have helped us to improve the clarity and rigor of the manuscript. Please find below our point-by-point replies to all suggestions. Replies and changes made to the manuscript are marked by *Italic* fonts in this letter and highlighted in red in the manuscript.

<u>Reviewer #1</u>

This is a really important work in the understanding of language cortical networks and the role of each area. Especially for rehabilitation and presurgical mapping this work is important to navigate clinicians in investigating this area as well with various tasks.

Since, this team has unique expertise in applying TMS-fMRI my comments will be restricted in the manuscript itself and some minor methodological issues.

<u>Response:</u> We thank the reviewer for their positive evaluation of our manuscript and their constructive feedback that helped us to improve our manuscript. We have addressed all your points as detailed below.

Reviewer Comment 1:

You used as high intensity the 100%MT and low 40%. Did you try to get a threshold value to apply TMS. It is known that the TMS threshold in below the MT. Did you try to define a threshold?

Why did you use the MT value? This value is usually applied in speech cortical mapping (Krieg et al. 2017; Lioumis et al. 2012) but it can be reduced also substantially (up to 70%).

The control value is 40% which is actually a very good choice because it serves as both control and sham in my opinion.

<u>Response:</u> Thank you for this comment. Indeed, it is common practice to apply a 100% vs 40% setup in interleaved TMS-fMRI studies of cognition (Schuler and Hartwigsen 2025, BrainStim; Woolgar et al. 2026, NatProt). Several studies that have directly compared functional thresholds vs motor thresholds suggest higher thresholds in association cortices (for review see Schuler et al. 2025, NBBR). Therefore, and in the absence of a validated dose-response readout for our specific task, we chose 100% stimulation intensity to obtain stable stimulation effects in the TMS-fMRI environment. This choice is further supported by our previous interleaved TMS-fMRI study over left DLPFC that has shown largest BOLD effects at stimulation intensities of 100% MT (as compared to 80, 90 and 110%).

We have added the following explanation to the Methods section (page 29):

"We chose a stimulation threshold of 100% of the individual rMT because a validated functional threshold for our specific task was not yet available (Schuler et al. 2025). Our choice was further supported by a previous study demonstrating stronger neural responses to stimulation in association cortex at 100% rMT compared with both lower and higher intensities (Tik et al. 2023)."

Reviewer Comment 2:
Can you talk a bit more about the timing of TMS delivery. Did this change across tasks? could it be that this playes a role in your findings? Even if this is a case this is a very important finding for both network validation and presurgical evaluation, since many times neurosurgeons do not take into consideration these very important details, and they apply unsynchronised currents during awake craniotomies resulting in very variable language-induced errors or lack of errors.

Response: *Thank you for this very important question. We intentionally adjusted the stimulation timing to the semantic condition only since this was the task of interest in our study. We expected visual-spatial attention to be processed at a different time course (~0-200 ms). This allowed us to directly probe the specificity of the stimulation effect optimized for semantic processing. Moreover, we expected spatial specificity for the semantic task since early visual-spatial processing should engage different brain regions including more pronounced right-lateralization.*

We have added the following explanation to the Methods section (page 29):

"We chose this delay since it guaranteed stimulation during different stages of semantic processing (200-600 msec) without affecting early visual-spatial processing (0-200 msec; Capotosto et al. 2009; Enge et al. 2023; Schendan and Ganis 2015). Please note that we calibrated the timing for the most effective semantic disruption in a pilot experiment (see SI Pilot Experiments)."

Reviewer Comment 3:
I think your work and your paradigm can be a great tool among other very few ones (the novel multi-locus TMS) to validate the current language network models. The propagation of your induced field is a very crucial tool for this purpose. In your discussion it will be important to generalize a bit, and explain how the TMS-fMRI can give further light on the how the whole network works. I think your approach can evaluate very well old and new models and categorize the functions for each area involved. This is a major paradigm shift and need to be explained in detail.

Response: *Thank you for hinting us towards this point. We have added a discussion on the potential of this paradigm to better understand spatio-temporal dynamics within the semantic network in health and disease at the network level (page 25):*

"Aside from providing insight into flexible adaptation in the semantic network, our findings demonstrate how combining TMS with fMRI enables a shift from static localization of cognitive functions ("where") toward a mechanistic account of network dynamics ("how") underlying semantic processing. Consistent with emerging non-modular frameworks (Hayden et al. 2025), this approach provides causal evidence for distributed and interactive semantic processing. By transiently perturbing a targeted node while measuring whole-brain activity, interleaved TMS-fMRI reveals how neural activity is redistributed across the semantic and support networks, identifying regions that exhibit reduced engagement, relative stability or compensatory upregulation. More broadly, integrating interleaved TMS-fMRI with emerging approaches such as multi-channel TMS (Souza et al. 2025) in future studies holds promise

for advancing a more fine-grained understanding of large-scale neural dynamics in semantic cognition."

Reviewer Comment 4:
Last you miss some very important references, although you include them in reviews, but for the readership would be important to have a chance to know them. 1) https://pubmed.ncbi.nlm.nih.gov/37390891/ which introduced how TMS-fMRI can be applied and 2) the virtual lesion demonstration https://pubmed.ncbi.nlm.nih.gov/2027485/ and the first virtual lesion approach for speech cortical mapping https://pubmed.ncbi.nlm.nih.gov/22108143/ .

Response: *Thank you for pointing us towards these publications. We have integrated them into our manuscript as follows:*

Introduction section (page 4): "Finally, online TMS studies have enabled focal, non-invasive causal mapping of cognitive functions, but do not allow for whole-brain mapping of direct stimulation effects yet (Lioumis et al. 2012; Pascual-Leone et al. 1991)."

Discussion section (page 21): "These findings converge with prior concurrent TMS-fMRI studies showing that remote activity increases are associated with performance gains in semantic memory tasks (e.g. Hawco et al. 2017; Hermiller et al. 2020; Vasileiadi et al. 2025)."

Reviewer #2
The study employs interleaved TMS-fMRI to investigate acute neural compensation during semantic processing, a technically advanced approach that provides real-time insights into network dynamics. This addresses a critical gap in understanding how the brain adapts immediately to perturbation, complementing prior offline TMS or static neuroimaging studies.By targeting the left posterior middle temporal gyrus (pMTG)–a core semantic control region–the authors demonstrate task-specific recruitment of both local semantic networks (bilateral IFG, SMA) and remote domain-general regions (right middle frontal gyrus [rMFG], superior parietal lobule [SPL]). The dissociation between local disruption (slower reaction times with increased pMTG activity) and remote compensation (less behavioral disruption with rMFG upregulation) is compelling.

Response: *We thank the reviewer for their overall positive evaluation of our manuscript. We have addressed all your points carefully and feel that the manuscript has strongly profited from your suggestions.*

Major Concerns

Reviewer Comment 1a:
Stimulation Specificity and Control Conditions
TMS Intensity Validation: The low-intensity condition (40% rMT) is intended as a sham control, but the authors should clarify whether this intensity truly avoids neural modulation. Prior work suggests subthreshold TMS can still induce subtle effects, raising questions about whether "low" intensity might confound interpretations of "high" intensity effects.

<u>Response:</u> *We thank the reviewer for this comment. Indeed, it is common practice to apply a 100% / 40% setup in interleaved TMS-fMRI studies of cognition (Schuler and Hartwigsen, 2025, BrainStim; Woolgar et al. 2026, NatProt). This has proven useful in mimicking peripheral TMS side effects. Since the induced electromagnetic field rapidly declines as a factor of intensity, we do not expect that 40% stimulation intensity would have a direct effect on the cerebral cortex. Effective subthreshold protocols still use intensities of 80% MT, which is far above our chosen TMS control intensity (Klomjai, Katz & Lackmy-Vallée 2015, Annals of Physical and Rehabilitation Medicine). Moreover, the task control condition (semantic vs perceptual task/rest under high intensity) allowed further control for task specificity under the same stimulation intensity condition. Reviewer #4 encouraged us to better highlight the study design and its power to efficiently control side effects in the MR-environment. Therefore, we have added the following paragraphs to the Methods and Results sections to address the topic of control conditions:*

Methods section (page 28): "Control Conditions
TMS has several side effects, including auditory and somatosensory activity as well as muscle twitches and attentional shifts, that necessitate rigorous control conditions. Addressing these confounds is particularly challenging in TMS-fMRI settings, where MR-compatible sham coils are unavailable and peripheral stimulation can substantially influence the BOLD signal, complicating the dissociation of transcranial and non-transcranial effects. To mitigate these limitations, we implemented a multi-level control framework in line with recent recommendations (Schuler and Hartwigsen 2025; Woolgar et al. 2025). Specifically, we contrasted (i) a task of interest with a control task (task specificity: semantic vs perceptual), (ii) task engagement with a rest state (state specificity: semantic vs rest), and (iii) effective vs subthreshold stimulation intensities (side effects control: 100% vs 40% rMT). While the latter controls for auditory and somatosensory confounds, it does not fully dissociate intensity-dependent effects. The integration of these complementary control conditions provides a robust basis for isolating transcranial contributions and offers a refined characterization of TMS-induced effects (see Figure 1B)."

Results section (page 6-8): "This design allowed controlling for task, state and general side effects in the challenging TMS-fMRI environment (cf. Figure 1B and Methods section for details)."

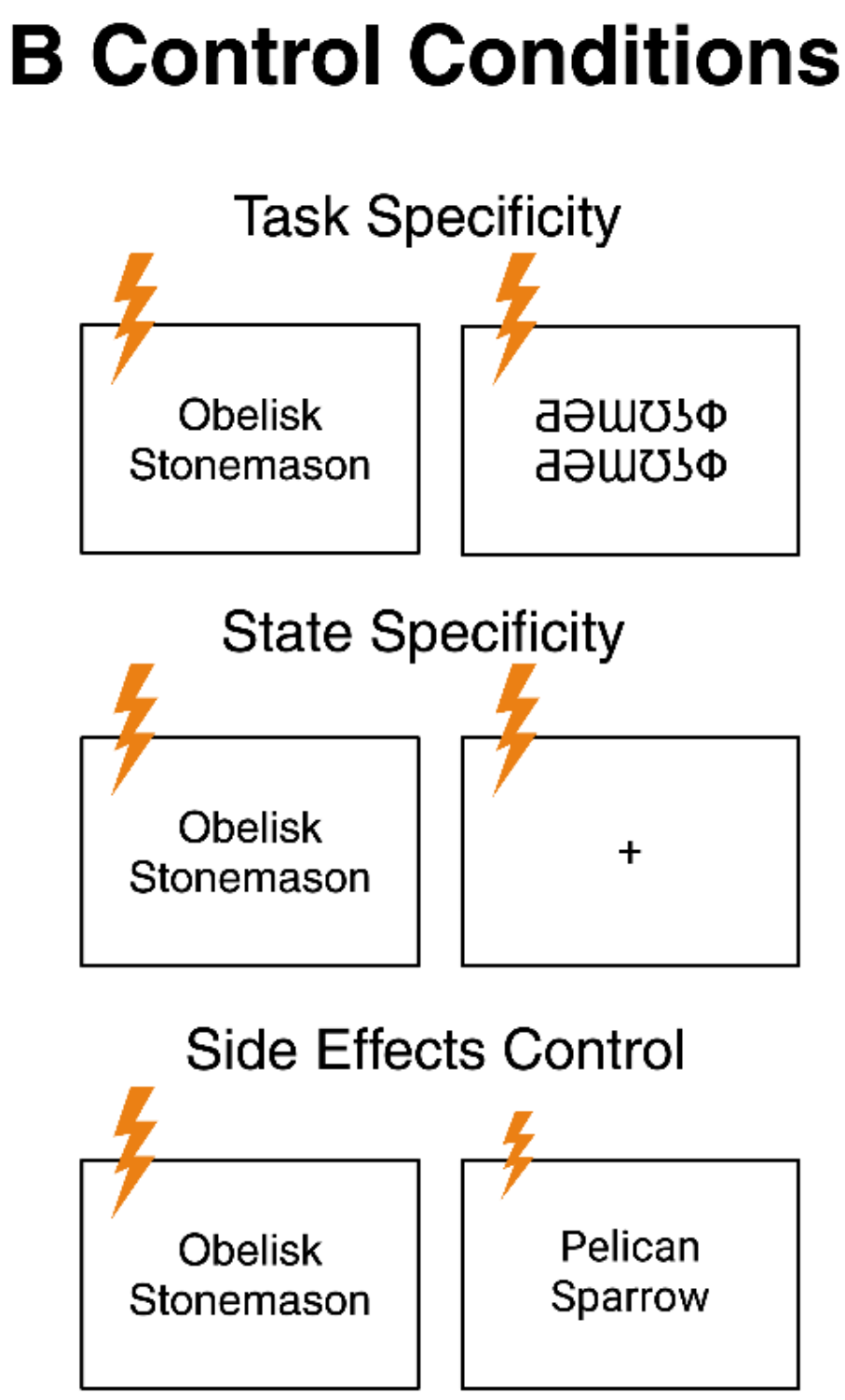

*[...] **B.** Control Conditions. Task Specificity. Stimulation during a control task at the same intensity (high stimulation intensity: 100% rMT). State Specificity. Stimulation during a control state (rest) at high intensity (100% rMT). Side Effects Control. Control for general auditory and somatosensory effects at 100% rMT (high) vs 40% rMT (low) stimulation intensity. [...]*

Discussion section (page 23): "Combining different control conditions (task-, state-specificity and general side effects control), we were able to dissociate unspecific side effects evoked by arousal, startle responses, or peripheral effects from compensatory mechanisms in response to TMS."

Reviewer Comment 1b:
Perceptual Task Specificity: The perceptual control task (inverted symbols) may not fully match the semantic task's cognitive load. The authors note higher accuracy in perceptual conditions (95% vs. 85% for semantic), indicating potential differences in difficulty. This could explain why domain-general regions (e.g., rMFG) are less engaged in perceptual tasks, rather than reflecting semantic-specific compensation.

<u>Response:</u> We agree with the reviewer that the perceptual task might have been easier. However, as depicted in our ROI analysis, contrast estimates in rMFG and rPMd are higher for both perceptual conditions compared to the semantic low-intensity condition but comparable to the semantic high-intensity condition, thus clearly indicating lateralized selectivity of domain-general areas for the perceptual task. The whole-brain correlation analyses were calculated between difference scores separately for semantic and perceptual conditions and should therefore not be biased by any difficulty differences. Whole-brain contrasts actually reflect stronger engagement of semantic and MDN regions for the semantic task, which is in line with our hypothesis for semantic compared to perceptual processing. Moreover, the baseline data show that, in the absence of TMS, the comparison of the semantic vs perceptual task resulted in circumscribed activation changes in the left IFG and pMTG

without any above-threshold activation changes in MDN regions. Consequently, we are confident that the observed compensatory effects were task specific.

Reviewer Comment 2a:
Statistical and Analytical Rigor
Multiple Comparisons: Whole-brain analyses use cluster-level FWE correction, but ROI analyses (e.g., MDN regions) rely on Bonferroni correction without pre-registration of ROIs. The overlap between a priori semantic control regions and exploratory whole-brain results (e.g., rMFG) should be explicitly justified to avoid circularity.

Response: *We thank the reviewer for raising this important point. Our primary objective was to characterize whole-brain changes in neural activity and their relationship with task performance. Guided by this aim, we had two a priori hypotheses: (i) disruption of semantic processing would induce local perturbations, reflected in activity changes and performance decrements; and (ii) remote activity increases, particularly outside the core semantic network, would reflect compensatory mechanisms.*

Accordingly, our analytical approach focused on whole-brain effects complemented by theory-driven regions of interest (ROIs). We have clarified this rationale more explicitly throughout the manuscript. Following the evaluation of these pre-planned analyses, we conducted additional exploratory ROI analyses to further probe the mechanisms underlying potential compensatory responses. These analyses are now clearly delineated from the pre-planned analyses throughout the manuscript.

We have made the following additions to the Methods section:

Methods section (page 32): "For in-depth exploration of the impact of TMS on the recruitment of domain-general areas of the multiple demand network, we extracted contrast estimates for the four regressors semantic high, semantic low, perceptual high and perceptual low from 7 key regions of the multiple demand network, including bilateral anterior insula (antIns), supplementary motor area (SMA), bilateral dorsal premotor cortex (PMd) and bilateral middle frontal gyrus (MFG) based on a canonical MDN reference map (Fedorenko et al. 2013; https://imaging.mrc-cbu.cam.ac.uk/imaging/MDsystem)."

Methods section (page 33): "This was a planned decomposition analysis, based on differences in behavioral trends from our TMS pilot. Since we restricted our analysis to a small family (n=2) of planned, hypotheses-driven comparisons, we did not perform multiple comparison correction for whole-brain regression models and correlation analyses at the stimulation site, consistent with recommendations by Rothman (1990) and recent complementary suggestions (Boulesteix and Hoffmann 2024; García-Pérez 2023; Hooper 2025)."

Methods section (page 33/34): "Finally, for exploratory analyses, we extracted low intensity-normalized beta-estimates from interaction-derived (left SMG, right PrG, right SPL, right SFG) cluster-peaks of statistical maps (6 mm spherical ROIs) and correlated these with reaction times for correctly solved items (response efficiency). Exploratory analyses were FDR-corrected for multiple comparisons (semantic matches, semantic mismatches)."

We have added these clarifications to the Results section:

Results section (page 12): "This was a planned decomposition since behavioral piloting data hinted towards qualitatively different processing strategies for both trial types."

Results section (page 12): "Correlations for semantic mismatches did not reveal any significant effects (p > 0.05)."

Results section (page 13): "No voxels survived cluster-level FWE-correction for the semantic matches regression analysis."

Results section (page 16): "Stimulation-induced increases in rPrG were associated with a trend for less stimulation-induced delays in semantic reaction times during semantic match trials (r = -0.414, p = 0.080) but there was no significant correlation for semantic mismatch trials. There was no significant correlation between activity changes in the left SMG and reaction times for any of the conditions." We have updated Figure 4A (page 15) accordingly.

Results section (page 16/17): "Indeed, a negative correlation with semantic reaction times revealed that stimulation-induced increases in activity in the rSFG were associated with less stimulation-induced disruptions in reaction times for semantic mismatches (r = -0.450, p = 0.048, FDR-corrected, 95% bootstrap C.I. [-0.724 -0.117]). No significant effect was found for semantic matches in the rSFG. Finally, for rSPL, there was a trend towards a negative correlation between semantic reaction times and activity changes for semantic matches (r = -0.433, p = 0.062, FDR-corrected) but no significant correlation for semantic mismatches."

Results section (page 17): "To further elucidate compensatory activity shifts towards domain-general control regions during semantic perturbation in the left pMTG, we performed exploratory participant-independent region-of-interest (ROI) analyses in the multiple demand network (MDN; Figure 5B)."

Reviewer Comment 2b:

E-Field Simulations: While E-field magnitudes confirm pMTG targeting, the relationship between E-field strength and behavioral effects (e.g., slower reaction times in posterior pMTG for mismatches) is only briefly mentioned. A more detailed analysis of E-field-behavior correlations across the stimulated region would strengthen causal claims.

Response: *We thank the reviewer for this valuable suggestion. We have added further information on E-field-behavior correlations and updated SI Figure 4. It now shows correlation coefficients from zero.*

We have made the following changes to the manuscript:

SI Methods section (page 43): "Exploratory Electric Field-Behavior Correlations
To explore the effects of electric field strength on behavioral modulations, we calculated correlation coefficients (r) for low intensity-normalized reaction times (response efficiency for semantic matches and semantic mismatches) and electric field magnitude inside the >40 V/m-thresholded mask."

SI Results section (page 48/49): "Exploratory Electric Field-Behavior Correlations
Exploratory electric field (E-field) correlations in the broader stimulated area across participants hinted towards more posterior stimulation being associated with delayed reaction times during mismatch trials. Conversely, faster reaction times were associated with higher E-field magnitude around the posterior opercular area during semantic match trials (SI Figure 4).

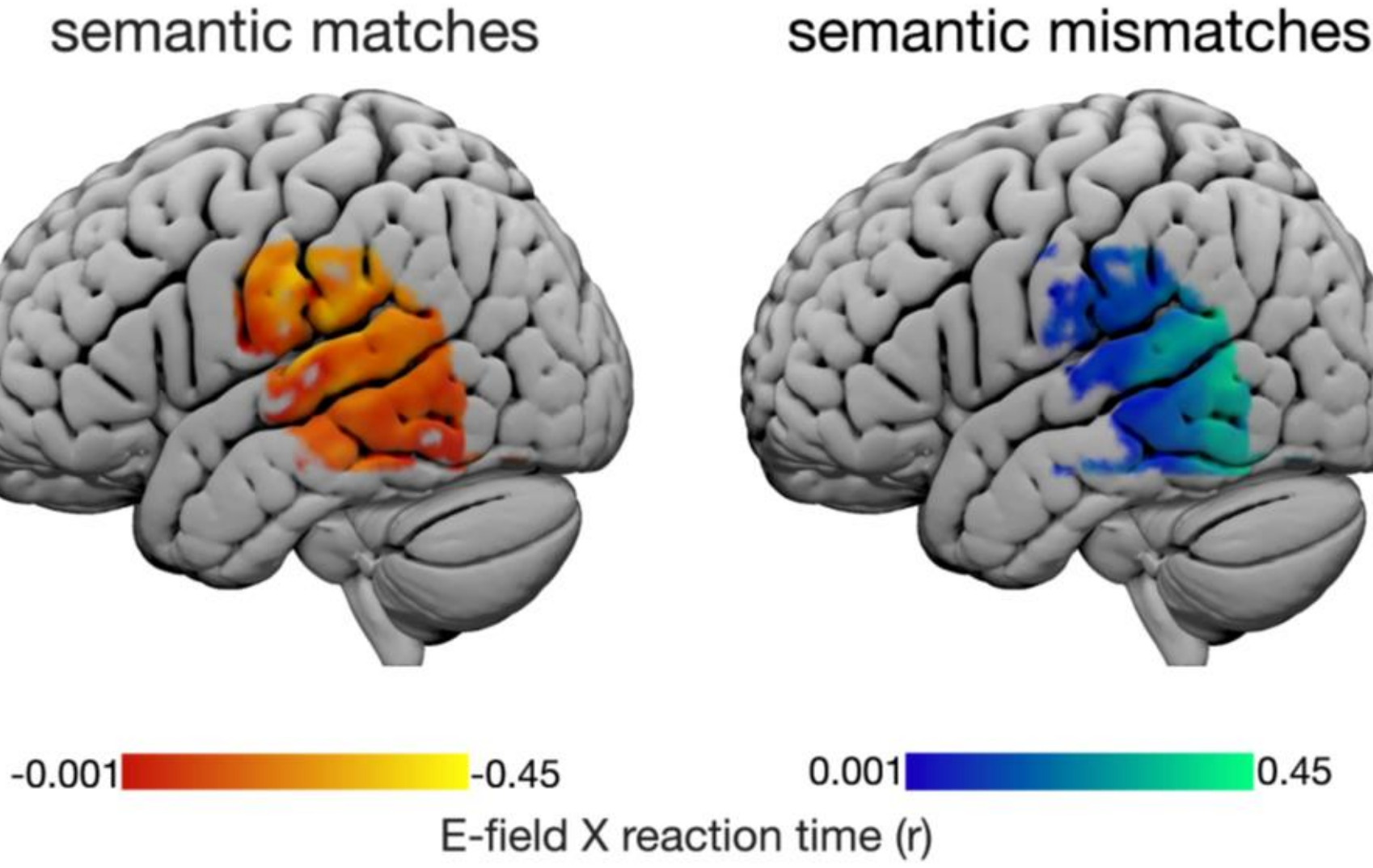

SI Figure 4. Correlations between reaction times and electric field (E-field). *Increased E-field magnitude was moderately correlated with increased reaction times in the more posterior part of the pMTG for semantic mismatches. Increased E-field magnitude was moderately correlated with decreased reaction times in the posterior opercular regions for semantic matches. Note: No significance testing applied."*

Discussion section (page 20): "Moreover, semantic reaction time delays were stronger with increased electric field strengths in the more posterior portion of the pMTG for semantic mismatches, supporting local TMS effects on semantic control as reported previously (Davey et al. 2015; Gao et al. 2022; Lambon Ralph et al. 2017)."

Reviewer Comment 3a:
Interpretation of Compensatory Mechanisms
Domain-General vs. Semantic Network Interactions: The authors argue that rMFG upregulation reflects "compensation," but this could also reflect task-general effort (e.g., increased attention). Control analyses contrasting semantic vs. non-semantic high-load tasks (e.g., working memory) would clarify whether effects are truly semantic-specific.

<u>Response:</u> *We thank the reviewer for raising this point. We characterized the brain-behavior relationship as "less disruptive", which may indeed plausibly reflect increased attentional engagement in some participants. This interpretation would be consistent with our assumption that, in the absence of within-network compensation, domain-general systems support task performance. Such support likely involves networks associated with attention, working memory, and cognitive control.*

To clarify this, we added the following sentence to the Introduction section (page 3):

"The MDN is central to executive processing and supports functions such as task set maintenance, attentional allocation, and response conflict resolution."

Regarding your second point, we focused on the direct comparison between semantic conditions for high- and low-intensity stimulation to isolate the interaction between semantic processing and TMS. Following the reviewer's suggestion, we additionally conducted exploratory analyses between semantic and perceptual conditions under high-intensity stimulation. At the whole-brain level, these analyses revealed associations between activity increases in the left SPL and increased response speed in the match condition. In contrast, increased activity in the left superior temporal gyrus was associated with faster response speed for the mismatch condition. However, these effects did not survive multiple comparison corrections. In line with requests for conciseness, these results are reported in the Supplement.

We have made the following changes to the manuscript:

Methods section (page 34): "Supplementary regression results for the semantic high vs perceptual high stimulation intensity conditions can be found in SI Figure 6."

SI section (page 49/50): "The regression analysis for semantic vs perceptual matches under high stimulation intensity revealed an association between increased activity in the left SPL and increasing reaction times (positive relationship). In contrast, increased activity in the left superior temporal gyrus for semantic vs perceptual mismatches was associated with decreased reaction times (negative relationship; SI Figure 6). However, none of the comparisons survived multiple comparison corrections.

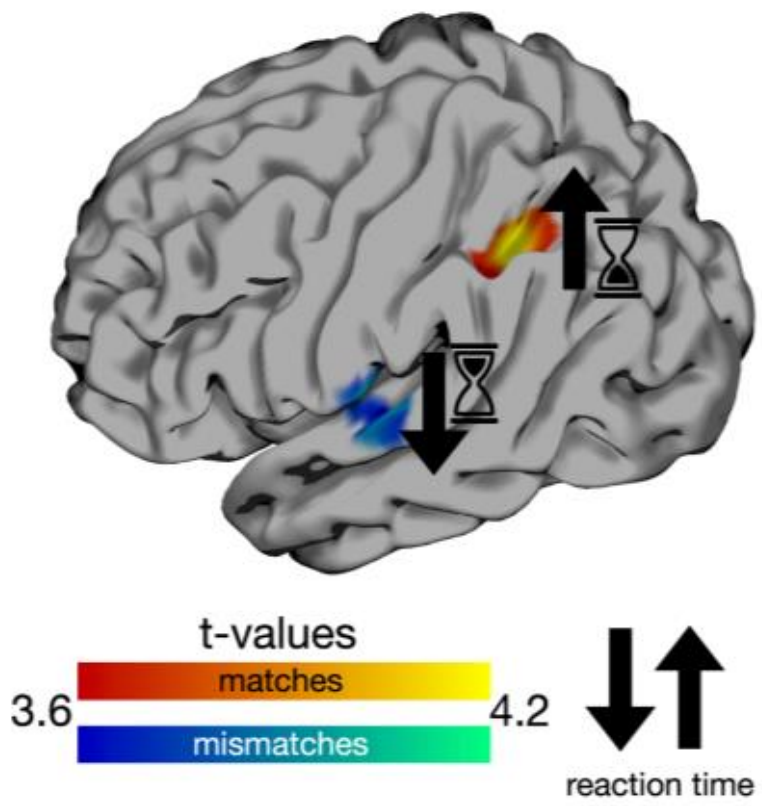

SI Figure 6. Brain-behavior relationships (regression for the comparison between tasks at high stimulation intensity and behavior). *Activity increases for semantic vs perceptual matches under high stimulation intensity in the left SPL are associated with increased reaction times (positive relationship). Activity increases in the left superior temporal gyrus for semantic relative to perceptual mismatches under high stimulation intensity are associated with decreased reaction times (negative relationship; n = 25; p < 0.001 uncorrected; voxel threshold k = 50). Note that these effects do not survive whole-brain correction for multiple comparisons."*

Reviewer Comment 3b:
State-Dependence: The interaction between TMS intensity and brain state (semantic task vs. rest) shows rSPL and rSFG effects, but the functional relevance of these regions to semantic processing is unclear. Linking these to existing models of semantic control would strengthen interpretations.

<u>Response:</u> We thank the reviewer for this suggestion. We have now elaborated on the interpretation of these results in light of semantic control. Please note that this attempt was merged with an additional suggestion by Reviewer #4.

We have made the following changes to the manuscript:

Results section (page 17): "Consistent with models of semantic control emphasizing the flexible engagement of distributed fronto-parietal regions (Hallam et al. 2016; He et al. 2025), putative compensation effects were thus state-dependent and qualitatively different at rest."

Discussion section (page 22/23): "Collectively, our findings highlight state-dependent interaction effects of online TMS (Jung et al. 2016; Kaiser et al. 2025): stimulation during semantic processing relative to a perceptual control task revealed task-specific activity increases in the SMG, whereas the comparison of the semantic task state with rest indicated a shift toward right-hemisphere regions associated with cognitive control. The latter effect is consistent with increased recruitment of right-hemispheric fronto-parietal regions in semantically challenging situations (Hallam et al. 2016; He et al. 2025). These observations provide novel insights into both the mechanisms of TMS and the dynamic allocation of cognitive resources."

Reviewer Comment 4:
Despite low spatial overlap between the semantic network and Multiple-Demand Network (MDN; Dice coefficients 0.0247-0.0726), ROI analyses show MDN activity increases.

<u>Response:</u> We thank the reviewer for raising this point. While the dice calculations are based on our piloting fMRI localizer data, the ROI analyses depict intended TMS effects on the task. We deliberately chose a task that should not result in domain-general activity changes without TMS, which was validated in the pilot fMRI-experiment. We then aimed at identifying TMS-induced increases in the recruitment of domain-general brain areas during semantic processing.

We have clarified our motivation in the Methods section (page 26):

"We chose this paradigm because it was specifically designed to minimize contamination of fMRI measures of semantic processing by activity changes driven by task difficulty. This allowed us to map TMS-induced increases in the recruitment of domain-general brain areas during semantic processing."

Reviewer Comment 5:
The impact of multiple comparison correction on small effect sizes needs explicit discussion. Some correlation analyses have a small sample size (n=25), e.g., the correlation between rSFG activity and reaction times in semantic mismatch trials (r=-0.450, p=0.024). Effect size reporting or bootstrap validation is advised.

Response: *We thank the reviewer for this suggestion. We have now clarified the descriptions on multiple comparison considerations (also referring to your Comment 2a). Following your suggestion, we have included bootstrapping intervals (after 5000 permutations) and additionally performed a leave-one-out analysis as suggested by Reviewer #4 for significant correlation analyses. Across analytical approaches, the results converged in supporting stable effects despite the small sample size (see SI Table 2). Bootstrapped confidence intervals are now reported alongside correlation coefficients in the main Results section.*

We have added the following to the Methods section (page 34):

"To guarantee robustness of results, we bootstrapped significant correlations for a 95% confidence interval (C.I.) running 5000 permutations per correlation. Bootstrapping intervals are displayed in SI Figure 7. Additionally, we calculated leave-one-out standard deviations (SD; SI Table 2)."

Furthermore, we have added the following passage to the SI Results section (page 50):

"Bootstrapping Results for ROI-Behavior Correlations

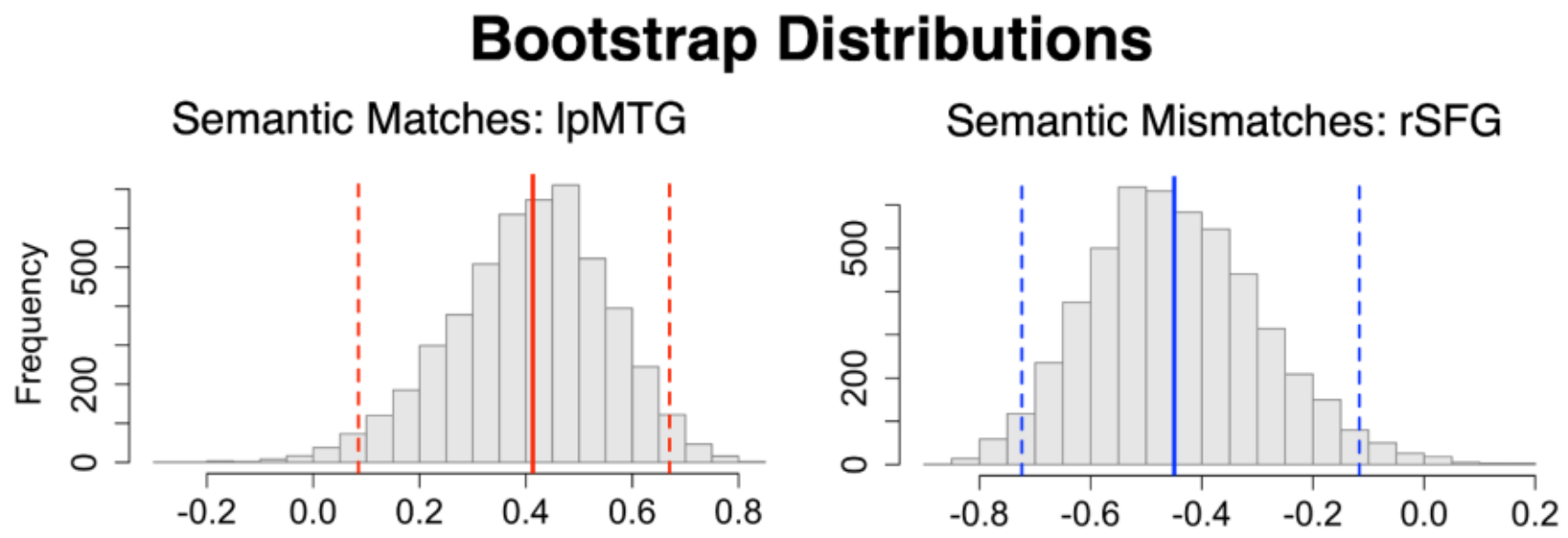

SI Figure 7. Bootstrap distributions for significant brain-behavior correlations in selected regions-of-interest for semantic matches and mismatches. *Left: moderate positive correlation between reaction times and activity strength (betas) in the lpMTG (stimulation target site), right: moderate negative correlation between reaction times and activity strength (betas) in the rSFG.*

SI Table 2. Leave-one-out and bootstrapping correlation results.

ROI	*Condition*	*loo mean*	*loo SD*	*r BS*	*CI low*	*CI high*	*r*
	Semantic Matches						
lpMTG (stim sites)		*0.413*	*0.032*	*0.413*	*0.085*	*0.67*	*0.413*
	Semantic Mismatches						
rSFG (26 30 46)		*-0.45*	*0.036*	*-0.45*	*-0.724*	*-0.117*	*-0.45*

ROI=region of interest, lpMTG=left posterior middle temporal gyrus, rSFG=right superior frontal gyrus, loo=leave-one-out, SD=standard deviation, r=correlation, BS=bootstrap, CI=confidence interval"

Minor Revisions

Reviewer Comment 6:
Clarify Stimulus Design: The adaptive staircase procedure for German word pairs is well-described, but details on how "semantic high/low" and "perceptual high/low" load were operationalized (e.g., difficulty levels, Levenshtein distance) should be expanded in the main text.

<u>Response:</u> We apologize for the confusion: "high" and "low" always refer to stimulation intensity (high: 100% rMT; low: 40% rMT). We have revised the manuscript to solve all previously ambiguous points and clarified this at first mention (page 5/6 of the Results section): "During this session, participants performed one run of the semantic paradigm with either high stimulation intensity (100% of resting motor threshold as effective stimulation, ***hereafter "high"****) or low stimulation intensity (40% of resting motor threshold as control intensity,* ***hereafter "low"****) being applied trial-wise, one run of the task without TMS (no TMS baseline) and one run of TMS without task (no Task; see Materials & Methods for further details)."*

Reviewer Comment 7:
Visualization of Brain-Behavior Correlations: Scatter plots (e.g., Figure 2C) would benefit from individual data points and 95% confidence intervals to illustrate variability in the relationship between rMFG activity and reaction times.

<u>Response:</u> We thank the reviewer for this suggestion. We have now updated all scatter plots based on your suggestions. Below is an example for Figure 2B,C for your reference.

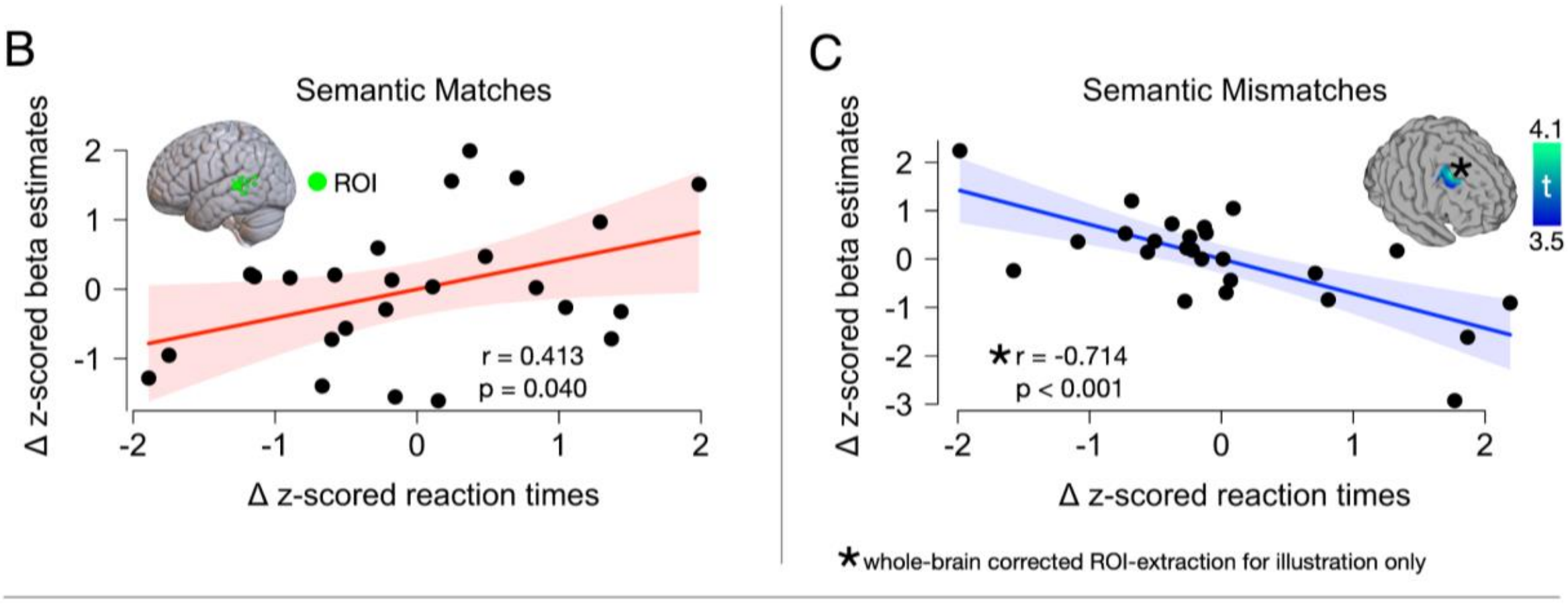

<u>Reviewer #3:</u>
The goal and design are clear, and the use of simultaneous TMS and fMRI is innovative. However, we have substantial reservation about the study's analyses and interpretation of the results.

<u>Response:</u> We thank the reviewers for their constructive feedback. Your suggestions have substantially improved the interpretation of our results. As acknowledged by all reviewers, this is the first study to investigate the acute neural correlates of flexible reorganization of neural networks during semantic processing. Our design choices and analysis plans were informed by a broad body of prior work and by extensive pilot testing, which we have further clarified in the revised manuscript. We hope that we have addressed all concerns as detailed below. Please note that, following reviewer #4's advice, we have included additional task-based connectivity analyses to our manuscript which further support our interpretation.

Major points

Reviewer Comment 1a:
Lack of distinction between language and semantic control. The authors frame their study as a study of semantic control areas; however, the semantic condition uses words whereas the perceptual condition uses foreign alphabet strings, meaning that the contrast includes language areas - many of the areas identified by the contrast might have been identified even during passive viewing of these stimuli. Moreover, the stimulation target is defined using that same contrast within pMTG, a region that contains swaths of language-selective contrast, so it's very possible that it's a language area, not a semantic control area that's being stimulated. Further, even if the target itself was a semantic control area and not a purely language area, I am not sure that TMS is spatially precise enough to only stimulate one and not the other, given the functional heterogeneity of pMTG. The authors state that their findings have implications for aphasia, which is a disorder of language.
So do they make claims about the language regions or semantic control regions?
To clarify why this distinction matters: language areas would be engaged during language comprehension/production whether or not there is a semantically demanding task; in contrast, semantic control areas would be preferentially recruited during semantically challenging tasks but wouldn't show a modality specificity, responding to both linguistic and non-linguistic semantically meaningful stimuli. So, the question of which network is being targeted would have important implications for both understanding the brain and developing strategies for treating aphasia, but it is not addressed here.

<u>Response:</u> We thank the reviewer for raising this point. Our task requires the active processing of semantic information and was designed for this purpose (Wilson et al. 2018, HBM). In practice, healthy young adult native speakers will always automatically process the meaning if two words are presented visually or auditorily. This also holds true for picture naming, a paradigm where even in neurological patient populations, areas for semantic processing become active in the absence of any linguistic stimuli (letters; Salmelin et al. 1994, Nature; Price et al. 2005, HBM; Benjamin et al. 2017, 2018, HBM). Finally, we made sure that participants were actively processing the task by employing a forced choice paradigm. We agree with the reviewer that a brain region might have different roles based on the context of the specific task. Design considerations were based on established studies showing TMS-effects on semantic processing and semantic control by modulating the pMTG (e.g., Davey et

al. 2015, JNeurosci; Lambon Ralph et al. 2017, NatRevNeurosci). Aside from task comparisons between semantic and perceptual decisions, we contrasted semantic processing with varying external difficulty manipulations (high vs low intensity stimulation).

We fully agree with the reviewer that these points were not made explicit enough in the manuscript and have made the following changes:

We adjusted our wording where misleading, e.g. "recovery from semantic aphasia" (page 25).

We clarify that we were interested in both general semantic processing and semantic control and specify their involvement throughout the manuscript, e.g. "Other work demonstrated that higher cognitive demands require flexible interactions between regions involved in general semantic processing, semantic control regions and domain-general areas of the multiple demand network (MDN; Nieberlein et al. 2024; Chiou et al. 2023)." (page 3); "Quantifying the impact of stimulation intensity on semantic decisions (the direct comparison of high and low stimulation intensity during semantic processing) revealed activity increases in both areas of the semantic network, including the stimulation site (lpMTG), and domain-general control areas (bilateral SMG, rMFG)." (page 8); "Activity increases were observed in the bilateral somatosensory cortices, right frontal lobe and extended into the left anterior temporal lobe and parietal cortex as general semantic regions." (page 21).

*We clarified that in this study, semantic control was not manipulated by the items but by the external perturbation of the brain state (Results section, page 5/6): "During this session, participants performed one run of the semantic paradigm with either high stimulation intensity (100% of resting motor threshold as effective stimulation, **hereafter "high"**) or low stimulation intensity (40% of resting motor threshold as control intensity, **hereafter "low"**) being applied trial-wise, one run of the task without TMS (no TMS baseline) and one run of TMS without task (no Task; see Materials & Methods for further details)."*

We added overlap maps for our main contrasts with established maps for the semantic control network (SCN) and the multiple demand network (MDN) (Figure 5A, Results section, page 18). For the contrast semantic high vs perceptual high stimulation intensity, the overlap was maximal for the semantic control mask (additional analyses are detailed below).

We have added the following to the Methods section (page 33): "For supplementary overlap maps between task contrasts (semantic high vs perceptual high, semantic high vs semantic low) and MDN and SCN (Figure 5A), we thresholded contrast images at $p < 0.001$ uncorrected with cluster-level FWE correction at $p < 0.05$, the MDN reference map was thresholded at $t = 1.5$, and the SCN reference map was thresholded at ALE = 0.02."

We have added the following to the Results section (page 17/18): "Finally, we created overlap maps between our main contrasts (semantic high vs perceptual high, semantic high vs semantic low) and the semantic control and multiple demand networks (SCN, MDN; Figure 5A). The semantic high vs perceptual high contrast overlapped with the SCN at the bilateral IFG, SMA and pMTG, and with the MDN at the left MFG, SPL and SMA. Furthermore, there was overlap with the MDN for the semantic high vs semantic low contrast in the right MFG and left SPL."

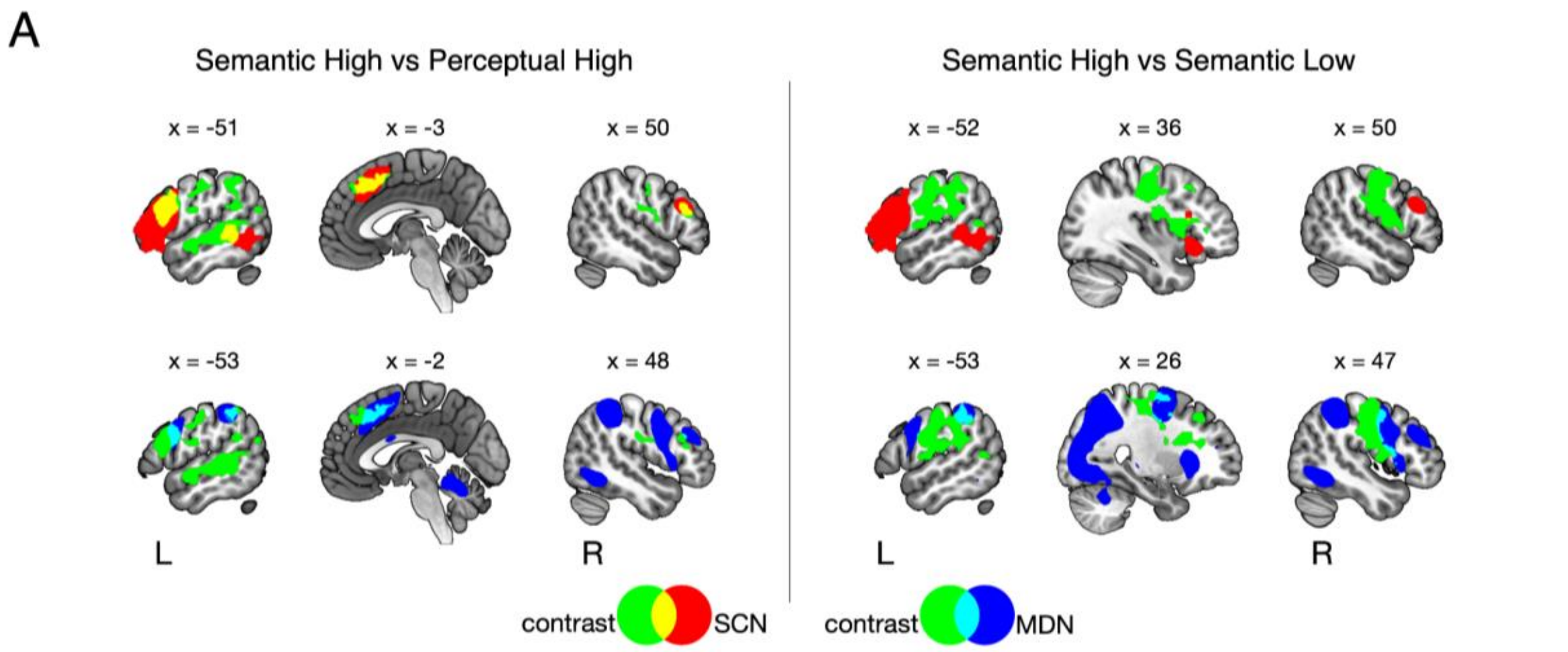

Figure 5. Stimulation-induced semantic activity increases in multiple demand and semantic control regions. A. *Overlap of task contrasts with MDN and SCN (semantic control network). The semantic high vs perceptual high contrast (green) overlapped with the SCN (red) in the bilateral IFG and left pMTG (yellow). Overlap with the MDN (blue) was found in the left MFG, SPL and SMA (turquoise). The semantic high vs semantic low contrast (green) overlapped with the MDN (blue) at the left SPL and right MFG (turquoise). MDN map obtained from Fedorenko et al. (2013), SCN map obtained from Jackson (2021). [...]*

Reviewer Comment 1b:

Lack of functional localization when describing the results. One of our main concerns is how regions are assigned to different functional networks. For instance, is right IFG a SC region, an MD region, or a language region? As noted in the first paragraph of the Results section, significant activation for Semantic_gt_Perceptual in the baseline condition does not include right IFG. While left IFG is consistent and reliably identified as a SC region, right IFG is smaller and MD activation is stronger on the right frontal cortex (Jackson et al. 2021). Similar concern also applies to SMA. In particular, SMA is mentioned as part of the core SC network in Figure 2, but later as one of the MD regions in Figure 4.

<u>Response:</u> *To address this issue, we have added an additional figure that shows clear overlap between canonical semantic control in the right IFG activation from our study (see Comment 1a and Figure 5A).*

The overlap of right IFG activation with the canonical semantic control network (SCN) relative to the MDN supports its association with the SCN. Additional subject-specific activity analyses within the right IFG mask (SCN) and adjacent right dorsolateral prefrontal cortex (DLPFC, MDN) revealed significantly greater IFG activation changes for semantic high vs perceptual high stimulation intensity conditions, consistent with our whole-brain results (see Comment 1c and Figure 2A). The right IFG was not engaged during the baseline condition above-threshold, and its increased recruitment likely reflects elevated cognitive demands induced by TMS-interference during task processing.

In line with the reviewer's comment, we consider the supplementary motor area (pre-SMA/SMA) as an interface between SCN and MDN (e.g. Chiou et al. 2023, CerebCortex). Additional participant-specific fROI-analyses using SCN- and MDN-defined SMA/pre-SMA masks showed strong MDN involvement alongside weaker but reliable semantic control engagement, consistent with our prior ROI analyses. We therefore interpret the SMA/pre-SMA

as a shared, non-dissociable interface between networks, reflecting the integrated organization of large-scale brain systems, and have incorporated this point into the Discussion section.

We have added the following passages to the manuscript:

Results section (page 8): "Given that the rIFG and SMA are functionally proximal to domain-general control regions, we conducted network-restricted, participant-specific functional ROI (fROI) analyses (see SI Methods, SI Figure 8; Figure 5A). These showed a predominant contribution of the multiple demand network (MDN) to remote stimulation effects in the SMA, alongside smaller but significant semantic control network (SCN) engagement. In contrast, the rIFG exhibited stronger SCN engagement."

Results section (page 19): "Consistent with its putative role as an interface between semantic and domain-general control, complementary subject-specific fROI analyses within SCN and MDN masks confirmed this pattern for the SMA, with a relatively stronger engagement of the MDN. Similarly, functionally restricted ROI analyses for the left middle frontal gyrus converged with participant-independent results (compare Figure 5B and SI Figure 8)."

Discussion section (page 21): "By contrast, upregulation of an adjacent rMFG subregion and its increased functional coupling with the pre-SMA—a hub at the interface of semantic and domain-general control—likely reflects compensatory network reconfiguration following transient disruption of semantic processing (Chiou et al. 2023)."

Reviewer Comment 1c:
Related, line 138 mentions that Semantic high_gt_Perceptual high activates domain-general regions. What are these regions? Figure 2A only denotes regions for the SC network. In short, currently it is unclear to me whether and to what extent there's selective engagement of the MD regions during the semantic condition after effective stimulation. Left MFG ad SMA perhaps? But I will be careful to conclude these are MD regions, given the decent of overlap between the MD and the SC network on the left hemisphere and in those anatomical regions (Jackson et al. 2021).

<u>Response:</u> *Thank you for pointing us towards this issue. Our initial manuscript already reported MDN engagement in frontal ROIs based on data-independent ROI selection. We found significant activation increases selectively for the semantically demanding condition (during high stimulation intensity TMS) relative to the semantically less demanding condition (during low stimulation intensity TMS). We have now added participant-specific fROI analyses restricted by SCN and MDN masks to show contributions of both network hubs in the context of higher task demand. As stated in the previous reply (Comment 1b), the involvement of the SMA was quantitatively stronger for MDN as compared to SCN. Similar effects were observed for the left MFG. Please also refer to Figure 5A.*

We have added the following text to the Supplement:

SI Methods (page 43/44): "Network-restricted fROI Analyses

We delineated subject-specific functional regions of interest (fROIs) using a top percentage procedure applied within canonical masks of the multiple demand network (MDN) and the semantic control network (SCN; Fedorenko et al. 2013).
For the MDN mask definition, we started from a previously published MDN template map and thresholded it at t > 1.5 (Fedorenko et al. 2013). The thresholded MDN mask and a thresholded metaanalytic SCN mask (Jackson, 2021) were each partitioned into spatially contiguous components using "26-connectivity" in three dimensions, yielding a set of anatomical-functional "search volumes" for each network. Since the canonical MDN mask resulted in very large clusters at t > 1.5, we created a second mask thresholded at t > 2. While this allowed for better cluster separation, it had the tradeoff of smaller ROIs. For this reason, our final comparisons were calculated for both thresholds.
Subject-specific functional localization was based on a conjunction contrast that combined all four experimental conditions (effects-of-interest contrast). For every subject and each MDN/SCN cluster, we sampled the localizer t-statistic image and first applied a voxel-wise threshold of p < 0.001, uncorrected, to remove weak or noisy responses (Nieto-Castañón and Fedorenko 2012). Within the surviving voxels of a given cluster, we then retained the top 10% of voxels with the highest t-values at the single-subject level. The resulting voxel sets constituted the subject-specific MDN and SCN fROIs for each cluster.
For each subject-specific fROI, we subsequently extracted condition-specific beta estimates from the first-level GLM. Beta coefficients corresponding to the four experimental conditions (semantic high, semantic low, perceptual high, perceptual low) were averaged across all voxels within an fROI, yielding one mean beta per subject, network (MDN vs SCN), cluster, and condition. These ROI-wise estimates were used for statistical comparisons and visualization of network-and cluster-specific response profiles."

SI Results (page 51): "Network-restricted fROI analyses

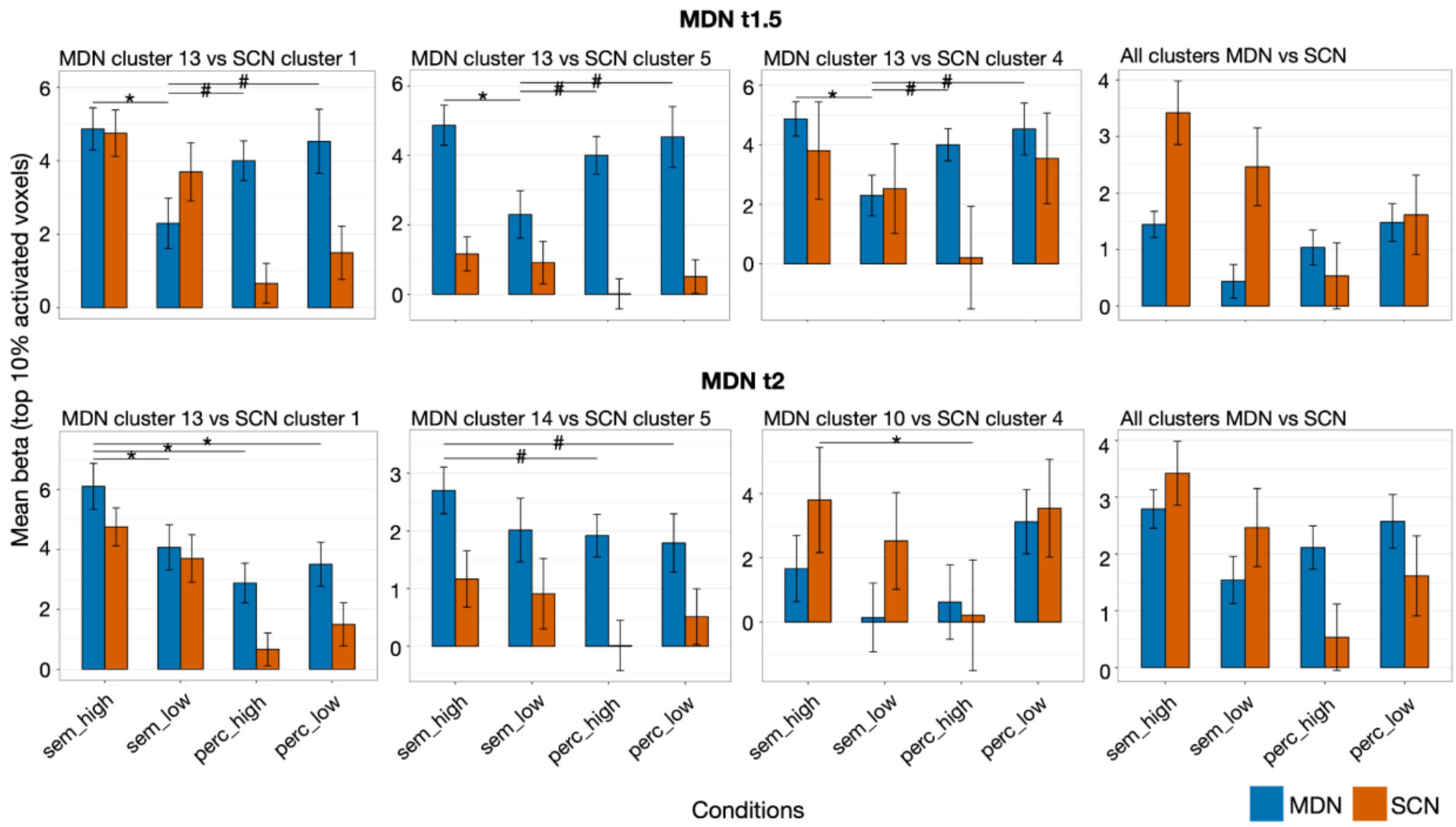

SI Figure 8. Subject-specific fROI-analyses for MDN and SCN overlap in selected clusters. *Upper panel: Comparison within MDN network-mask thresholded at t > 1.5. MDN cluster 13 = frontal cluster, SCN cluster 1 = left IFG, SCN cluster 4 = right IFG, SCN cluster 5 = SMA. Lower panel: Comparison within MDN network-mask thresholded at t > 2 (MDNt2). MDN cluster 14 = SMA, MDN cluster 10 = right*

*DLPFC, MDN cluster 13 = left MFG, SCN cluster 1 = left IFG, SCN cluster 4 = right IFG, SCN cluster 5 = SMA. The last figure in each row shows averaged effects across the respective mask. Bars indicate mean, whiskers indicate standard error of the mean (SEM). # = p < 0.05 uncorrected, * = p < 0.05 Bonferroni corrected."*

Reviewer Comment 1d:
Realistically, these broad anatomical areas are functionally heterogeneous and likely include both SC and MD subareas; distinguishing between them would require analyzing responses at the participant level (with relevant localizer tasks or resting-data-based network parcellations). Thus, the group-level analyses employed in this study have somewhat limited power for dissociating effects in MD, SC, and language networks.

<u>Response:</u> *Please refer to our previous replies. We have added subject-specific network-restricted fROI analyses to dissociate MDN and SCN contributions in the SMA/pre-SMA, left MFG and right IFG, and at the respective network level. We have revised the manuscript to clarify our focus on general semantic processing and semantic control and refined our terminology throughout.*

Reviewer Comment 2:
Unclear multiple comparisons in brain-behavior analyses. Showing an association between behavioral responses and TMS-induced brain changes is cool, but the current results seem post hocky, at least in the way they are currently described. Why are local effects only observed for matching trials and remote effects only for mismatching trials? Why do we see increased activation post TMS at the local point impairing performance - from a theoretical standpoint, wouldn't we predict the opposite? How exactly was the whole-brain analyses done for Figure 2C and is the same data used to define that region used to estimate the correlation (which would constitute double dipping and potentially inflate the effect)? Overall, how many comparisons were done in total, were they exploratory or hypothesis-driven, and how was correction for multiple comparisons done?

<u>Response:</u> *We agree that our previous description of multiple comparison corrections was unclear. We also recognize that we did not highlight our piloting-based design and analyses strategies sufficiently in the original version of our manuscript. We have revised the manuscript to clarify multiple comparison correction procedures, number of comparisons and distinguish exploratory from hypothesis-driven analyses. While whole-brain regression analyses and brain-behavior correlation at the stimulation site followed planned decompositions based on behavioral piloting data, further correlations on fMRI-results' ROIs were exploratory. We added reports on non-significant results and FDR-corrected p-values were applicable.*

We have made the following changes to the manuscript:

Methods section (page 33): "This was a planned decomposition analysis, based on differences in behavioral trends from our TMS pilot. Since we restricted our analysis to a small family (n=2) of planned, hypotheses-driven comparisons, we did not perform multiple comparison correction for whole-brain regression models and correlation analyses at the stimulation site, consistent with recommendations by Rothman (1990) and recent complementary suggestions (Boulesteix and Hoffmann 2024; García-Pérez 2023; Hooper 2025)."

Methods section (page 33/34): "Finally, for exploratory analyses, we extracted low intensity-normalized beta-estimates from interaction-derived (left SMG, right PrG, right SPL, right SFG) cluster-peaks of statistical maps (6 mm spherical ROIs) and correlated these with reaction times for correctly solved items (response efficiency). Exploratory analyses were FDR-corrected for multiple comparisons (semantic matches, semantic mismatches)."

Results section (page 12): "Correlations for semantic mismatches did not reveal any significant effects (p > 0.05)."

Results section (page 13): "No voxels survived cluster-level FWE-correction for the semantic matches regression analysis."

Results section (page 16): "Stimulation-induced increases in rPrG were associated with a trend for less stimulation-induced delays in semantic reaction times during semantic match trials (r = -0.414, p = 0.080) but there was no significant correlation for semantic mismatch trials. There was no significant correlation between activity changes in the left SMG and reaction times for any of the conditions." We have updated Figure 4A (page 15) accordingly.

Results section (page 16/17): "Indeed, a negative correlation with semantic reaction times revealed that stimulation-induced increases in activity in the rSFG were associated with less stimulation-induced disruptions in reaction times for semantic mismatches (r = -0.450, p = 0.048, FDR-corrected, 95% bootstrap C.I. [-0.724 -0.117]). No significant effect was found for semantic matches in the rSFG. Finally, for rSPL, there was a trend towards a negative correlation between semantic reaction times and activity changes for semantic matches (r = -0.433, p = 0.062, FDR-corrected) but no significant correlation for semantic mismatches."

Reviewer #2 and #4 suggested additional robustness checks of our significant ROI correlation results (right SFG, left pMTG). We performed both bootstrapping and leave-one-out analyses. Both analyses converged to a robust correlation effect. We have added a description of both procedures to the Methods section (page 34). Please refer to Reviewer #2, Comment 5 for further details.

Additionally, we expanded the Methods section to detail "All regression analyses were performed in SPM12 using multiple regression analysis at p < 0.05 cluster level FWE-corrected, with an initial cluster-defining threshold of p < 0.001 uncorrected." (page 33). We clarified in the figure that the respective ROI correlation analysis is presented for visualization only, as previously indicated in the figure caption. The updated figure looks as follows (Figure 2C, page 13):

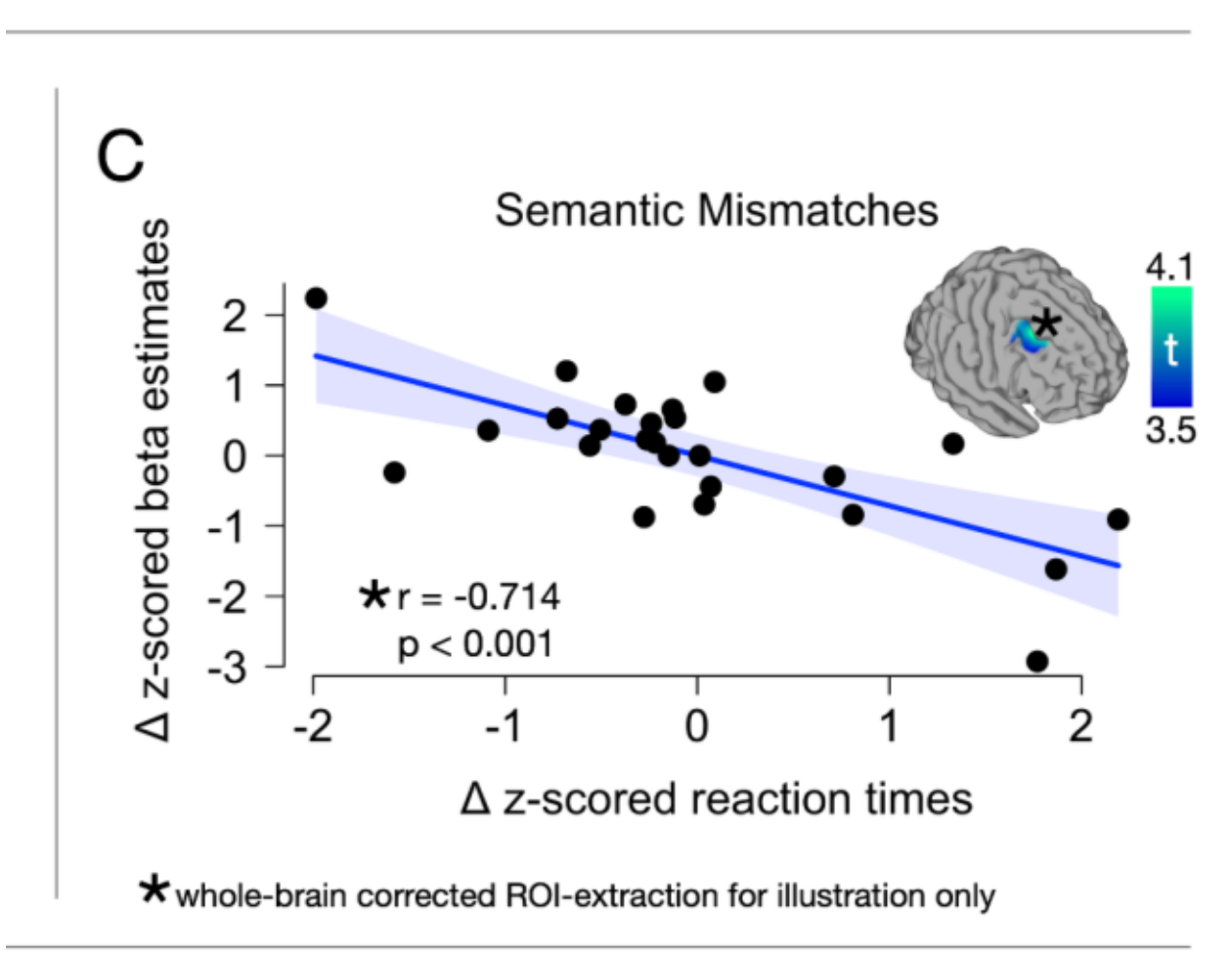

As outlined in the Introduction section, local activity increases during TMS likely reflect increased metabolic demand following externally induced neural perturbation, rather than a specific excitatory or inhibitory process. Given that BOLD signals are insensitive to the direction of underlying neural activity, behavioral measures provide a critical proxy for interpreting these effects; this point has now been clarified in the Discussion section as follows (page 24):

"Importantly, BOLD-fMRI signals are insensitive to whether the underlying neural activity is excitatory or inhibitory. Consequently, behavioral outcomes serve as a proxy for interpreting the direction of these effects."

Reviewer Comment 3a:
Describing and interpreting interaction effects. Why are the brain maps in Figure 3 A and B so different? Wouldn't we expect a consistent effect of increased stimulation, no matter whether the semantic task is contrasted with a perceptual baseline or rest? Is this result surprising or theoretically justified?

Response: *We thank the reviewers for raising these points. Indeed, we would not expect the same interaction effects for perceptual and rest control conditions. With the perceptual control condition, we controlled for visual processing of symbols, visual-spatial attention, decision making and peripheral stimulation effects. In contrast, the rest condition was included to control for the brain state, that is, peripheral stimulation effects during self-referential thinking vs semantic processing. We therefore expected differences in the interaction effects. We have also added an overview of the different control conditions and our motivation to include them as suggested by Reviewer #4. Please see our response to Reviewer #2, Comment 1a and Reviewer #4, Comment 1a for further details.*

Reviewer Comment 3b:
Methodologically, I assume the interaction effects are estimated using the same data as the data that was used to define the clusters, which is an instance of double dipping and is likely to inflate the effect.

Response: *We apologize for the confusion. We would like to stress that cluster activation plots were solely generated to visualize the direction of interactions. This is common practice in*

fMRI research given the multidimensional nature of whole-brain activation changes. Without these plots, the reader would not be able to identify the direction of the interaction effects. To avoid further confusion, we added an explanation to the figure caption stating that these plots (similar to the scatter plot in the whole-brain correlation analysis in Figure 2C) were added for visualization purposes. We would also like to draw the reviewers' attention to the fact that no significance indicators were used in these parts of the figure.

The interaction effects were indeed investigated at the whole-brain level by modelling an interaction term for our respective four task conditions. These maps were cluster-level FWE-corrected (as stated in the manuscript). We extracted activation changes of the respective peak-voxel per condition for visualization purposes. The directionality would be similar in each voxel of this cluster, as revealed by significant whole-brain results. The activations at the selected voxels are an example to provide more information to the reader.

Reviewer Comment 3c:

Finally, there is only one scatterpot per panel, for 1/2 brain areas and 1/2 trial types (match/mismatch). Was multiple comparisons done on these and the rest of the comparisons, that I assume went unreported?

<u>Response:</u> *We thank the reviewer for raising this point. In the main text, we report significant vs non-significant correlations. We regret that multiple comparison correction was not reported consistently in the previous version of our manuscript. To increase transparency, we now consistently report multiple comparison corrections for all exploratory analyses. See also replies to your Comment 2. We would also like to stress that analyses from Figure 2C were performed and corrected at the whole-brain level and not for any specific area. This implies that results for match trials would include the depiction of an empty brain.*

Reviewer Comment 4:

Is there a significant performance difference between semantic and perceptual conditions during the baseline condition? SC regions are usually defined by contrasting hard vs. easy semantic conditions (e.g., Noonan et al. 2013). This is supposed to isolate the semantic-specific controlling component. The current contrast, semantic_gt_perceptual, might yield regions that for semantic cognition and difficulty in general. This may explain why we observe an effect in the left anterior temporal cortex and angular gyrus, beyond the core SC network.

<u>Response:</u> *We agree with the reviewer that the perceptual task might have been easier. However, as depicted in our ROI analysis, contrast estimates in rMFG and rPMd are higher for both perceptual conditions compared to the semantic low-intensity condition but comparable to the semantic high-intensity condition, thus clearly indicating lateralized selectivity of domain-general areas for the perceptual task. The whole-brain correlation analyses were calculated between difference scores separately for semantic and perceptual conditions and should therefore not be biased by any difficulty differences. Whole-brain contrasts actually reflect stronger engagement of semantic and MDN regions for the semantic task, which is in line with our hypothesis for semantic compared to perceptual processing. Moreover, the baseline data show that, in the absence of TMS, the comparison of the semantic vs perceptual task resulted in circumscribed activation changes in the left IFG and pMTG without any above-threshold activation changes in MDN regions. As stated above, our difficulty manipulation was the TMS-induced modulation (high vs low stimulation intensity) during task processing while keeping task-difficulty (stimulus presentation) constant. This may explain*

why we observe an effect in the left anterior temporal cortex and angular gyrus, beyond the core semantic control network.

Reviewer Comment 5:
It would be helpful to talk about the rationale for using 40% rMF as the low-intensity control, especially considering that this condition increases activity in the perceptual but not the semantic condition. How does this yield the same or different effect as the sham control?

Response: *Thank you very much for this important question. Reviewer #1 (Comment 1) and #2 (Comment 1a) asked a similar question, please see our response to their comments above. We note that there is no commercial sham-coil available for intra-scanner TMS and our approach follows common practice in interleaved TMS-fMRI studies of cognition (Schuler and Hartwigsen 2025, BrainStim; Woolgar et al. 2026, NatProt).*

Reviewer Comment 6:
In the Discussion, the potential anterior-to-posterior gradient is discussed, but it is unclear how this relates to any results reported in the Results section.

Response: *We agree with the reviewer that this paragraph might have gone beyond our primary results. We have therefore removed it from the Discussion section.*

Reviewer Comment 7:
Later in the Discussion (line 362-364), the authors mentioned that mismatched trials are more complex and demanding. Is this reflected in behavioral performance? Can this be supported by SI Figure 6, where more left anterior temporal activation in high mismatched vs. matched? Related, in the Supplementary Brain-Behavior Correlations section, here, the behavior is the matched and mismatched combined? The rationale for separating or combining different types of trials can be clearer.

Response: *Thank you for these questions. We have rephrased our assumptions on mismatches to clarify that we were expecting qualitative differences between semantic match and mismatch conditions (as also suggested by supplementary brain maps decomposing match and mismatch trials).*

We have updated the Discussion section as follows (page 23/24):

"This might be explained by different processing strategies for semantic mismatch conditions that require the inhibition of linking concepts rather than actively deciding on a connection between two concepts. These trials require more divergent lexical-semantic search and the ability to inhibit loose associations between presented concepts and thus trigger stronger engagement of frontal resources (Tik et al. 2018; Gao et al. 2022)."

We now also highlight intended decomposition analyses in the Methods section (see also Reviewer #4 Comment 6b):

Page 27: "The number of matches and mismatches was balanced per conditions to enable decomposition analyses of qualitatively different trial types."

Page 33: "This was a planned decomposition analysis, based on differences in behavioral trends from our TMS pilot."

Finally, for full transparency, we added correlations across both semantic conditions to the Supplement (SI Figure 5, page 49).

Reviewer Comment 8:
Line 339-341: "While the effect in the SPL was explained by increased activity solely for the effective high intensity stimulation during the semantic task, the effect in the SFG was driven by decreased deactivation during the same condition."; The effect in the SFG is also driven by stronger deactivation during Rest in high stimulation.

<u>Response:</u> *Thank you for pointing us to this inconsistency. We have rephrased the respective sentence as follows (page 22): "While the effect in the SPL reflected increased activity solely for high-intensity stimulation during the semantic task, the effect in the SFG was driven by decreased deactivation during the same condition, and decreased deactivation during rest for low-intensity stimulation."*

Reviewer Comment 9:
The paragraph from line 345 to line 356 mainly reiterates results. It would be helpful to discuss how to interpret these differential response patterns in MD ROIs.

<u>Response:</u> *Thank you for this suggestion. We have revised the respective passage of the Discussion section to interpret the differential response patterns in MD ROIs as follows (page 23):*

"ROI analyses within the MDN revealed that high- intensity TMS during semantic decisions increased activity in bilateral MFG and SMA, consistent with enhanced domain-general control under elevated semantic demands. The left MFG exhibited increased activity for high vs low stimulation intensity during semantic compared to perceptual decisions, suggesting a left-lateralized bias for semantic control demands.
In contrast, the right MFG and PMd were both upregulated across high stimulation intensity semantic and perceptual conditions, consistent with a role in domain-general difficulty and performance monitoring (Quillen et al. 2021). The SMA showed the strongest response for high stimulation intensity during semantic trials, indicating increased demands on response selection and temporal coordination under semantic perturbation.
Together, these findings suggest that high-intensity TMS transiently elevates semantic control demands, supported by flexible recruitment of MDN regions with partially dissociable domain specificity, in line with models of resilient semantic cognition involving interactions between domain-general and semantic-specific control systems (e.g., Branzi and Lambon Ralph 2023; Hodgson et al. 2024)."

Reviewer Comment 10:
Line 375-378: According to this logic, do we expect to see a correlation between decreased activity in motor areas and increased activity in SC regions? Is there any significant correlation?

<u>Response:</u> We apologize for the confusion. The respective statement in the discussion refers to the results on page 16: "Stimulation-induced increases in rPrG were associated with a trend for less stimulation-induced delays in semantic reaction times during semantic match trials (r = -0.414, p = 0.080) but there was no significant correlation for semantic mismatch trials." We have revised this sentence to clarify our intention (page 24): "Moreover, a trend towards stimulation-induced activity increases in rPrG being associated with less response disruption during semantic match trials suggests less automated processing and increased reliance on semantic control (Davey et al. 2016)."

Minor points

Reviewer Comment 11:
line 255: "less engagement for the semantic low stimulation"; which region? rPMd?

<u>Response:</u> To clarify this issue, we have updated the respective sentence as follows (page 19): "…less engagement for the semantic low stimulation intensity condition relative to all other conditions in the rPMD,…"

Reviewer Comment 12:
Figure 5: It would be helpful to mark the names of regions that are associated with delayed or less delayed responses

<u>Response:</u> Thank you for the suggestion. We have updated the figure (new Figure 6, page 20) accordingly.

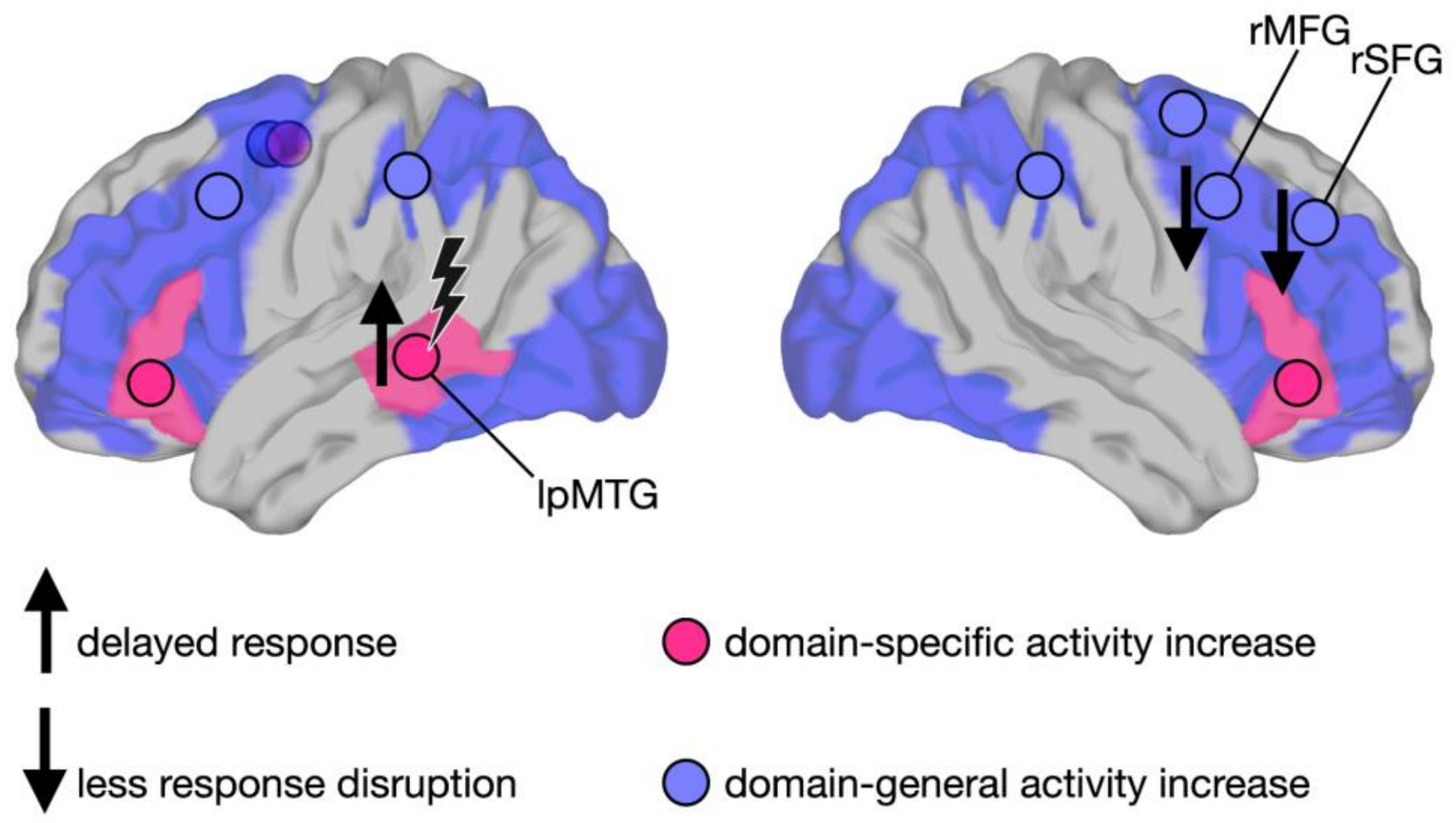

Reviewer Comment 13:
Line 364-367: How does the state dependency of the TMS effects for the semantic task relative to rest support the claim in the previous sentence (i.e., mismatched condition creates a more vulnerable brain state)?

Response: *We acknowledge that these sentences were confusing. We have removed them from the Discussion section.*

Reviewer #4:
The authors use interleaved TMS-fMRI to test how the brain responds immediately to perturbation of a key semantic-control node (left pMTG) during an adaptive semantic matching task. The approach is technically ambitious (delivering bursts within a ~520 ms acquisition gap) and the design is unusually strong for causal neuroimaging: a within-subject manipulation with multiple, complementary controls (perceptual control task, intensity manipulation, a no-TMS baseline, and a stimulation-at-rest run). The central pattern - local semantic-network effects linked to slowing, alongside remote domain-general recruitment linked to preserved performance - is compelling and fits well with prominent network-flexibility accounts. More broadly, this work is a methodologically impressive application of interleaved TMS-fMRI to semantic control and is well positioned to serve as a useful reference for acute network adaptation during language/semantic processing.

The manuscript is already strong. In my view, the main claims would be further strengthened primarily by: (i) making the handling of peripheral/sensory confounds even more explicit in the main narrative using controls the authors already include (especially the rest run and task control), and (ii) adding (or briefly motivating why not feasible) a connectivity-based test of the proposed compensatory mechanism linking right frontal recruitment to semantic-network support. With these clarifications/additions, I expect this paper will make a strong contribution to both the concurrent TMS-fMRI and semantic cognition literatures.

Response: *We thank the reviewer for their enthusiastic evaluation of our manuscript. We have made our control conditions more explicit and added additional functional connectivity results. Your suggestions helped us to substantially improve our manuscript.*

Major comments

Reviewer Comment 1a:
Peripheral/sensory confounds: make the logic maximally explicit using the existing controls. A key interpretive challenge in concurrent/interleaved TMS-fMRI is that "high vs low"; intensity can differ in auditory/somatosensory sensations and arousal/startle, which may influence behavior and BOLD responses in nonspecific ways. The authors already acknowledge these issues and, importantly, the current design is well equipped to address them (perceptual control task and stimulation-at-rest run). I recommend making this inferential logic especially explicit in the main Results/Discussion so readers can immediately see why the key conclusions are not driven by generic intensity effects.

Suggestions:
Center the inference on state-/task-specific effects (e.g., Task by Intensity interactions and/or semantic vs rest comparisons), rather than emphasizing simple high_gt_low maps. Even if the underlying model already includes this, showing it clearly (main text and/or a compact figure panel) would help readers understand how nonspecific intensity consequences are controlled.

Response: We thank the reviewer for this great suggestion. We have added a paragraph on discussing control conditions to the Methods section (page 28), integrated an overview of control conditions in Figure 1 (now 1B, page 7) and refer to control conditions in the Results section (page 6). Please refer to Reviewer #2, Comment 1a for further details.

Reviewer Comment 1b:
Elevate the rest-run check in the main narrative (rather than leaving it feeling secondary). A concise statement like "effects consistent with compensation are state-dependent and not present (or are qualitatively different) at rest"; would go a long way.

Response: Thank you for this helpful suggestion. Reviewer #2 made a similar suggestion, please refer to our response to Comment 3b by Reviewer #2.

Reviewer Comment 1c:
Discomfort/pain ratings: If subjective discomfort/loudness ratings were collected, reporting them (even briefly) would further strengthen this point. If not collected, a one-sentence limitation acknowledging this would be sufficient.

Response: We administered the questionnaire by Giustiniani et al. (2022). Results are now summarized in SI Table 1.

We have added the following description to the Methods section (page 28):

"Participant-specific TMS side effects were assessed with the TMSSens_Questionnaire (Giustiniani et al. 2022; SI Table 1)."

SI Table 1 can be found on page 45:

"TMS Side Effects
SI Table 1. Quality, frequency and intensity of TMS side effects.

Effect	***Number of Participants***	***Range***
Neck Stiffness	*3*	*mild-moderate*
Headache	*8*	*mild-considerable*
Muscle Contractions	*11*	*mild-strong*
Scalp Pain	*11*	*moderate-strong*
Difficulty Concentrating	*11*	*mild-considerable*
Fatigue	*2*	*mild*
Sleepiness	*7*	*mild-considerable*
Coil Pressure	*11*	*mild-strong*
Anxiety/Nervousness	*6*	*mild-considerable*
Intrinsic Noise Perception	*1*	*mild*

Tingling Sensations	*7*	*mild-moderate*
Skin Sensation	*2*	*moderate-considerable*
Negative Mood	*2*	*moderate-strong*
Burning Sensation	*1*	*considerable*

"

Reviewer Comment 2:
Make the "compensation"; interpretation more directly testable with connectivity. At present, compensation is inferred primarily from (i) remote upregulation in domain-general regions and (ii) brain-behavior associations in right frontal areas. This is promising and theoretically well motivated. However, a connectivity analysis would make the mechanistic interpretation substantially more persuasive, because it would test whether the "helpful"; remote recruitment is functionally integrated with the semantic network during perturbation.

Suggestion:
Add a task-dependent connectivity analysis (e.g., PPI/gPPI), using seeds in right MFG/rSFG and/or canonical semantic-control nodes (e.g., left IFG/pMTG). The key prediction would be stronger coupling between right frontal regions and semantic-control/semantic areas specifically during high-intensity semantic blocks (and/or higher-demand conditions).
If full PPI is not feasible, a focused ROI-to-ROI connectivity analysis (semantic-control; MDN nodes) would still directly test redistribution accounts.
If connectivity is not feasible (e.g., design constraints, power), a brief statement explaining why would help align expectations with what the current data can support.

<u>Response:</u> *Thank you for this great suggestion. We calculated additional PPI analyses for semantic high-intensity stimulation vs semantic low-intensity stimulation using the stimulation site (left pMTG) and the "compensatory" rMFG region as seed regions. While connectivity between the stimulated region and right frontal areas decreased, the compensatory right MFG seed showed increased coupling with the pre-SMA—an interface between semantic control and multiple-demand networks. As this finding strengthens our main conclusions, we have included the PPI analysis as a separate result in the main Results section.*

We have added the following to the Methods section (page 32):

"Generalized Psychophysiological Interaction (gPPI)
To elucidate TMS intensity specific connectivity modulations, we used the CONN toolbox (22.v2407). Four task conditions were modeled as miniblocks without HRF convolution (semantic high, semantic low, perceptual high, perceptual low). Preprocessing was skipped, since all necessary preprocessing steps had been performed for initial GLM-estimations. The lpMTG was defined as a thresholded cluster from a TMS-independent semantic network contrast derived from our baseline no TMS run. As a second ROI, we used the significant cluster in the rMFG derived from our brain-behavior regression analysis (see below). These ROIs were imported into CONN as single-timeseries seeds, and whole-brain seed-to-voxel connectivity analyses were computed at the subject level. At the second level, we tested stimulation condition contrasts on gPPI effects (semantic high > semantic low) using voxelwise t- tests within CONN. Resulting seed-to-voxel t-maps were thresholded at the voxel level ($p < 0.001$, uncorrected) with cluster-level FWE correction at $p < 0.05$. Significant clusters were

interpreted as regions showing condition-dependent increases or decreases in lpMTG or rMFG connectivity."

The following description was added to the Results section (page 14/15):

"Task-based connectivity explains local disruption effects and remote compensation
For an in-depth investigation of the compensatory dynamics between semantic and domain-general regions, we performed task-based connectivity analyses (gPPI) between the targeted lpMTG as well as compensatory rMFG and all other areas in the brain, respectively. Comparing connectivity changes between the semantic high-intensity and low-intensity stimulation conditions, we observed decreases in the interaction between the lpMTG (seed region) and rMFG/SFG (x, y, z = 30, 22, 58; t > 3.75, p = 0.034; Figure 3, left panel) and between the rMFG (seed region) and the lpMTG (x, y, z = -52, -58, 2; t > 3.75, p < 0.001; Figure 3, right panel). In contrast, coupling between the rMFG and the pre-SMA—an intermediate zone between semantic control and multiple demand regions—increased (x, y, z = 0, 20, 40; t > 3.75, p = 0.028; Figure 3, right panel).

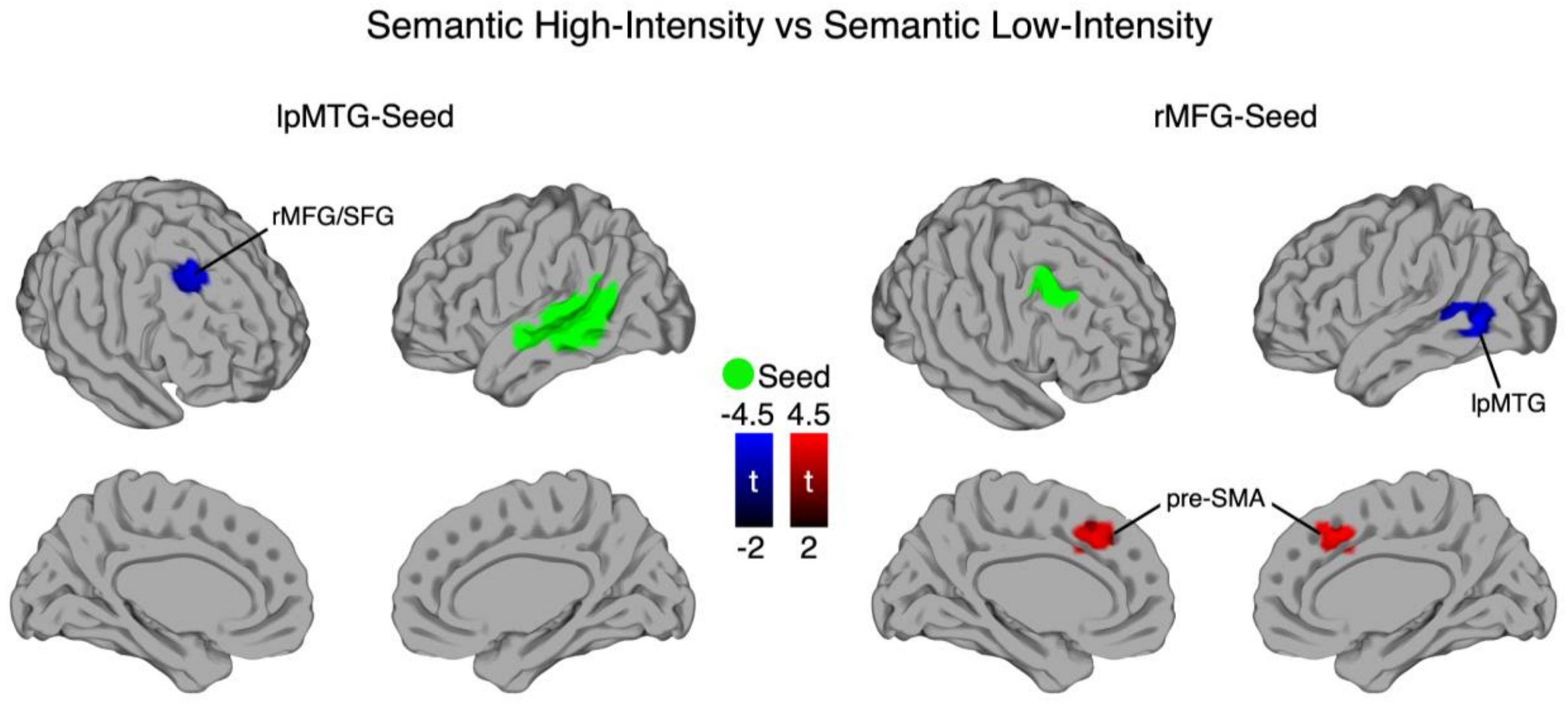

Figure 3. TMS-induced connectivity changes during semantic processing. *Left Panel. Seed-based connectivity between the lpMTG and the rest of the brain showed significant connectivity decreases between the lpMTG and rMFG/SFG. Right Panel. Seed-based connectivity between the rMFG and the rest of the brain revealed significant connectivity decreases between the rMFG and the lpMTG and increases between the rMFG and the pre-SMA. Blue t-values: negative connectivity, red t-values: positive connectivity. Thresholded at p < 0.001 uncorrected with cluster level FWE correction at p < 0.05."*

We have added the following to the Discussion section (page 21):

"Complementary whole-brain connectivity analyses showed disrupted task-related connectivity between the lpMTG and rMFG/SFG for semantic high- vs low-intensity conditions. This decrease in connectivity under effective stimulation may indicate less efficient coordination of control resources and likely contributed to delayed performance on demanding

(high-intensity) semantic trials. By contrast, upregulation of an adjacent rMFG subregion and its increased functional coupling with the pre-SMA—a hub at the interface of semantic and domain- general control—likely reflects compensatory network reconfiguration following transient disruption of semantic processing (Chiou et al. 2023).”

Reviewer Comment 3:
Clarify ROI definition/independence (preempt circularity concerns). The MDN ROI findings are interesting and support the domain-general recruitment narrative, but you clarify how ROIs were defined and whether they were independent of the contrasts used for hypothesis testing.

Suggestions:
Add a clear statement specifying whether ROI centers are drawn from an independent atlas/prior coordinates versus derived from peaks in the present contrasts.
If any ROIs were derived from the present data, consider a leave-one-subject-out approach or a fully atlas-based alternative for the main confirmatory ROI tests (with current ROIs retained as exploratory).

<u>Response:</u> *Thank you for this suggestion. The MDN ROIs were indeed data independent (as specified in the Methods section (page 32): “For in-depth exploration of the impact of TMS on the recruitment of domain-general areas of the multiple demand network, we extracted contrast estimates for the four regressors semantic high, semantic low, perceptual high and perceptual low from 7 key regions of the multiple demand network, including bilateral anterior insula (antIns), supplementary motor area (SMA), bilateral dorsal premotor cortex (PMd) and bilateral middle frontal gyrus (MFG) based on a canonical MDN reference map (Fedorenko et al. 2013; https://imaging.mrc-cbu.cam.ac.uk/imaging/MDsystem).” All other ROI results were derived from study specific locations. Following the reviewer’s suggestion, we added leave-one-out analyses in addition to bootstrapping results as suggested by Reviewer #2 (Comment 5; SI Table 2, page 50). Both measures converge and support our correlation results.*

Reviewer Comment 4:
Brain-behavior correlations: clearly distinguish confirmatory vs exploratory and state the correction approach. Multiple brain-behavior correlations are reported (local and remote; sometimes split by match/mismatch). Given their importance for the mechanistic story, we would benefit from knowing which relationships were hypothesized a priori and how the paper handles the multiplicity of tests.

Suggestions:
Label correlations (or families of correlations) as confirmatory vs exploratory
Briefly state the strategy for multiple comparisons (e.g., FDR across a defined family, or a small hypothesis-driven set highlighted in the main text with the remainder described as exploratory).
Where feasible, consider supplementing difference-score correlations with a single regression-style framework (e.g., predicting RT or inverse-efficiency with Task/Intensity/Match and relevant interactions) to reduce noise and simplify interpretation.

<u>Response:</u> *Thank you very much for this suggestion. We have clarified the distinction between hypotheses-driven and exploratory analyses throughout the manuscript and revised the explanation of multiple comparison procedures. Please refer to Comment 2a by Reviewer #2*

and Comment 2 by Reviewer #3 for further details. For our correlation analyses, we retained difference scores, as our hypotheses concerned within-subject effects of semantic processing across high- and low-intensity TMS conditions. Difference scores provide a direct numerical representation of this contrast, facilitating straightforward interpretation.

Reviewer Comment 5a:
Alternative behavioral modeling. The behavioral results reassuringly show minimal (or no) high vs low differences in RT/accuracy within task, which supports the interpretation that neural effects are not trivially explained by gross behavioral differences. Because the mechanistic argument leans on brain-behavior relationships, a trial-level model could provide additional leverage and a clean way to integrate Task/Intensity/Match in one place.

Suggestions:
Consider adding a trial-level mixed-effects model (LME/GLMM) with Task, Intensity, Match, and interactions, with subject-level random effects. This uses all trials and avoids some limitations of condition medians.

<u>Response:</u> *We have added LME models for behavioral outcomes. We have added the following to the Methods section (page 30):*

"Behavioral Data
Reaction times (RT) were analyzed using linear mixed-effects regression with the lme4 and lmerTest packages in R (RStudio 2022.07.1). The dependent variable was z-standardized log-transformed RT for correctly solved items. Fixed effects included task, intensity, and match and their interactions. The random-effects structure comprised random intercepts for participants and items. Trial-level accuracy (correct = 1, incorrect = 0) was analyzed using generalized linear mixed effects models with a binomial error distribution and logit link. Fixed and random effects mirrored the RT model, with task, intensity, and match (and their interactions) as fixed effects, and random intercepts for participants and items."

We have added the following to the SI Results section (page 46-48):

"Behavioral Results
LME results for reaction times (RT) showed a main effect for task ($F(1, 997.23 = 166.87$, $p < 0.001$) and match ($F(1,1082.39) = 28.57$, $p < 0.001$) and a significant interaction between both factors ($F(1,1082.89) = 60.49$, $p < 0.001$). Participants were faster for perceptual mismatch trials compared to the other conditions. Simple effects were estimated with a linear mixed-effects model (Kenward-Roger degrees of freedom). Overall, the task x match interaction reflects a larger semantic - perceptual difference for mismatch trials (semantic - perceptual = 0.91, SE = 0.06, df = 941, $t = 15.07$, $p < 0.001$) compared to match trials (semantic - perceptual = 0.23, SE = 0.06, df = 914, $t = 3.55$, $p < 0.001$). The effect of match was driven by the perceptual task: for the semantic task, there was no significant difference between mismatch and match conditions (mismatch - match = 0.11, SE = 0.06, df = 1112, $t = 1.70$, $p = 0.089$), whereas for the perceptual task, reaction times were shorter for mismatch than match trials (mismatch - match = -0.58, SE = 0.06, df = 845, $t = -9.28$, $p < 0.001$). LME results for accuracy revealed a main effect for match ($p < 0.001$) with matching trials showing lower accuracy than mismatching trials (beta = 1.61, SE = 0.31, $z = -5.25$, $p < 0.001$) as well as a task-by-match interaction (beta = 2.10, SE = 0.48, $z = 4.36$, $p < 0.001$). Participants had less accurate

performance during semantic matches compared to the other conditions. A simple-effects analysis confirmed that task had no significant effect on accuracy for mismatch trials (semantic vs perceptual: OR (odds ratio) = 1.24, z = 0.86, p = 0.39), but did so for match trials, where the odds (odds ratio) for a correct response in the semantic task were lower than for the perceptual task (semantic vs perceptual: OR = 0.19, z = -7.36, p < 0.001).

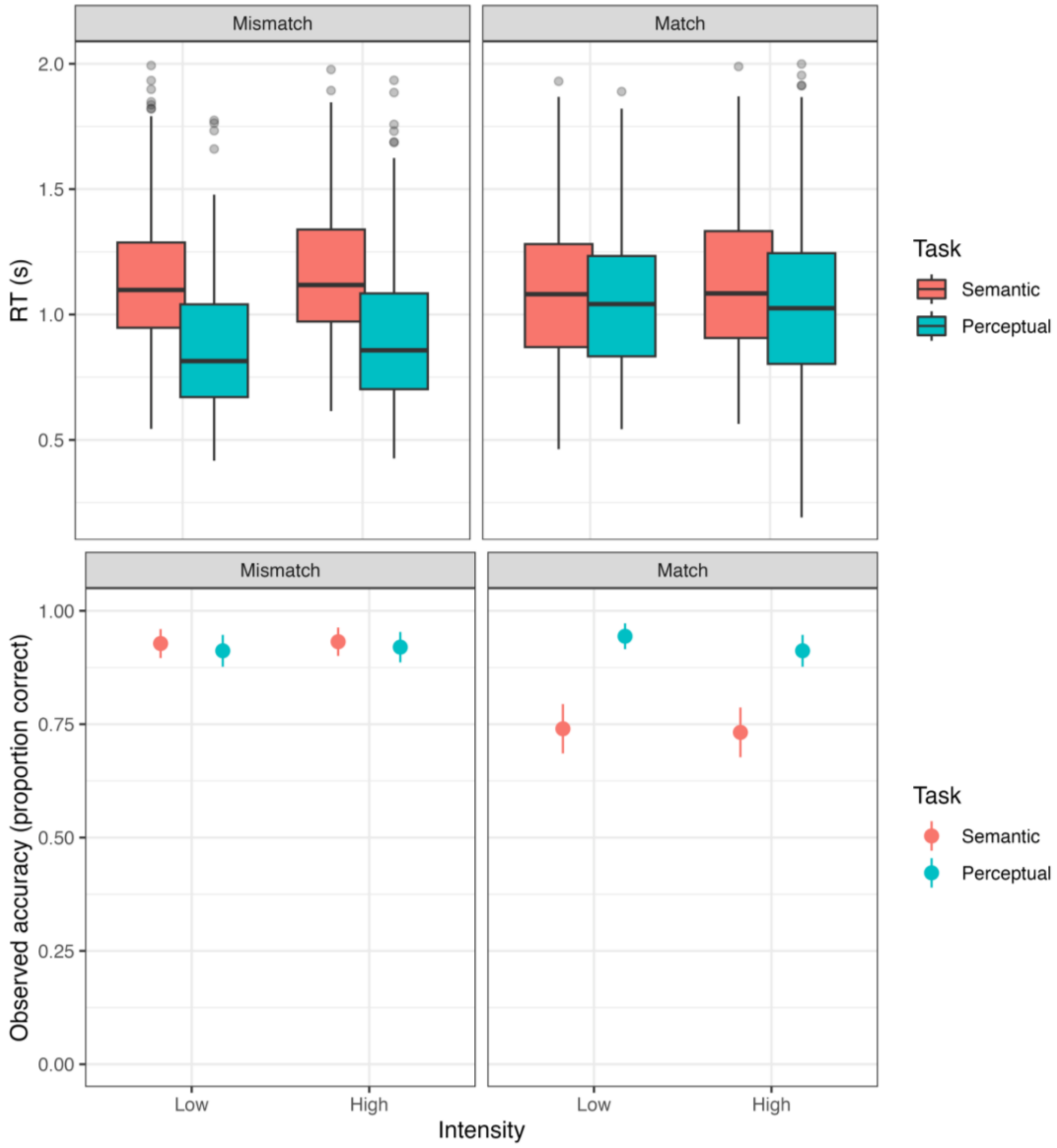

SI Figure 3. Reaction times (correct responses) and accuracies during TMS. *No significant differences were found between high and low stimulation intensity conditions. Participants showed faster reaction times for perceptual mismatches relative to all other conditions. Accuracy was lower for semantic matches compared to all other conditions. Boxes indicate 25th percentiles, whiskers indicate 50th percentiles and dots indicate values exceeding the 50th percentile. For accuracy, dots indicate mean accuracy proportion and whiskers 95% confidence intervals."*

Reviewer Comment 5b.

If the authors want to emphasize "no behavioral confound,"; an equivalence test (or Bayes factors) for high vs low within each task can communicate evidence for no meaningful difference (rather than a null result).

<u>Response:</u> We have now added equivalence tests to support the behavioral confound statement:

SI Methods (page 43): "Equivalence Tests
Reaction times were analyzed separately for each task x match condition. For each participant and condition, we computed mean reaction times for low- and high-intensity TMS and derived within-subject difference scores (high - low; positive values indicate slower responses under high intensity). Across participants, we then calculated the mean difference, standard deviation, and confidence intervals.
We evaluated within-subject reaction time differences between high- and low-intensity TMS using the two one-sided tests (TOST) procedure for equivalence testing (Schuirmann 1987). Equivalence bounds were set to 0.2 of the raw RT difference scores. Equivalence was assumed when both one-sided tests were significant at α = .05, which is equivalent to the 90% confidence interval for the mean difference lying entirely within the equivalence bounds."

Results section (page 9): "Note that the impact of TMS on task-related activity was not confounded by stimulation-induced changes at the behavioral level (SI Methods and Results (Equivalence Tests), and SI Figure 3)."

SI Results (page 48): "Equivalence Tests
Across all task x match conditions, within-subject reaction time differences between high- and low-intensity TMS were small and within the predefined equivalence bounds of -0.2 to +0.2. For semantic mismatches, the mean difference was 0.042 (SD = 0.127; 90% CI [-0.002, 0.085]), and the equivalence test was significant. For semantic matches, the mean difference was 0.037 (SD = 0.189; 90% CI [-0.028, 0.101]) with significant equivalence. For perceptual mismatches, the mean difference was 0.036 (SD = 0.128; 90% CI [-0.008, 0.080]) and again supported equivalence. For perceptual matches, the mean difference was essentially zero (-0.001; SD = 0.196; 90% CI [-0.069, 0.066]), and the equivalence criterion was also met."

Reviewer Comment 6a.
Interpretation: sharpen domain-general control vs arousal/difficulty and keep match/mismatch aligned with evidence strength. The authors already makes a nuanced argument about state dependency and demand (e.g., mismatch trials). I recommend a brief expansion that explicitly contrasts two interpretations and ties them to the existing controls.

Suggestions:
Arousal/startle/peripheral effects: should be broad (semantic + perceptual) and potentially present at rest.
Domain-general control compensation: should be state- and demand-dependent, and should relate to preserved performance.

<u>Response:</u> We thank the reviewer for raising this point. We have adjusted the Discussion section on page 22/23 as follows.

"These observations provide novel insights into both the mechanisms of TMS and the dynamic allocation of cognitive resources. Combining different control conditions (task-, state-specificity and general side effects control), we were able to dissociate unspecific side effects

evoked by arousal, startle responses, or peripheral effects from compensatory mechanisms in response to TMS."

Reviewer Comment 6b:
Relatedly, if the key Intensity; Task; Match interaction is not significant, I recommend keeping the main claims centered on supported effects and framing match/mismatch decompositions as exploratory unless clearly powered and pre-specified.

<u>*Response:*</u> *Thank you for raising this point. After piloting, we indeed adjusted the main protocol to allow for planned decomposition of stimulus effects to be able to map potentially subtle but relevant differences in solving qualitatively different items. We have clarified this in the Methods and Results sections as follows:*

Methods section (page 27): "The number of matches and mismatches was balanced per conditions to enable decomposition analyses of qualitatively different trial types."

Methods section (page 33): "This was a planned decomposition analysis, based on differences in behavioral trends from our TMS pilot."

Results section (page 12): "This was a planned decomposition since behavioral piloting data hinted towards qualitatively different processing strategies for both trial types."

Minor comments / clarity edits

Reviewer Comment 7.
Define "semantic efficiency"; at first use (e.g., correct RT / inverse-efficiency) and keep terminology consistent.

<u>*Response:*</u> *Thank you for this suggestion. We now consistently use "reaction times" throughout the manuscript. We clarify that this means correct reaction times upon first mention.*

Reviewer Comment 8:
If follow-up contrasts are presented after a non-significant higher-order interaction, label them clearly as planned decompositions or exploratory (as appropriate).

<u>*Response:*</u> *Thank you for this suggestion. While the main focus of our study was to identify TMS effects during semantic processing, the study was designed to allow for planned decompositions acknowledging behaviorally different cognitive strategies for matches vs mismatches (see Response to Comment 6b).*

Reviewer Comment 9:
Consider adding one brief sentence situating the semantic-control framing within a standard model (e.g., controlled semantic cognition) for general readers.

<u>Response:</u> We have added the following sentence to the Introduction section (page 3): "Notably, semantic control requires both, the controlled retrieval of semantic concepts and the ability to solve conflicting semantic information (Lambon Ralph et al. 2017)."

Reviewer Comment 10.
Ensure motion/tSNR quality checks are easy to find (a compact table or brief summary is sufficient).

<u>Response:</u> We thank the reviewer for this suggestion. We have added a summary on motion/tSNR quality checks to the Supplement and referenced it in the Methods section (page 29):

"For an overview on motion and tSNR quality checks, please refer to SI Table 3."

SI Table 3 can be found on page 53:

SI Table 3. Motion and tSNR quality checks.

Framewise displacement (translational motion parameters)			
	No Task	***Task***	***Task-TMS***
mm	*0.088 (0.026)*	*0.083 (0.026)*	*0.092 (0.028)*
tSNR (ROI)			
Left MFG	*35.063 (8.051)*	*38.119 (6.952)*	*30.768 (7.176)*
Right MFG	*46.291 (11.746)*	*52.619 (12.448)*	*37.732 (10.052)*
Right SFG	*42.586 (9.528)*	*47.837 (10.908)*	*36.156 (10.67)*
Left OpIFG	*47.847 (10.302)*	*53.987 (13.926)*	*41.637 (11.843)*
Right OpIFG	*45.980 (14.038)*	*55.652 (15.686)*	*38.566 (11.912)*
Left TrIFG	*31.345 (8.162)*	*36.148 (11.153)*	*26.780 (9.089)*
Right TrIFG	*34.421 (12.782)*	*41.431 (13.050)*	*30.094 (10.114)*
Left MTG	*37.835 (9.457)*	*44.510 (13.106)*	*30.951 (8.311)*
Left STG	*46.460 (11.765)*	*54.609 (15.797)*	*37.859 (10.176)*
Left SMG	*57.859 (15.917)*	*66.281 (17.562)*	*48.183 (15.129)*
Right SMG	*48.919 (12.736)*	*53.121 (11.062)*	*42.769 (13.302)*
Left SPL	*45.341 (9.573)*	*47.991 (8.591)*	*40.976 (9.912)*
Right SPL	*34.497 (11.025)*	*37.375 (10.741)*	*32.455 (11.329)*

All values presented as mean (SD). tSNR was calculated on unsmoothed data. ROIs were defined based on Neuromorphometrics anatomical atlas.

Average subject motion was low (<1mm) and tSNR was fair to good for all relevant brain areas (>20) and across the three runs.

Reviewer Comment 11:
Make the rationale for the cluster correction approach explicit (e.g., CDT p<.001 with cluster-level FWE), primarily to preempt predictable reviewer concerns.

<u>Response:</u> We have changed the respective sentence as follows (page 31): “Statistical thresholds were set to $p < 0.05$ cluster level FWE-corrected, with an initial cluster-defining threshold of $p < 0.001$ uncorrected.”

Reviewer Comment 12:
Briefly justify the choice of 40% RMT as the control intensity and clarify what it controls (and what it does not).

<u>Response:</u> This was clarified in response to your Comment 1a.

<u>Reviewer #5:</u>
I co-reviewed this manuscript with one of the reviewers who provided the listed reports. This is part of the Nature Communications initiative to facilitate training in peer review and to provide appropriate recognition for Early Career Researchers who co-review manuscripts.

<u>Response:</u> Thank you for your constructive feedback that helped us to improve our manuscript.

<u>Response Letter</u>

We thank the editor and reviewers for their continued thoughtful and constructive feedback, which has further improved the clarity and rigor of the manuscript. Below, we provide point-by-point responses to all remaining comments. Revisions introduced in response to these comments are indicated in *italics* in this letter and highlighted in red in the manuscript.

<u>Reviewer #1</u>

Dear Authors,

Thank you for the comprehensive answers to all te comments of all the reviewers. Indeed your work is important and pioneering!

<u>Response:</u> We would like to thank you again for spending the time and effort to review our manuscript.

Reviewer Comment 1:

I have one more suggestion, that can be ignored. Can you please simplify the title a bit. Although it is detailed and informative it can be difficult for a more general readership, although it is indeed a difficult task

<u>Response:</u> We thank the reviewer for this suggestion. We have simplified the title to accommodate the general readership at Nature Communications: "Disrupting the Semantic Network Shifts Activity to Domain-General Brain Regions"

<u>Reviewer #2</u>

I have read the revised manuscript and the authors' responses to the reviewers' comments with interest. Overall, I think the authors have made a substantial effort to address the concerns raised during review. The revised manuscript is clearer in its theoretical motivation, better structured in its presentation of the control conditions, and more cautious in several places regarding the interpretation of TMS-induced activity changes as compensatory mechanisms.

I still think several points should be treated with caution before publication.

<u>Response:</u> We are pleased the reviewer is generally satisfied with our revisions. We have addressed the remaining suggestions by the reviewer point-by-point below.

Reviewer Comment 1:

First, the term "compensatory" remains somewhat stronger than what the data can directly establish. The negative brain–behavior correlations in right frontal regions are consistent with compensation, but they do not by themselves demonstrate a causal compensatory mechanism. This is especially important because BOLD activity cannot determine whether the underlying neural activity is excitatory or inhibitory, a limitation that the authors themselves acknowledge. I therefore recommend that the authors consistently use more cautious language, such as "putative compensatory recruitment" or "activity patterns consistent with compensation," particularly in the Abstract, Discussion, and Figure 6 legend.

<u>Response:</u> *We thank the reviewer for this suggestion. We have toned down our statements on compensatory recruitment throughout the manuscript. For example, in the Abstract (page 2), we now state:*

"Using transcranial magnetic stimulation interleaved with functional magnetic resonance imaging, we investigated acute network adaptation when the left posterior middle temporal gyrus—a core lexico-semantic region—was perturbed during lexico-semantic decisions in 25 healthy participants."

[...] "These findings indicate that acute semantic disruption triggers immediate, flexible recruitment of domain-general control areas consistent with the maintenance of task performance, highlighting the brain's capacity to dynamically adapt to interference. Our findings contribute to the current understanding of putative compensatory network-level mechanisms supporting lexico-semantic cognition."

*Figure legend 6 (page 20) now reads: "**Figure 6. Perturbation of left pMTG triggers acute semantic network responses and domain-general activity shifts associated with preserved performance.** Summary of the main findings. Visualization of immediate activity changes in domain-specific semantic areas (pink circles) and domain-general regions (purple circles). Local activity increases in the pMTG were associated with delayed lexico-semantic reaction times, while activity increases in remote domain-general areas were correlated with less disruption in lexico-semantic reaction times. Purple mask: outline of the domain-general multiple demand network."*

Reviewer Comment 2:
Second, the sample size remains relatively modest for individual-difference correlations between stimulation-induced BOLD effects and behavioral modulation. The reported correlations are theoretically interesting, but some are close to conventional significance thresholds. The manuscript would benefit from a clearer statement that these brain–behavior associations should be interpreted as converging evidence rather than definitive proof of compensation. This is particularly relevant for the planned match/mismatch decomposition and for the decision not to correct some planned correlation analyses for multiple comparisons.

<u>Response:</u> *We thank the reviewer for this suggestion. We have now added a Limitations section which includes the following statement (page 26):*

"[...] Third, although we included a relatively large sample compared to typical interleaved TMS-fMRI studies of cognition (cf. Schuler and Hartwigsen, 2025), the observed brain-behavior correlations should be interpreted as converging rather than definitive mechanistic evidence for compensatory processes. Future studies should further examine the functional relevance of putative compensatory upregulation."

Reviewer Comment 3:
Third, while the multi-level control framework is a strength, the low-intensity stimulation condition cannot fully separate auditory/somatosensory effects from intensity-dependent neural effects, as the authors acknowledge. This limitation should remain visible in the Discussion rather than being treated as fully resolved by the design.

<u>Response:</u> *We agree with the reviewer and discuss this now as a potential limitation. We have made the following change to the manuscript (page 26):*

"First, despite being an established control condition in interleaved TMS-fMRI research (Schuler and Hartwigsen 2025; Woolgar et al. 2025), the low-intensity stimulation condition cannot fully separate auditory/somatosensory effects from intensity-dependent neural effects."

Reviewer Comment 4:
In summary, I find the revised manuscript substantially improved and scientifically valuable. The study offers a technically sophisticated and novel contribution to the literature on semantic control, TMS-fMRI, and flexible network adaptation. Provided that the authors further moderate the causal/compensatory language and explicitly acknowledge the remaining limitations concerning sample size, correlational interpretation, and translational claims, I would support publication after minor revision.

<u>Response:</u> *We thank the reviewer again for their positive evaluation of our manuscript. We have revised our statements on potential compensatory effects, toned down our translational claims (see first reply to Reviewer #3 below for details) and discuss the limitations of our study (including sample size and correlational interpretation) in the new Limitations section of the discussion.*

<u>Reviewer #3</u>
All reviewers note the technical innovation of the work, which is indeed commendable. However, our concerns here center primarily on the scientific (statistical) validity of the findings, as well as the theoretical framing. The authors state that their results might be relevant for aphasia treatment development, and so it's really important to be confident in the implications that this work does or does not have for aphasia, as well as the overall strength of the work. We are worried that, if published in a high-impact journal like Nature Comms, the results of the work might be perceived as stronger as they are. We are listing the core concerns below, in order of importance.

<u>Response:</u> *We agree with the reviewers' concerns regarding the interpretation of our findings and their potential implications for aphasia therapy. Given the novelty of our approach and findings, the interpretation of the results and their potential translational implications should be presented with appropriate caution. We acknowledge that our study provides only a preliminary contribution toward a more dynamic understanding of recovery following brain lesions. Accordingly, we have revised the manuscript to better reflect the scope of our findings and to avoid overstating their potential implications for aphasia therapy. In particular, we toned down statements in the Introduction and Discussion sections regarding the potential translational relevance:*

Page 4: "Moreover, elucidating dynamic adaptation during lexico-semantic cognition may not only inform neurobiological models of lexico-semantic processing but also provide initial insights relevant to future neurostimulation approaches for patients with lexico-semantic impairments following brain lesions."

Page 25: "Future studies could investigate these mechanisms further to determine whether targeted non-invasive brain stimulation of domain-general areas may represent a potential avenue for supporting recovery in patients with impaired lexico-semantic processing after brain lesions. In particular, frontal nodes of the multiple demand network (MDN) may represent promising stimulation targets following damage to lexico-semantic brain regions, although this possibility requires further investigation."

Below, we address all other suggestions by the reviewers point-by-point.

Reviewer Comment 1:
The link between brain response and behavior. In our original review, we asked which analyses were prespecified and which were exploratory, and whether adjustments for multiple comparisons have been made. The authors responded that they fit separate models for correct and incorrect responses based on pilot data. However, their own method description says that the main model does not include response correctness and that "For exploratory purposes, an additional model was estimated that incorporated matches vs mismatches as a third factor." Thus, this result is purely exploratory, which is not reflected in the framing. It is also not quite consistent with the authors' original hypothesis, which does not posit different effects for matches and matches - that fact too should be reflected. Finally, a multiple comparison correction should be done here and was not - the authors run separate tests for matches and mismatches and even if only one is significant, they treat it as evidence in support of their theory. This is a classic situation where a multiple comparison adjustment would be needed - and if applied, it might actually make one of the results non-significant. So overall, we just don't have faith in the brain-behavior results and the replicability of these findings.

<u>Response:</u> *We thank the reviewers for this comment and appreciate the opportunity to clarify the analysis structure and the role of our pilot data. We further apologize for the lack of clarity in the previous revision.*

First, we did not fit separate models for correct and incorrect responses in the pilot sample or in the main experiment. Rather, as described in the Methods (page 27), our task included a set of predefined semantic matches (e.g., "calendar"–"date") and mismatches (e.g., "pear"–"ring").

Second, in the pilot experiment, we examined the effects of TMS on task performance across different stimulation onsets separately for predefined semantic match and mismatch trials, and observed qualitatively different modulation patterns for the two trial types (matches vs mismatches) in accuracy and reaction times at a delay of 200 msec. We now state the following in the Supplementary Information (page 44):

"Performance at this timing also hinted towards qualitative differences in TMS effects–slower reaction times for mismatches_high vs matches_high (Cohen's d = 0.284; $p_{Wilcoxon} > 0.1$), suggesting a small but not negligible effect and lower accuracies for matches_high vs mismatches_high (Cohen's d = 1.605, $p_{Wilcoxon} = 0.014$) suggesting a strong effect. Descriptive statistics are displayed in SI Table 1. Based on these data, we planned decomposition analyses for matches and mismatches at the behavioral level for our main experiment."

SI Table 1. Descriptive TMS pilot data results at 200 msec delay

Measure	***Mismatches High***	***Mismatches Low***	***Matches High***	***Matches Low***
Reaction Times	*1.346 sec (0.164)*	*1.320 sec (0.174)*	*1.295 sec (0.099)*	*1.285 sec (0.208)*
Accuracy	*100% (0%)*	*100% (12.4%)*	*59% (20.3%)*	*73% (20.2%)*

All values presented as median (interquartile range, IQR). Reaction times are displayed in seconds, accuracy in percent. High = high stimulation intensity, low = low stimulation intensity.

Note that we did not include these details in the previous version since the Supplementary Information was already very dense. However, we agree with the reviewer that this information is crucial to justify our decisions.

Our pilot observations motivated the design of the main experiment, in which we implemented an event-related paradigm with an equal number of semantic matches and mismatches per condition and pre-specified a decomposition of behavioral and brain–behavior effects by trial type. Accordingly, the brain-behavior analysis consisted of a whole-brain regression analysis between activity and behavior, which was corrected for multiple comparisons with the family-wise error correction (FWE) at the cluster-level.
We have clarified this decision on pages 12-13: "A whole-brain cluster-level FWE-corrected regression analysis revealed that stronger upregulation of the domain-general right MFG was associated with less stimulation-induced reaction time delays, potentially reflecting compensatory increases outside the core lexico-semantic network (x, y, z = 36, 16, 54; t = 4.62, p = 0.038; FWE-corrected, Figure 2C) during semantic mismatch trials."

Finally, we performed a planned correlation analysis between activity changes at individual stimulation targets and reaction times separately for the trial types semantic matches and mismatches.

Since the decomposition by match vs mismatch trials was pre-specified based on our pilot data and constituted planned analyses rather than post-hoc exploration, we did not apply an additional correction across the two trial types. We tried to clarify this rationale during the previous revision round (page 34): "This was a planned decomposition analysis, based on differences in behavioral responses from our TMS pilot. Since we restricted our analysis to a small family (n=2) of planned, hypotheses-driven comparisons, we did not perform multiple comparison correction for whole-brain regression models and correlation analyses at the stimulation site, consistent with recommendations by Rothman (1990) and recent complementary suggestions (Boulesteix and Hoffmann 2024; García-Pérez 2023; Hooper 2025)."

Third, the "exploratory model" referred to in our Methods section pertains exclusively to additional analyses of physiological (fMRI) effects, without behavioral covariates. Our expectation, informed by the pilot experiment, was that TMS would have qualitatively different effects on behavior for matches vs mismatches. We did not assume that these behavioral differences would be mirrored in identical ways in the BOLD signal, which is consistent with the moderate correlations between brain activity and behavior.

To avoid confusion, we now explicitly distinguish between the planned brain-behavior model that decomposes effects by match vs mismatch, and the separate exploratory fMRI-only model; this distinction is now clearly labeled in the revised Methods (page 32) and Supplementary Results (page 53):

"For exploratory purposes, an additional second-level fMRI model was estimated that incorporated matches vs mismatches as a third factor (SI Figure 9)."

"The exploratory three-way interaction of intensity, task and match condition was not significant."

In line with the other reviewers' suggestions, we have further toned down our conclusions regarding behavioral compensation and now explicitly state in the new Limitations section that the brain-behavior findings should be viewed as converging, but not definitive, evidence (page 26):

"[...] Third, although we included a relatively large sample compared to typical interleaved TMS-fMRI studies of cognition (cf. Schuler and Hartwigsen, 2025), the observed brain-behavior correlations should be interpreted as converging rather than definitive mechanistic evidence for compensatory processes. Future studies should further examine the functional relevance of putative compensatory upregulation."

Reviewer Comment 2:
Semantic vs linguistic processing. MTG, the stimulation site, may relate to either linguistic processing or semantic control. Thus, when it is disrupted, either linguistic or semantic processing may be affected, which would have implications for different symptom profiles in aphasia. The authors respond to our original framing concern by stating that semantic processing can take place over either linguistic or non-linguistic stimuli - which is true but it doesn't address our concern that the manipulation may be targeting language processing upstream of semantic processing (a parallel example for images: it's like blurring the image before semantic processing takes place, in which case we would also expect the involvement of domain-general fronto-parietal areas to cope with increased task difficulty). If the authors are committed to a theoretical view that semantic and linguistic processing are inseparable, they should be very explicit about their theoretical commitments and outline their implications for result interpretation. Alternatively, they should clarify that their manipulation may target language processing more generally rather than only semantic control.

<u>Response:</u> *We thank the reviewers for raising this important conceptual point and for the helpful image-blurring analogy, which clarified the specific concern for us. We agree that our manipulation cannot fully rule out effects on processes upstream of semantic access, and we address this from three complementary angles: stimulation timing, the functional-anatomical profile of the stimulation site, and our task design.*

First, we selected a stimulation onset of 200 msec post word-onset based on our pilot data. This latency occurs after the earliest stages of visual/orthographic word-form encoding, which should be largely resolved within the first 150 ms and are typically associated with ventral occipito-temporal cortex rather than pMTG. We therefore believe our design makes a purely low-level perceptual account—directly analogous to image blurring—less likely, although

lexical selection processes may still be ongoing at 200 msec (e.g., Indefrey and Levelt, 2004; Wagner, Toffanin & Başkent, 2016).

Second, independent of timing, pMTG is not typically implicated in low-level visual or phonological input processing in the broader neuroimaging and lesion literature but has instead been consistently associated with lexico-semantic retrieval and semantic control (e.g., Whitney et al., 2011; Davey et al., 2015; Jefferies, 2013).

Third, our difficulty manipulation was specifically designed to increase demands on semantic selection and control—i.e., competition between candidate meanings—rather than properties associated with earlier processing stages such as word frequency, orthographic complexity, or word-form ambiguity. The fact that TMS effects were modulated by this semantic difficulty manipulation provides converging, though not definitive, evidence that the functionally relevant disruption occurs at the level of controlled lexico-semantic retrieval rather than purely upstream linguistic processing.
Taken together, we believe these considerations make a purely pre-semantic, "blurring"-type account of our findings unlikely, although we cannot fully exclude some contribution of upstream lexical processes, which are not fully dissociable from semantic retrieval in the present paradigm.
In accordance with the reviewers' suggestion from the previous review round, we have adopted the term "lexico-semantic" consistently throughout the manuscript to make this residual ambiguity explicit, and we have added the following paragraph to the Limitations section of the Discussion (page 26):

"Finally, although stimulation was delivered at 200 msec post word-onset—after the earliest stages of visual and orthographic word-form encoding are largely resolved—and although pMTG is not typically implicated in low-level perceptual or phonological input processing, we cannot fully exclude a contribution of upstream lexical retrieval processes that may still be ongoing at this latency. Our task-based difficulty manipulation, which specifically targeted semantic selection demands, supports an interpretation centered on (lexico-)semantic control rather than purely pre-semantic processing; however, we acknowledge that lexical and semantic retrieval processes are not fully dissociable in the present paradigm, and this should be considered when generalizing our conclusions."

Reviewer Comment 3:
The expected response pattern for the semantic control network (SCN) and multiple-demand network (MDN) across the experimental conditions (semantic/perceptual x high/low). For example, in the reply, the authors mentioned that semantic control was manipulated by high vs. low stimulation. However, in Figure 5, we see stronger overlap with the SCN for the Sem_gt_Perc map rather than the High_gt_Low map. The authors should clarify more clearly the distinction between the left and right panels of Figure 5A and explain what each result is intended to demonstrate. For example, because the paradigm was adjusted for difficulty, we may not expect to observe a traditional high_gt_low difficulty effect in the perceptual conditions within the MD system. Domain-general regions would instead be reflected by comparable responses to semantic and perceptual conditions, whereas more semantic-selective regions would show stronger responses to semantic conditions, as shown in Figure 5.

Response: *We thank the reviewers for their suggestions to clarify this point. As discussed in the previous revision round, multiple control conditions are necessary to fully depict TMS effects on task processing. While the Sem_gt_Perc high map separates TMS effects during lexico-semantic processing from peripheral TMS effects and all cognitive processes that are part of task processing but not of our interest, the semantic High_gt_Low map explains the interaction between TMS and lexico-semantic processing, while subtracting most of the activity that is directly associated with (lexico-)semantic control.*

We have clarified in the Results section what the different contrasts explain (page 17):
"The semantic high vs perceptual high contrast measured the TMS effect on lexico-semantic processing, while controlling for peripheral effects of high-intensity stimulation, symbol processing, and decision making. This contrast overlapped with the SCN at the bilateral IFG, SMA and pMTG, and with the MDN at the left MFG, SPL and SMA. Furthermore, there was overlap with the MDN for the semantic high vs semantic low contrast in the right MFG and left SPL. The latter contrast measured the TMS effect during lexico-semantic processing while partly controlling for peripheral effects and the direct effect of lexico-semantic decisions."

We further added a sentence clarifying the specificity of our TMS effects in terms of modulation of domain-general areas (page 19):

"Note that activity increases in the MDN under effective high-intensity stimulation were only observed for the semantic condition, highlighting the specificity of our effect."

Reviewer Comment 4:
Relatedly, the authors did not directly address the concern regarding task difficulty. The authors stated that "our difficulty manipulation was the TMS-induced modulation (high vs low stimulation intensity) during task processing while keeping task difficulty (stimulus presentation) constant." However, the behavioral results still show a significant task effect. The equivalence tests are conducted between high vs. low stimulation within each condition, but the main comparison also involves semantic vs. perceptual conditions.

Response: *We agree with the reviewers that the effect in the semantic high vs perceptual high contrast might have been partly driven by task difficulty. We acknowledge this now in the Limitations section (page 26):*

"Second, reaction times were generally faster in the perceptual condition, suggesting that domain-general activity changes for the semantic high vs perceptual high contrast might be partly driven by differences in task difficulty."

Reviewer Comment 5:
Finally, we still have methodological concerns regarding the ROI analysis. The authors provided subject-specific fROI analyses, but if we understand correctly, they did not use independent data for ROI definition and response extraction. In addition, the fROIs appear to have been defined using an omnibus contrast across all conditions. In general, this analysis remains unclear to me. For example, it is not clear what contrast is being referred to in the following sentence: "For every subject and each MDN/SCN cluster, we sampled the localizer t-statistic image and first applied a voxel-wise threshold of $p < 0.001$, uncorrected, to remove weak or noisy responses (Nieto-Castañón and Fedorenko 2012)."

<u>Response:</u> We thank the reviewers for raising this concern. We have defined fROIs based on the four conditions (semantic high, semantic low, perceptual high, perceptual low) of our main experiment. We opted for this contrast, since it provides information on both–modulations in multiple demand regions and semantic control regions–without being biased by one specific task condition. Furthermore, while our localizer data was acquired with a standard MR headcoil, the interleaved TMS-fMRI data were recorded with a dedicated coil setup. Therefore, we based our ROI extraction on data acquired with the same setup to avoid bias from the coil setup. For the reviewers' reference, we have calculated an additional fROI analysis based on our localizer session (depicted below; ***Revision Figure 1****). Results converge with both of our previous analyses: data-independent and fROI extraction.*
We updated the description of our fROI methodology as follows (page 45 of the Supplementary Information):

"Subject-specific functional localization was based on an effects-of-interest contrast that depicts the combined effect across all task conditions (semantic high, semantic low, perceptual high, perceptual low) of our main experiment. We selected this contrast to minimize bias toward a specific task condition and to obtain robust localization of regions associated with multiple demand and semantic control using our dedicated interleaved TMS-fMRI coil setup. For every subject and each MDN/SCN cluster, we applied a voxel-wise threshold of p < 0.001, uncorrected, to this contrast to remove weak or noisy responses (Nieto-Castañón and Fedorenko 2012)."

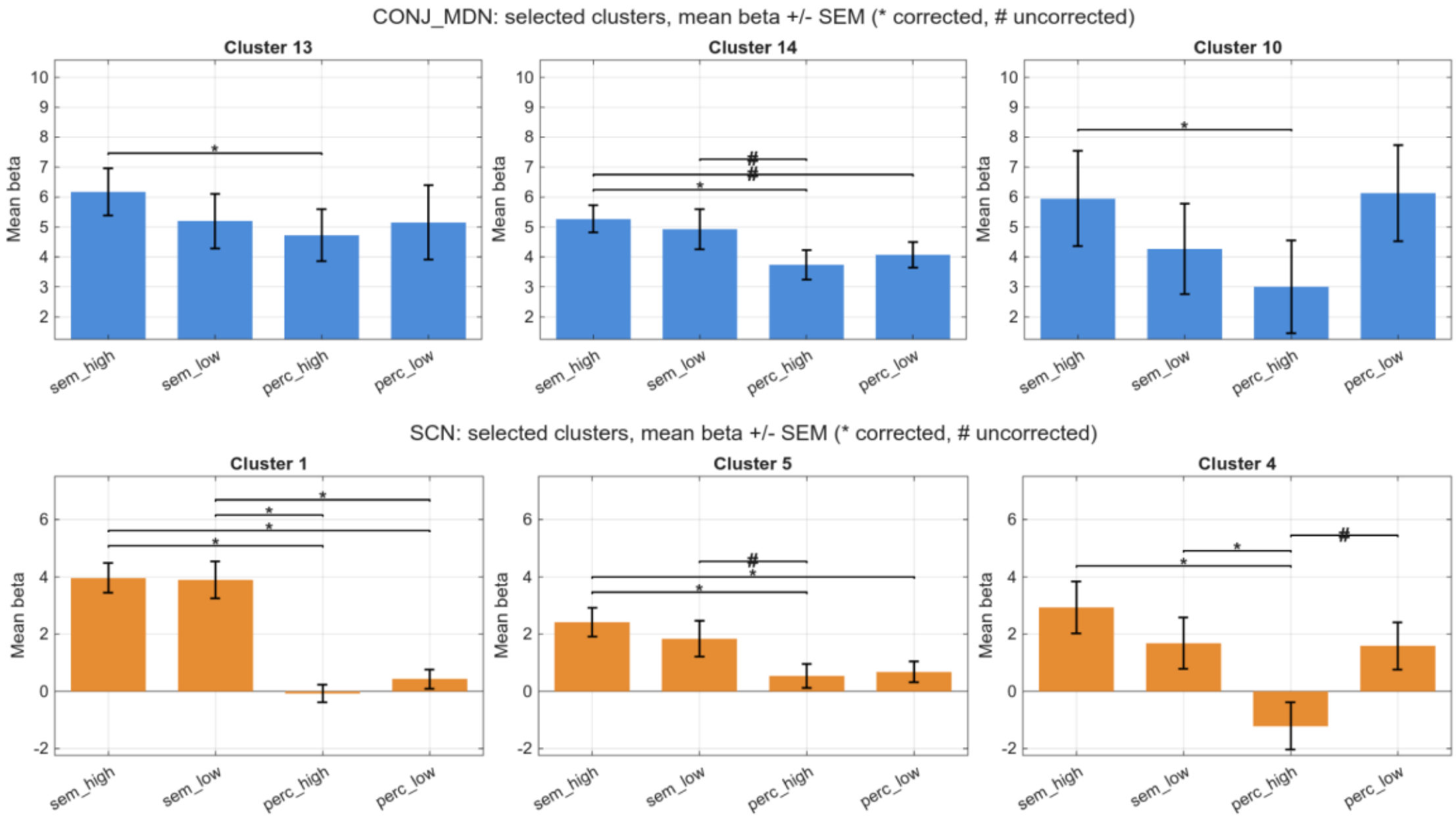

Revision Figure 1. Subject-specific fROI-analyses for MDN and SCN overlap in selected clusters. Comparison within MDN network-mask thresholded at t > 2 (MDNt2). MDN (blue) cluster 14 = SMA, MDN cluster 10 = right DLPFC, MDN cluster 13 = left MFG, SCN (orange) cluster 1 = left IFG, SCN cluster 4 = right IFG, SCN cluster 5 = SMA. For the MDN, fROIs were based on the conjunction of semantic > baseline and perceptual > baseline from our localizer session. For the SCN, fROIs were based on the semantic > perceptual contrast from our localizer session.

Minor points:

Reviewer Comment 6:
- The authors noted that, when running gPPI, the task regressor was not convolved with the HRF, which is not standard practice (McLaren et al., 2012).

<u>Response:</u> *We opted not to perform HRF convolution as suggested and implemented for sparse sampling approaches considering the volume gaps in our EPI data to avoid TMS artifacts (Nieto-Castañón & Whitfield-Gabrieli, 2021). We have added the following clarification to the Methods section (page 33):*

"Four task conditions were modeled as miniblocks without HRF convolution as implemented in the toolbox to account for acquisition gaps (semantic high, semantic low, perceptual high, perceptual low; Nieto-Castañón & Whitfield-Gabrieli, 2021)."

Reviewer Comment 7:
- In the Discussion, the authors state: "In contrast, the right MFG and PMd were both upregulated across high stimulation intensity semantic and perceptual conditions, consistent with a role in domain-general difficulty and performance monitoring (Quillen et al. 2021)." However, if we are reading the figure correctly, there does not appear to be an intensity effect in the perceptual conditions for rMFG and rPMd.

<u>Response:</u> *Thank you for spotting this inconsistency. We have rephrased this sentence to clarify that there was no TMS effect during the perceptual condition (page 23):*

"In contrast, the right MFG and PMd showed increased activity during high compared with low stimulation intensity in the lexico-semantic task, but comparable activity across both stimulation intensities in the perceptual task (no significant intensity effect), consistent with their role in domain-general difficulty and performance monitoring (Quillen et al. 2021)."

<u>Reviewer #4</u>
The authors have done an excellent job addressing the concerns raised in the initial review process. The manuscript is substantially improved in clarity, rigor, and interpretation, and overall I found this to be a technically sophisticated and conceptually important study. The use of interleaved TMS-fMRI to probe acute network adaptation during semantic processing is especially compelling, and the manuscript now provides a much clearer account of how semantic and domain-general systems interact under perturbation.

The revision thoughtfully addresses many of the key methodological and interpretational concerns from the previous round of review. In particular, I appreciated the expanded justification of the control conditions, the clearer distinction between planned and exploratory analyses, and the more balanced interpretation of the "compensation" findings. The additional statistical reporting (e.g., bootstrap confidence intervals, leave-one-out analyses) also strengthens confidence in the reported effects.

The manuscript now does a much better job situating the findings within broader models of semantic control and multiple-demand network engagement. I also thought the new discussion

emphasizing a shift from "where" to "how" in semantic network neuroscience was especially strong and nicely framed the broader significance of the work.

Overall, I think this is a strong and innovative manuscript that makes a valuable contribution to the literature on semantic cognition, adaptive network dynamics, and interleaved TMS-fMRI methodology. The authors responded carefully and professionally to the prior critiques, and I believe the manuscript is now suitable for publication pending minor revisions:

Response: *We thank the reviewer for their positive evaluation of our manuscript and for helping us improve our paper. We have addressed their remaining suggestions below.*

Reviewer Comment 1:
I encourage the authors to continue using somewhat cautious language when referring to "compensation." The manuscript is much improved in this regard, but there are still a few places where the wording implies stronger causal evidence for compensation than the data fully support. In several instances, phrasing such as "putatively compensatory" or "associated with preserved performance" may better match the level of inference supported by the data.

Response: *We thank the reviewer for this suggestion. Reviewer #2 and #3 had similar comments. We have revised the manuscript to tone down our statements regarding compensatory recruitment and avoid overinterpretation of our findings.*

Reviewer Comment 2:
The distinction between semantic match and mismatch trials is now better justified, particularly with the clarification that this decomposition was motivated by pilot data. I think the manuscript would benefit from a brief conceptual discussion of why matches and mismatches may recruit partially different control mechanisms or processing strategies.

Response: *We thank the reviewer for this recommendation. We have added the following to our Methods section (page 28):*

"Conceptually, semantic match trials primarily engage convergent retrieval and verification of appropriate associations, whereas semantic mismatch trials place greater demands on conflict monitoring, divergent lexico-semantic search, and the inhibition of inappropriate links, thereby recruiting partially distinct control mechanisms and processing strategies (Tik et al. 2018; Gao et al. 2022; Kuhnke et al. 2023)."

Reviewer Comment 3:
The gPPI connectivity findings are interesting and potentially important. I would just encourage slightly cautious wording regarding network "reconfiguration" or mechanistic interpretation of connectivity directionality, given the limitations of gPPI analyses.

Response: *We agree with the reviewer and have modified the wording regarding the interpretation of our gPPI results in the Discussion as follows (page 21):*

"By contrast, upregulation of an adjacent rMFG subregion and its increased functional coupling with the pre-SMA—a hub at the interface of semantic and domain-general control—likely

reflects network modulation consistent with potential compensation following transient disruption of semantic processing (Chiou et al. 2023).”

Reviewer #5

I co-reviewed this manuscript with one of the reviewers who provided the listed reports. This is part of the Nature Communications initiative to facilitate training in peer review and to provide appropriate recognition for Early Career Researchers who co-review manuscripts.

Response: *We would like to thank you again for the time and effort invested in the review of our manuscript.*

References

Davey, J., Cornelissen, P. L., Thompson, H. E., Sonkusare, S., Hallam, G., Smallwood, J., & Jefferies, E. (2015). Automatic and controlled semantic retrieval: TMS reveals distinct contributions of posterior middle temporal gyrus and angular gyrus. *Journal of Neuroscience*, *35*(46), 15230-15239.

Indefrey, P., & Levelt, W. J. (2004). The spatial and temporal signatures of word production components. *Cognition*, *92*(1-2), 101-144.

Jefferies, E. (2013). The neural basis of semantic cognition: converging evidence from neuropsychology, neuroimaging and TMS. *Cortex*, *49*(3), 611-625.

Nieto-Castañón, A., & Whitfield-Gabrieli, S. (2021). CONN functional connectivity toolbox (RRID: SCR_009550), Version 21.

Wagner, A. E., Toffanin, P., & Başkent, D. (2016). "The timing and effort of lexical access in natural and degraded speech." *Frontiers in Psychology, 7*: 398.

Whitney, C., Kirk, M., O'Sullivan, J., Lambon Ralph, M. A., & Jefferies, E. (2011). The neural organization of semantic control: TMS evidence for a distributed network in left inferior frontal and posterior middle temporal gyrus. *Cerebral Cortex*, *21*(5), 1066-1075.
